# Structural basis of the gating mechanism of the large-conductance mechanosensitive channel from *Escherichia coli*

**DOI:** 10.1038/s41467-026-76193-0

**Published:** 2026-08-22

**Authors:** Katie Hardman, Joshua L. Wort, Qaiser Waheed, Xinyu Liu, Diya Arul, Sebastian A. Porav, Antonio N. Calabrese, Stephen P. Muench, Christos Pliotas

**Affiliations:** 1 Astbury Centre for Structural Molecular Biology, University of Leeds, Leeds, UK; 2 School of Biomedical Sciences, Faculty of Biological Sciences, University of Leeds, Leeds, UK; 3 BioEmPiRe Centre for Structural Biological EPR Spectroscopy, School of Biological Sciences, Faculty of Biology, Medicine and Health, The University of Manchester, Manchester, UK; 4 Manchester Institute of Biotechnology, The University of Manchester, Manchester, UK; 5 Leeds Institute of Cardiovascular and Metabolic Medicine, LIGHT Laboratories, University of Leeds, Leeds, UK; 6 School of Molecular and Cellular Biology, Faculty of Biological Sciences, University of Leeds, Leeds, UK

**Keywords:** Cryoelectron microscopy, Membrane biophysics, Membrane proteins

## Abstract

The mechanosensitive channel of large conductance (MscL) is a tension-gated, pore-forming protein that acts as a safety valve to protect bacteria from osmotic lysis. *Escherichia coli* MscL (EcMscL) was the first mechanosensitive channel discovered and subsequently served as a model system for understanding mechanical sensing, becoming one of the most decorated and well-studied systems. Despite extensive biophysical and functional characterisation spanning several decades, the precise mechanism of EcMscL gating has been poorly understood due to the lack of high-resolution structural information. Herein, we solve two EcMscL structures by cryoEM in the closed conformation in DMPC and DOPC lipid nanodiscs. Using PELDOR/DEER spectroscopy, we screen conditions and identify that in DSPC lipids, the EcMscL conformational ensemble shifts away from the closed state and that open-like states are present. We solve the structure in an expanded state by cryoEM, revealing an architecture with pore properties consistent with previous electrophysiology reports. By combining hydrogen-deuterium exchange mass spectrometry and molecular dynamics simulations, we investigate the dynamics of EcMscL gating in lipid bilayers, identifying sites involved in the closed-to-expanded transition. Combined, this enables us to inform on the elusive structural mechanism of EcMscL mechanosensitive channel function.

## Abstract (web summary)

Here, authors solve three cryo-EM structures of EcMscL in two different lipid environments and two distinct conformational states; closed and open-like, guided by EPR. Additional EPR, MD and HDX-MS experiments provide insight into the gating mechanism.

## Introduction

Mechanotransduction, the translation of mechanical force into intracellular signals, is a conserved mechanism present in cells across all life forms^1^. Prokaryotic mechanosensitive (MS) channels function as osmoprotectants, altering their conformation in response to high membrane tension to facilitate the passive flow of ions and solutes. In higher organisms, mechanotransduction mediates many important physiological processes, including the detection of auditory, haptic, and nociceptive stimuli, blood pressure regulation and turgor control^2^. Pressure-sensing channels were first discovered by patch-clamp electrophysiology on the plasma membrane of giant *Escherichia coli* (*E. coli*) spheroplasts^3^. These channels were later identified as the MS ion channels of large- (MscL)^4,5^ and small- (MscS)^6^ conductance, respectively.

The MscL from *E. coli* (EcMscL) has served as a paradigm for studying mechanosensation across all species for over four decades, yet the three-dimensional structure of the wild-type, full-length protein has never been resolved, owing to its recalcitrance to crystallisation^7^, and the low molecular weight limitations of cryogenic electron microscopy (cryoEM). The first X-ray crystal structure of MscL, derived from a construct expressing the *Mycobacterium tuberculosis* (MtMscL) orthologue, was solved to 3.5 Å resolution in detergent^8,9^, revealing a homo-pentameric channel. Each subunit comprises an N-terminal short helix (S1) positioned parallel to the membrane, two transmembrane (TM) α-helices (TM1 and TM2) connected by a periplasmic loop, and a C-terminal α-helix that projects into the cytoplasm. Overall, the channel is divided into two distinct domains: (i) a TM domain (TMD) and (ii) a cytoplasmic helical bundle (CTD)^8,9^.

The MtMscL structure was used for homology modelling of EcMscL to facilitate interpretation of computational, biochemical and biophysical observations, and to rationalise the design and engineering of a wide range of EcMscL-inspired biotechnological applications^10,11^. Homology modelling was leveraged on the 50% sequence similarity between these two channels, despite significant functional differences. MtMscL exhibits a higher tension activation threshold than EcMscL, can be activated by lysolipids, and shows a distinct lipid preference^12^. Direct structural comparison within the channel’s tension-sensitive pockets by pulsed electron–electron double resonance (PELDOR; also known as double electron-electron resonance, DEER) spectroscopy revealed significant structural differences between EcMscL and MtMscL, accounting for their distinct functional behaviours^13,14^.

The structures of MscL from *Staphylococcus aureus* (SaMscL) and the archaeon *Methanosarcina acetivorans* (MaMscL) have also been determined by X-ray crystallography^15,16^. SaMscL was initially reported to form a homotetramer^15^; however, subsequent biochemical and mass spectrometry studies suggested that SaMscL’s tetrameric organisation may be an artefact arising from the detergent used during solubilisation^17,18^. A C-terminally truncated SaMscL structure revealed an expanded conformation, with TM1 and TM2 tilted outward relative to MtMscL, resulting in membrane-spanning regions 13 Å thinner and 17 Å wider than MtMscL^8,9,15^, respectively. Similar expanded states were later observed in MaMscL by crystallography^16^ and in MtMscL in lipid membranes by PELDOR^13^, supporting the helix-tilt model for gating, in which TM1 and TM2 tilt and radially expand as the pore expands^19,20^. Single-molecule Förster resonance energy transfer (smFRET) measurements indicated that EcMscL’s pore may reach 28 Å wide^21^, signifying one of the largest known pores in all ion channels^1^. While the CTD is highly conserved among MscL orthologues, its function remains unclear. To account for the channel’s exceptionally large conductance (>3 nS), several models have proposed that the CTD helices dissociate during channel gating^19,22–24^. However, biochemical studies have demonstrated that stabilising the CTD through covalent cross-linking does not alter gating parameters, whereas destabilising mutations do^25,26^. These observations support a model in which the CTD functions as a “molecular sieve”, helping retain vital metabolites during osmotic shock^25,26^. Furthermore, the crystal structure of the isolated EcMscL CTD, solved at 1.45 Å resolution, revealed a tightly packed pentameric helical bundle even in the absence of membrane anchoring^27^.

Lipids play a crucial role in the gating of MS channels. MscL channels have lipid-binding pockets at the cytoplasmic interface formed by the horizontal S1 helix and adjacent TM helices^8,9,28,29^. Blocking lipid access to this pocket stabilises an expanded state^13^, analogous to the natural, tension-induced sub-conducting state S1^30^. Similar pockets have been identified across diverse MS channel families. These include the prokaryotic channels MscS^31–34^, YnaI^35^, YbiO^36^, and MscK^37^; the plant channels Msl1^38^ and Flyc1^39^; fungal MscA/B^40^, with comparable fenestrations in eukaryotic two-pore domain potassium (K2P) channels^41–43^. For MS channels, lipid dissociation from hydrophobic pockets drives a conformational change to the open state. This entropy-driven “lipid-moves-first” model^44,45^ provides a molecular framework for the general force-from-lipid principle^46^. Membrane properties, such as lipid composition, bilayer thickness and fluidity, also influence MS channel activity. For example, MscL exhibits a lower activation threshold when reconstituted into lipids with shorter acyl chains^20,47^, due to an increase in hydrophobic mismatch and the exposure of hydrophobic residues^48,49^. Cyclodextrin (CD) treatment of nanodisc-reconstituted MscS^50,51^ and EcMscL^52^ has been used to induce MS channel expansion, the extent and kinetics of which depend on incubation time and the CD-to-lipid ratio used to sequester lipids from nanodiscs^51^.

Here, we present cryoEM structures of EcMscL in two distinct conformations, closed and expanded, stabilised in different lipid environments. Structure determination of small membrane proteins by cryoEM remains a significant challenge. Notably, unlike some cryoEM structures of small proteins that employ Fabs^53^, nanobodies^54^, or engineered scaffolds^55^ to increase molecular weight, reduce flexibility and improve particle alignment^56^, here, EcMscL was resolved in two distinct states without the use of state-locking antibodies. Using a multidisciplinary approach to explore EcMscL’s conformational ensemble in different lipid membranes, guided by PELDOR spectroscopy and complemented by hydrogen-deuterium exchange mass spectrometry (HDX-MS) and atomistic molecular dynamics (MD) simulations, we provide key insights into the structural basis of the gating transition of this essential and first-discovered MS channel.

## Results

**Fig. 1:**
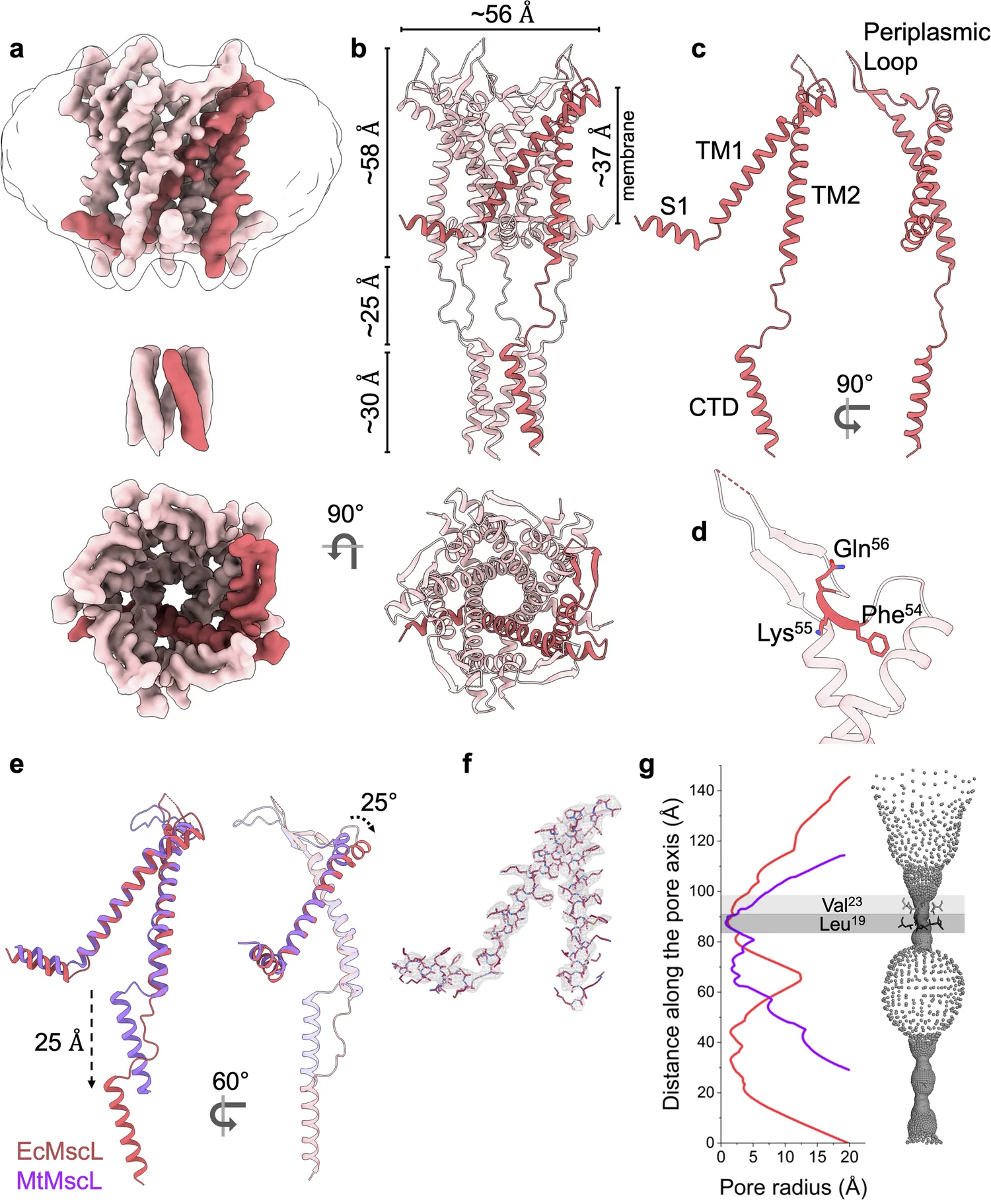
The cryoEM structure of closed-state EcMscL. **a** The 4.1 Å resolution density map of EcMscL in MSP1D1 and DMPC nanodiscs shown from side-on (top) and top-down (bottom) views. **b** The model of closed-state EcMscL showing approximate dimensions. **c** Each subunit of the pentameric channel includes an N-terminal short helix (S1), two TM helices (TM1 and TM2), connected by a periplasmic loop, a cytoplasmic loop and a cytoplasmic helix. **d** The periplasmic loop, including the part helical ω-loop, features residues Phe^54^, Lys^55^ and Gln^56^. **e** Comparison between one subunit of MscL from *E. coli* (pink; EcMscL) and *M. tuberculosis* (purple; MtMscL). TM1 in EcMscL is tilted by ~25° relative to MtMscL. **f** TM1 and TM2 side chains modelled in the density map. **g** Pore analysis of EcMscL (pink) and MtMscL (purple) using HOLE^59^.

Full-length EcMscL was reconstituted into nanodiscs composed of MSP1D1 and 1,2-dimyristoyl-sn-glycero-3-phosphocholine (DMPC; 14:0) or 1,2-dioleoyl-sn-glycero-3-phosphocholine (DOPC; 18:1) lipids, and the structure was resolved to 4.1 Å resolution by cryoEM (Fig. 1a). Before reconstitution, the lipid-to-protein ratio was estimated using CHARMM-GUI^57^, ensuring that multiple lipid rings encapsulated the channel to minimise artefacts from MSP-protein interaction or MSP-induced compression. In both DMPC (Fig. 1 and Supplementary Fig. 1 for cryoEM processing pipeline) and DOPC (Supplementary Figs. 2 and  3 for cryoEM processing pipeline) nanodiscs, EcMscL forms a homopentameric channel, with each subunit comprising an N-terminal short helix (S1), two TM α-helices (TM1 and TM2) connected by a periplasmic loop, a cytoplasmic loop and a C-terminal α-helix (Fig. 1b, c). Our model features well-defined density for TM1 and TM2 side chains, which confirms the register of the helices (Fig. 1f). The TMD of EcMscL spans ~56 Å at the widest diameter of the periplasmic surface and is ~58 Å in length (Fig. 1b). The TMD is contained entirely within the nanodiscs, except for the periplasmic loops, which protrude slightly from the disc (Fig. 1a). The position of the CTD is the most dramatic feature of the structure of EcMscL, as it is situated ~25 Å away from the TMD (Fig. 1b). The 10-amino-acid loop connecting the C-terminus of TM2 to the CTD is proline-rich (Pro^109^, Pro^113^, Pro^115^), reinforcing its stability. 3D variability analysis in CryoSPARC^58^ revealed minimal conformational heterogeneity between the TMD and CTD, suggesting their relative positions are fixed. However, some degree of flexibility is likely between the regions, as the cytoplasmic loops are only visible in the density map at a high threshold (Supplementary Fig. 4). The crystal structure of the isolated EcMscL CTD^27^ was used to model this region of the full-length protein (Supplementary Fig. 4c).

The structure of EcMscL in DMPC and DOPC lipid nanodiscs presents substantial differences compared to the X-ray crystal structure of MtMscL solved in detergent^8,9^. Whilst the TMD helices of EcMscL—S1, TM1 and TM2—align closely to those in its orthologue (Fig. 1e), the C-terminal end of TM1 in EcMscL is tilted by ~25° compared to the equivalent region in MtMscL, meaning that the diameter at the periplasmic surface is ~9 Å wider. The periplasmic loop in EcMscL is six amino acids longer than in MtMscL, and while both proteins contain a short β-hairpin, EcMscL also features a partial helical segment, known as the ω-loop in MaMscL^16^, formed by Phe^54^, Lys^55^ and Gln^56^ (Fig. 1d). On the cytoplasmic side, the loop between TM2 and the CTD is twice as long in EcMscL (residues 107–115) compared to MtMscL (residues 101–104), but the CTD in EcMscL sits ~25 Å away from the membrane, whereas in MtMscL it is aligned with the TMD (Fig. 1e). The extended length of the cytoplasmic loop in EcMscL was predicted from sequence analysis, but its orientation relative to the protein was unexpected. HOLE analysis^59^ of EcMscL in DMPC (Fig. 1g) revealed a pore radius of ~1 Å at the narrowest point, consistent with a non-conducting, closed-state channel. Comparison with MtMscL showed minimal pore differences, with nearly identical distances measured between the constriction sites (Leu^19^/Val^23^ in EcMscL and Leu^17^/Val^21^ in MtMscL) (Fig. 1g). Notably, the pore-lining residues at the cytoplasmic side of TM1 that form the channel gate are highly conserved among MscL orthologues^60^.

### Scanning the EcMscL conformational ensemble in membranes by PELDOR

Having determined the closed-state structure of EcMscL in two lipid systems, we next sought to investigate conditions that promote channel gating and pore expansion. To this end, we employed PELDOR (or DEER) spectroscopy, which enables rapid, high-resolution screening of conformational ensembles, to identify conditions that may result in expanded states suitable for cryoEM analysis. We hypothesised that MscL gating is primarily influenced by (a) lipid phase and order, and (b) nanodisc size. Therefore, EcMscL was reconstituted into lipids with a high phase transition temperature (*T*_m_) (1,2-distearoyl-sn-glycero-3-phosphocholine; DSPC; 18:0) and nanodisc scaffolds of varying belt sizes (MSP1D1 and MSP1E3D1), in pursuit of populations with enhanced expanded-state conformations. As in cryoEM, we used MSP scaffolds that provided at least three lipid layers around EcMscL, thereby diminishing (if any) MSP-induced artefacts.

**Fig. 2:**
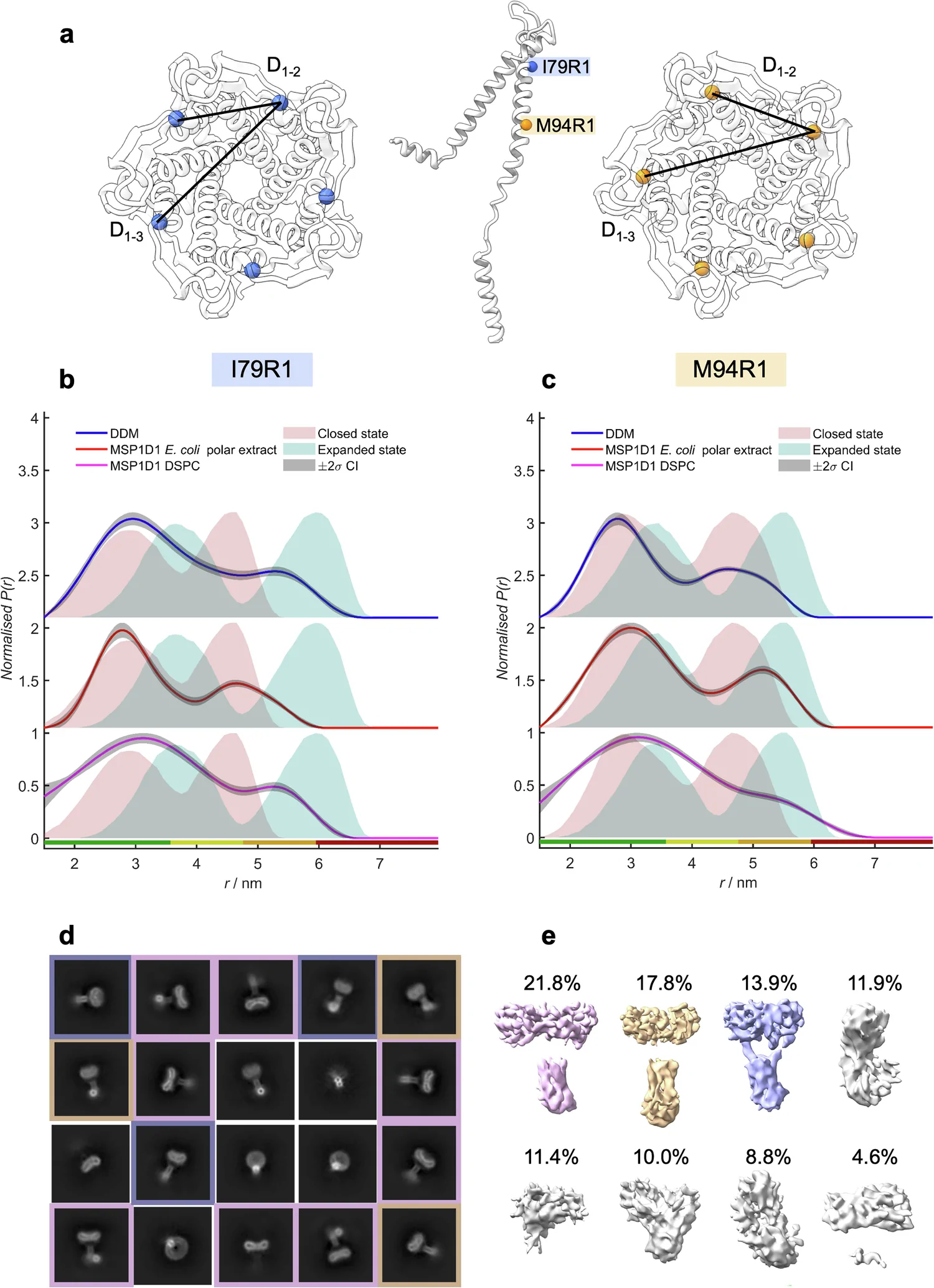
Monitoring the EcMscL conformational ensemble in lipid membranes by PELDOR (or DEER) spectroscopy. **a** Spin labelling sites Ile^79^R1 (blue) and Met^94^R1 (orange) on TM2 of EcMscL. Due to the channel’s pentameric arrangement, distances between neighbouring (D_1–2_) and opposing (D_1-3_) subunits are measured by PELDOR. **b**, **c** Different conditions stabilise distinct conformations of EcMscL. PELDOR distance distributions in DDM (blue) and reconstituted into MSP1D1 nanodiscs inflated with either *E. coli* polar extract lipids (red) or DSPC (magenta) for Ile^79^R1 (**b**) and Met^94^R1 (**c**). Shaded area (grey) represents distribution uncertainty, whilst the coloured bars indicate reliability. The green region denotes reliable shape, width and mean; yellow denotes reliable width and mean; orange indicates only mean reliability; and red indicates that no quantification is possible. Filled areas (salmon and teal) depict predicted distance distributions for the EcMscL closed- (in MSPD1/DMPC nanodiscs) and expanded- (in MSP1D1/DSPC nanodiscs) state(s), respectively. In silico distance distribution modelling was performed using MtsslWizard^117,118^. PELDOR data were processed using global fitting and compactness regularisation in DeerLab^109,110^. **d** Selected 2D class averages for EcMscL in MSP1D1/DSPC nanodiscs show a conformationally heterogeneous population of expanded states. **e** The initial 3D classes (ab initio reconstruction without symmetry imposed) for these particles resulted in three EcMscL-like maps (pink, orange and purple) in a range of expanded states. Closed-state structures at sufficient abundance were not detected in either 2D or 3D classification.

PELDOR spectroscopy, which measures long-range distances (2–16 nm) between pairs of spin-labelled residues, is a powerful tool for probing the conformational equilibria of membrane proteins in solution and lipid-rich environments^61–66^. Here, two distinct sites on TM2 of EcMscL (Ile^79^R1 and Met^94^R1) were individually labelled to serve as reporters for detecting channel expansion (Fig. 2a), and the distance between labels in different conditions was measured by PELDOR spectroscopy (Fig. 2b). These cysteine-modified EcMscL mutants were chosen based on previous studies indicating that (1-oxyl-2,2,5,5-tetramethyl-Δ3-pyrroline-3-methyl) methanethiosulfonate (MTSSL) or 2-(trimethylammonium) ethyl methanesulfonate (MTSET) labelling on the same Cys sites did not disrupt channel function or cell viability^29,67,68^. Note that this results in five spin labels in each homopentamer, meaning that two inter-subunit distances (D_1-2_ and D_1-3_) are measured simultaneously, reflecting the distances between neighbouring and opposing subunits (Fig. 2a). Continuous-wave electron paramagnetic resonance (CW-EPR) spectroscopy measurements for the individual spin labels, as well as the raw PELDOR traces, are in Supplementary Fig. 5.

Under all conditions tested, distance distributions were broad, indicating conformational heterogeneity, consistent with multiple EcMscL channel states present in the ensemble (Fig. 2b, c and Supplementary Figs. 6–8). For comparison, previous measurements of Met^94^R1 EcMscL in DMPC^14^, the same lipid in which we resolved the EcMscL closed state, yielded a more homogenous distribution and compact channel configuration. Interestingly, the kinetics of the closed-S1 transition are on the same timescale (high ms) as the rate of flash-freezing of EPR samples in N_2(l)_, with S1–S4 transitions progressing faster^69^. This provides an explanation for the degree of conformational heterogeneity observed, as a mixture of closed- and all sub-conducting states is present. Indeed, a similar level of conformational heterogeneity is observed in 2D and 3D classes from cryoEM (Fig. 2d, e). We observe substantial deviation from the closed-state in silico distance distributions (Fig. 2b, c salmon fills; vide supra for cryoEM structure), which corresponds to the presence of a population of expanded-state(s) (Fig. 2b, c teal fills; vide infra for cryoEM structure) in the conformational landscape.

For both Ile^79^R1 and Met^94^R1 variants of EcMscL, in the native lipid environment (i.e., *E. coli* polar extract lipids), the distance distributions yielded the highest local and global similarity scores to the closed-state in silico distance distribution (Supplementary Tables 1–4). This is consistent with the expectation that under native *E.coli* lipid conditions, the EcMscL channel is predominantly in the closed-state conformation and suggests the nanodisc (MSP1D1 in this case) does not induce artificial expansion. However, reconstitution in DSPC shifted the conformational equilibrium away from the closed-state (i.e., yielded lower local and global similarity scores to the closed-state modelling, across technical replicates, distinct analysis pipelines, and irrespective of nanodisc size; see Supporting Tables 1–4 for details). Notably, PELDOR distance distributions in n-dodecyl-β-D-maltoside (DDM) yielded intermediate similarity scores to the closed-state in silico modelling.

In summary, the EPR-based screen showed that reconstitution of EcMscL in DSPC nanodiscs enhanced sampling of expanded conformations. We therefore reconstituted EcMscL into MSP1D1/DSPC nanodiscs for further single-particle cryoEM analysis.

### The structure of EcMscL in an expanded/open-like state

**Fig. 3:**
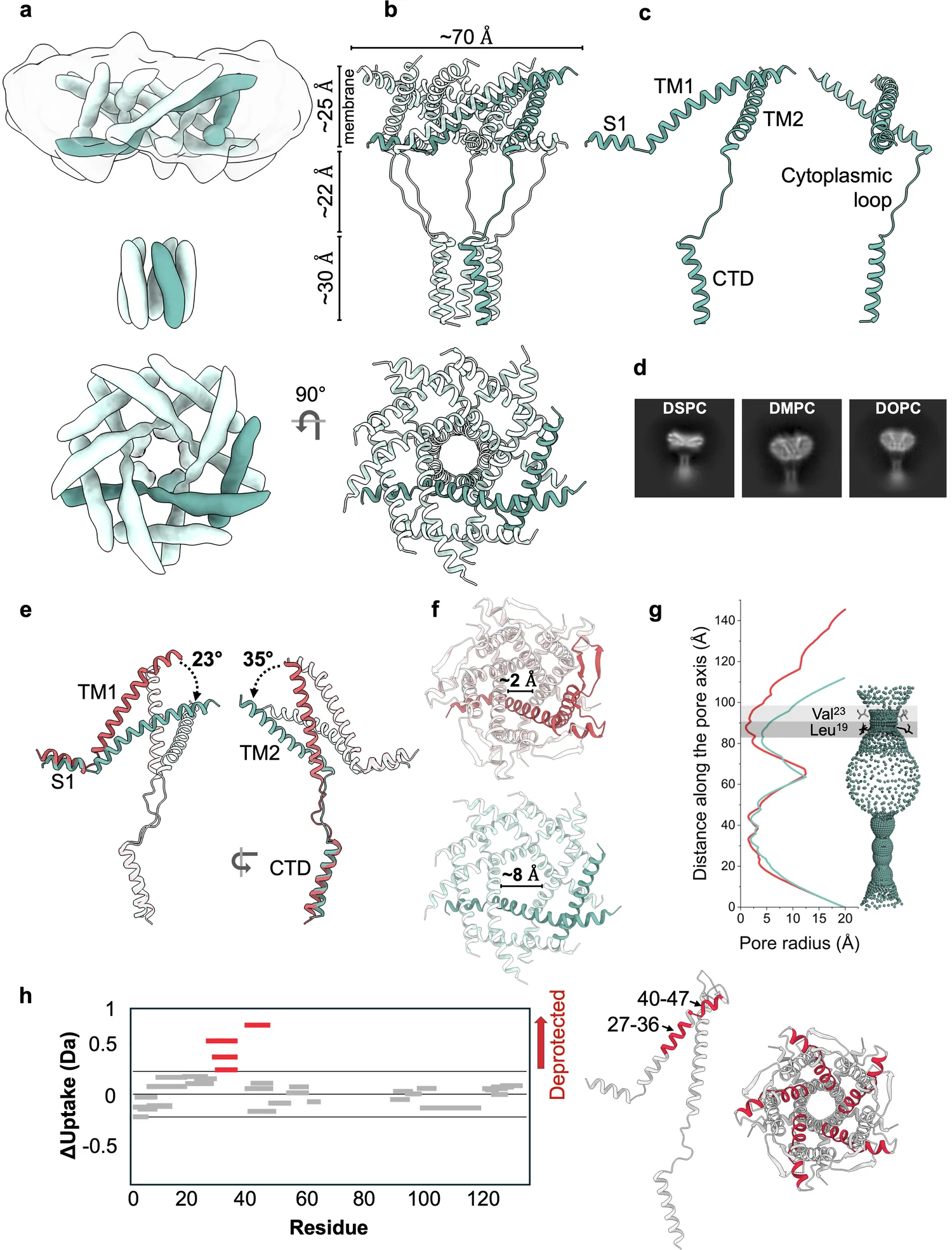
The expanded/open-like state structure of EcMscL. **a** The 7.0 Å resolution cryoEM density map of EcMscL in MSP1D1 and DSPC nanodiscs reveals an expanded conformation, with large flattening of the TMD. **b** The expanded state structure of EcMscL showing approximate dimensions. **c** One subunit of EcMscL shown from two viewing angles. **d** Side-view 2D classes of EcMscL in DSPC, DMPC and DOPC nanodiscs. **e** Comparison between one subunit of EcMscL in the closed and expanded state. In DSPC lipids (dark teal), TM1 and TM2 tilt towards the membrane plane by ~23° and ~35°, respectively. **f** Pore comparison of the TMD (residues 1–104) of EcMscL in the closed (salmon) and expanded state (teal). The pore diameter expands from ~2 Å to ~8 Å. Note that residues 105–136 (encompassing the cytoplasmic loops and CTD) have been removed to better visualise the pore region. **g** HOLE^59^ pore analysis of the closed and expanded states of EcMscL. **h** HDX-MS analysis of EcMscL in DMPC vs DSPC nanodiscs. Data are shown for the 30-second deuterium incubation time point. Deprotection in the same regions of the protein was observed at the other two deuterium incubation timepoints (5 and 30 min) analysed (see Supplementary Fig. 10). Statistical analyses (98% confidence limit, *p* value < 0.02) were applied to identify peptides with significant deuterium uptake (ΔUptake). Residues 27–36 and 40–47 of EcMscL (red) are deprotected in DSPC nanodiscs.

Compared to its structure in DMPC or DOPC lipids, EcMscL adopts an expanded conformation in DSPC lipids (Fig. 3 and Supplementary Fig. 9 for cryoEM processing pipeline). The diameter of ~8 Å is consistent with previously reported EcMscL sub-conducting state(s) S1/S2^69^. The 2D class averages of EcMscL in DSPC nanodiscs demonstrate a dramatically different conformational state compared to both DMPC and DOPC (Fig. 3d). This state is characterised by a large reduction in TMD thickness and a wider pore entrance, evident in both top-down and oblique side-view classes (Fig. 3d). Notably, the DSPC-stabilised state resembles the 2D class averages of CD-treated EcMscL^52^, suggesting that both DSPC bilayers and CD-treated bilayers are associated with lower lipid density, and may be used to stabilise similar states in other MS proteins.

A model of EcMscL in DSPC lipids was built into the 7.0 Å density map (Fig. 3b), allowing reliable positioning of the TMD helices. Residues 50–73, corresponding to the periplasmic loop, were omitted owing to insufficient resolution; however, all other regions, including the TMD helices, S1, TM1 and TM2, and the CTD, were modelled. Significant structural rearrangements in the TMD characterise channel expansion. In the expanded state in DSPC, the diameter at the periplasmic surface is ~12 Å wider than in the closed-state model. The length of the TMD within the membrane, along the *z* axis perpendicular to the membrane plane, is largely reduced, measuring ~25 Å in the DSPC-expanded state and ~37 Å in the closed state. In the expanded conformation, both TM helices tilt and radially expand, consistent with the helix-tilt gating transition proposed previously for EcMscL, based on computational and spectroscopic methods^19,20^. TM1 tilts by ~23° and TM2 by ~35°, relative to the central pore axis (Fig. 3e). As the channel expands, the N-terminal S1 helix tilts towards the membrane, increasing the angle between S1 and TM1 from ~100° to ~145°. The angle between TM1 and TM2 in the same subunit (TM1–TM2) increases from ~43° to ~63° as they cross perpendicularly. However, the crossing angle of TM1 and TM2 from neighbouring subunits (TM1–TM2’) decreases from ~18° to ~5°, reflecting a shift towards a parallel arrangement with respect to the membrane plane. The TM1–TM2’ interface is predominantly composed of hydrophobic residues, which likely stabilise this parallel interaction as the channel expands. Channel expansion is associated with an increase in pore diameter from ~2 Å to ~8 Å (Fig. 3f, g). Due to the outward displacement of TM2, the CTD shifts ~5 Å closer to the membrane, equivalent to one full α-helix turn (Fig. 3e). Interestingly, the CTD moves as a single, assembled pentameric subunit during channel expansion^27,70,71^. In contrast to earlier models proposing its dissociation and lateral repositioning to the membrane plane^19,20,72^, the CTD appears to remain mostly intact. The extended cytoplasmic loops create sufficient space between domains to permit the passage of large molecules through the inter-loop gaps, consistent with previous reports^26,73^.

### The EcMscL periplasmic region becomes exposed during channel expansion

Having determined the structures of EcMscL in both closed- and expanded-states by cryoEM, we sought to explore the protein domains involved in these transitions. To this end, we employed HDX-MS, a powerful tool for studying membrane protein dynamics^74–77^, including MtMscL^30,78^. Here, we compared the extent of deuterium incorporation in EcMscL reconstituted into MSP1D1 nanodiscs containing either DMPC or DSPC, to match the lipid environment and nanodisc belt size used for our cryoEM structures, and quantified the differences in deuterium uptake between the two samples. We achieved >76% sequence coverage, including at the important pore-lining residues of TM1, with reduced coverage at the periplasmic loops (residues 65–74) and cytoplasmic side of TM2 (residues 75–90), likely due to lipid shielding preventing digestion (Fig. 3h). Our HDX-MS results identified two regions of increased deuterium uptake in DSPC, spanning residues 27–36 and 40–47 (Fig. 3h). The latter region lies immediately upstream of the periplasmic loops, which were previously shown to stabilise and prolong the expanded state dwell time^79^. Residues 27–36 line the pore entrance in TM1, and deprotection in this region agrees with a conformational rearrangement that increases solvent exposure during channel opening. In DMPC nanodiscs (closed state), TM1 residues remain buried within the membrane. In contrast, in DSPC nanodiscs, TM1 tilts to facilitate pore expansion. This motion flattens the TMD and shifts TM1 toward the membrane-solvent interface, and we propose that this increases its susceptibility to deuterium labelling. Notably, no regions showed protection or reduced deuterium accessibility in DSPC, as all other covered regions remained unchanged (Fig. 3h). Our findings align with previous HDX-MS and EPR studies on MtMscL in detergent, which showed the periplasmic loop residues became exposed upon introducing a mutation that stabilises an expanded state^30^.

### Bilayer thickness and interdigitation in EcMscL gating

**Fig. 4:**
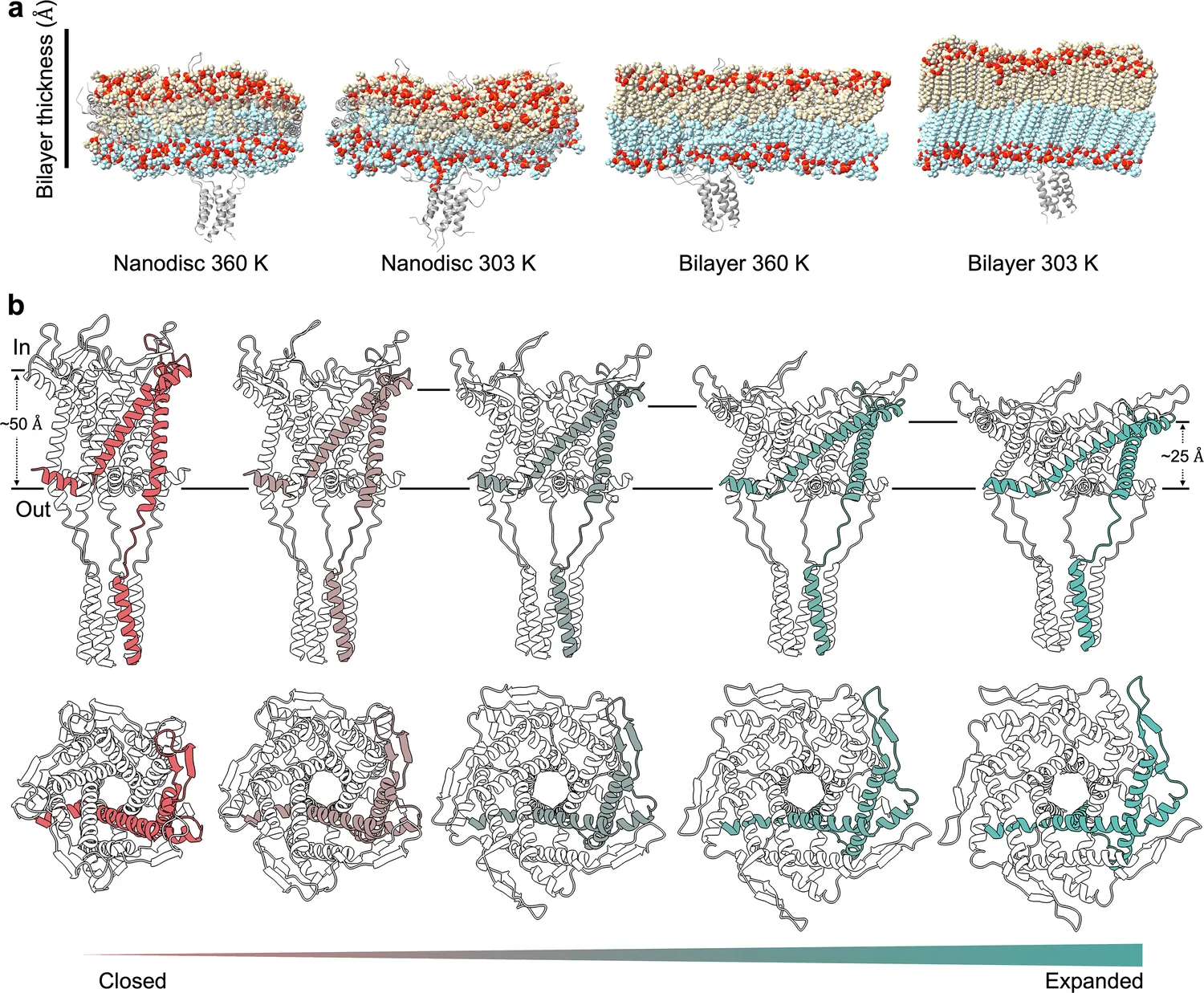
EcMscL bilayers under different temperature and lipid conditions, and corresponding morph of channel expansion. **a** MD simulations of EcMscL embedded in DSPC MSP1E3D1 nanodiscs and planar bilayers at 303 and 360 K. The nanodisc environment promotes local bilayer thinning, resulting in enhanced interdigitation between the acyl chains of opposing lipid leaflets compared to planar bilayers. **b** Structural rearrangements associated with EcMscL channel expansion based on the closed and open-like state(s) structural models, derived by cryoEM.

We next performed atomistic MD simulations of the closed state in DMPC bilayers and the expanded state in DSPC bilayers at 30 °C. To mimic the membrane environment used in the cryoEM experiments, we also performed MD simulations in DMPC MSP1D1 (closed) and DSPC MSP1E3D1 nanodiscs (expanded) at the same temperature. To provide a more complete structural representation, residue sidechains and periplasmic loops, which were not resolved in the expanded-state cryoEM structure, were modelled using the corresponding segments from the AlphaFold-predicted structure^80^. To investigate whether the expanded state is preferentially stabilised in nanodiscs, we analysed bilayer headgroup-to-headgroup thickness^81^ and leaflet interdigitation in planar bilayers^82,83^ and nanodiscs (MSP1D1 and MSP1E3D1). DSPC nanodiscs showed pronounced bilayer thinning and increased interdigitation compared to planar bilayers, particularly at 30 °C, nominally below the *T*_m_ of DSPC. These effects were associated with stabilisation of the expanded/open-like conformation of EcMscL. Although similar behaviour was observed at 87 °C, the effect was marginally weaker. Representative snapshots from the DSPC bilayer and nanodisc simulations are shown in Fig. 4a, and the complete thickness and interdigitation time series are provided in Supplementary Fig. 11c, d.

In contrast, DMPC nanodiscs and native *E. coli* lipid bilayers exhibited only minor changes in bilayer thickness and comparatively low interdigitation, consistent with reduced stabilisation of the expanded state and a preference for the closed conformation of EcMscL (Supplementary Fig. 11a, b). Pore-radius profiles (Supplementary Fig. 12) of all simulated systems reveal larger pore radii for the DSPC nanodisc system(s). Indeed, while all systems undergo compression during the equilibration stage, leading to partial pore closure (compared to the initial models used), the DSPC nanodisc systems maintain a pore radius >2 Å, indicating persistence for a more expanded conformation. This conclusion is corroborated by sustained pore hydration, together with increased membrane thinning and lipid interdigitation observed in DSPC nanodiscs.

### Protein–lipid interactions and critical residues in EcMscL gating

**Fig. 5:**
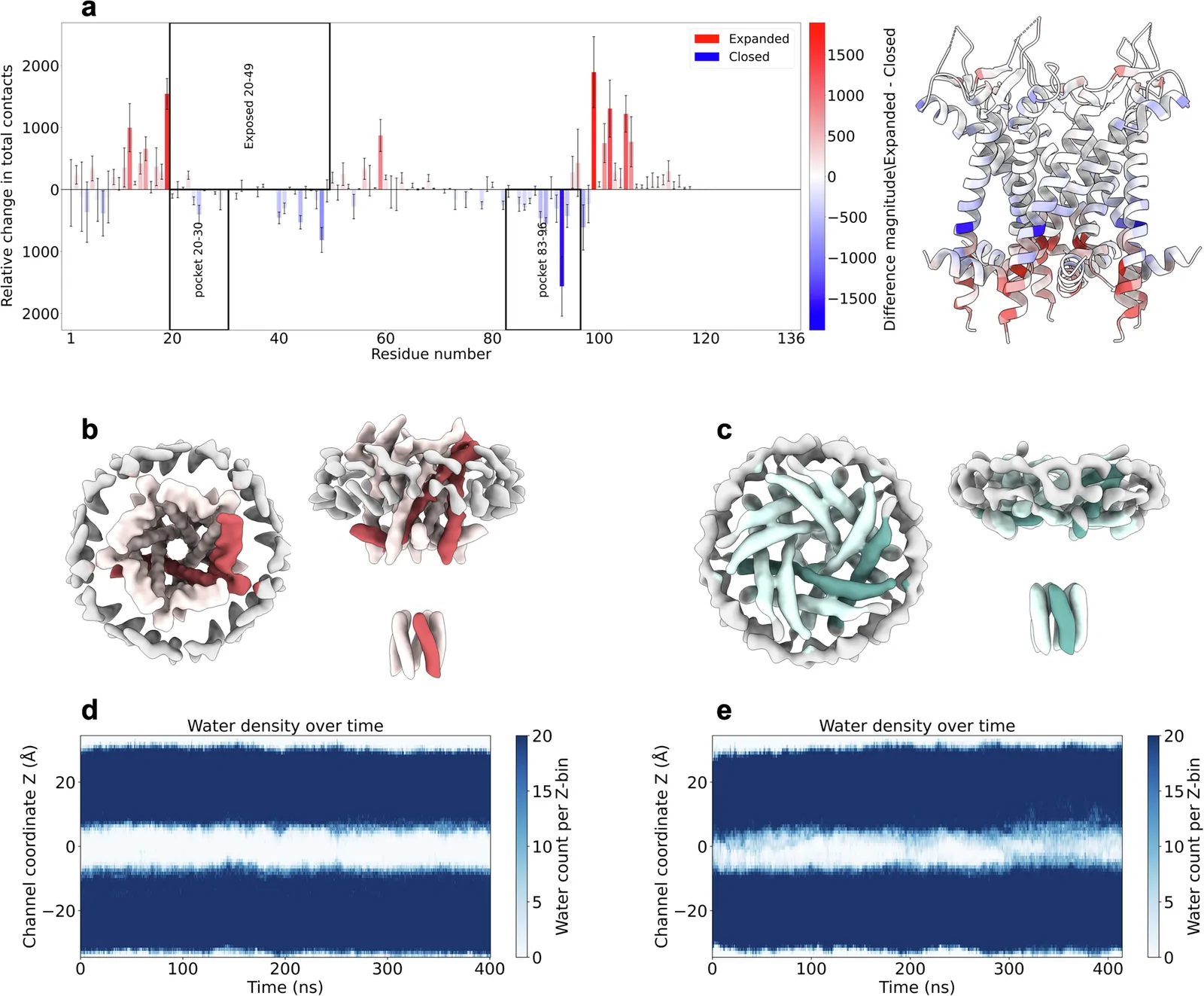
Protein–lipid interactions during EcMscL gating. **a** The net change in protein–lipid contacts between the closed state in DMPC and the expanded state in DSPC is mapped onto the closed EcMscL structure. Inset panels designate the EcMscL pocket regions, for which lipid contacts substantially reduce in the expanded (*vs* closed) state, consistent with the lipid-moves-first model. The coloured bars represent the average over 10 ns and black lines represent the standard deviation across EcMscL subunits. Residues with reduced lipid contacts in the MD during channel expansion showed consistent increased deuterium accessibility in HDX-MS. **b**, **c** Top- and side-view density maps from cryoEM of the closed- and expanded-state EcMscL structures in MSP1D1 DSPC nanodiscs, indicating the MSP-belt protein. **d**, **e** Pore hydration calculations for the closed- and expanded-states in DMPC or DSPC at 30 °C.

We analysed key protein–lipid interactions that stabilise distinct EcMscL conformational states. Following equilibration, the total number of lipid contacts was calculated for each EcMscL residue using a 3 Å radial distance cutoff. The net change in lipid contacts between the expanded and closed states for each residue in nanodiscs is presented as a bar plot and mapped onto the closed-state structure (Fig. 5a). The corresponding analysis for planar bilayers is shown in Supplementary Fig. 13. Several residues, including Ile^3^ and Ile^4^ on S1, Leu^48^, Phe^57^ and Val^59^ on the periplasmic loop, and Leu^98^ on the cytoplasmic side of TM2, interacted with lipids in both conformational states, with Ile^4^, Leu^48^ and Leu^98^ forming the highest number of mean lipid contacts (5–7 contacts per 0.1 ns; Supplementary Fig. 14, while three- to four-fold higher contacts in nanodiscs; Supplementary Fig. 15). Phe^7^, Ile^49^, Leu^61^, Phe^90^, Phe^93^ and Lys^97^ exhibited more contacts in the closed state, whereas Lys^101^, Leu^102^ and Lys^105^ interacted more frequently with lipids in the expanded state (Fig. 5a for nanodiscs and Supplementary Fig. 13 for bilayers). All other residues formed on average fewer than 1 protein–lipid contact per 0.1 ns.

Residues at the periplasmic side of TM1 (20–30), the periplasmic loops (40–49) and the cytoplasmic end of TM2 (83–100) consistently showed a reduction in lipid contacts upon channel expansion (Fig. 5a for nanodiscs and Supplementary Fig. 13 for bilayers), which is in agreement with previous electrophysiology studies^79^. The decrease in lipid contacts at the top of TM1 and the periplasmic loops complements our HDX-MS data, with increased deuterium uptake in the same region in DSPC (Fig. 3h). A reduction in lipid occupancy, or more broadly, a loss of lipid-protein interactions, likely contributes to this enhanced solvent exposure upon channel expansion. In contrast, residues flanking TM2 on both the periplasmic (50–65) and cytoplasmic side (100–105) show a marked increase in lipid contacts during channel expansion. These changes indicate that TM2 undergoes a tilt in which its exposed terminal regions shift closer to the membrane, forming enhanced lipid contacts that likely stabilise the expanded state. This is also consistent with both nanodisc and bilayer MD simulations, where approximately three-fold greater interactions are observed (expanded state) for the initial segment of TM1 (residues 12–19, including pore-forming Leu^19^) in nanodiscs. This indicates a more stable expanded state in nanodiscs than in bilayers.

To evaluate the pore hydration properties of the expanded EcMscL structure in atomistic MD (DMPC and DSPC bilayers) simulations, we performed Channel Annotation Package (CHAP) analysis^84^ (Supplementary Fig. 16). The corresponding nanodisc simulations were analysed by calculating the water density at the pore restriction site. In DMPC and DSPC bilayers, and DMPC nanodiscs, at 30 °C, under equilibrium conditions there are fewer hydration events for the nominally closed state of EcMscL, consistent with a non-hydrated closed channel (Fig. 5d and Supplementary Fig. 16). In DSPC nanodiscs, consistent pore hydration is observed throughout the latter part of the trajectory (300–400 ns) under equilibrium conditions (Fig. 5e). Together, these results indicate that temperature and the associated changes in lipid phase behaviour strongly influence EcMscL hydration in DSPC bilayers and nanodiscs, leading to more frequent yet transient openings at lower temperatures.

## Discussion

EcMscL is the first MS ion channel to be identified and among the best-characterised. Since its discovery^3,5^, its function has been extensively studied through electrophysiology^22,85^, mutagenesis^23,86^, structural biology^8,9,15,16,27^ and MD simulations^24,87^. Most studies have focused on the *E. coli* orthologue, yet its full-length structure, including the TMD critical for gating and function, has remained unresolved. Here, we present EcMscL structures in both closed and expanded states, providing essential insights into the molecular basis of its gating mechanism.

Using PELDOR spectroscopy, we monitored the conformational ensemble of EcMscL in different lipid membranes and nanodisc scaffolds and identified a condition, namely MSP1D1 and DSPC lipids, that shifted the equilibrium of the ensemble away from the closed state, subsequently allowing us to resolve an expanded structure by cryoEM. DSPC shares the same phosphatidylcholine (PC) head group as DMPC and DOPC, and like DOPC, it contains 18-carbon acyl chains; however, the absence of a cis-double bond in DSPC (18:0) results in tighter lipid packing and a high *T*_m_ of ~55 °C^88^ (in comparison, the *T*_m_ values of DMPC (14:0) and DOPC (18:1) are 23 °C^88^ and 17 °C, respectively^89^). At temperatures below the *T*_m_, lipids form a solid-like state known as the gel phase, which is characterised by reduced mobility and slower dynamics^90^. MD simulations indicate that, in high-melting temperature bilayers, lipids exhibit collective motion with neighbouring lipids, but this coordinated behaviour is not observed in the liquid-crystalline phase^91,92^. This effect is due to increased cooperativity caused by interactions between the lipid tails.

During reconstitution, a sufficient number of lipids are displaced from the nanodisc to accommodate the membrane protein. We speculate that reconstitution into high-melting-temperature lipids may cause substantially more lipid depletion from the nanodisc (Fig. 5b, c). As the surface area of the lipid bilayer in nanodiscs is fixed, with the dimensions constrained by the MSP belt, the remaining lipids must occupy a larger area to maintain bilayer integrity and prevent water access, thereby generating membrane tension. This is consistent with our cryoEM structures, where the channel occupies a larger cross-sectional area within the same nanodisc size, suggesting a smaller number of lipids is present in the expanded state, compared to the closed state (Fig. 5b, c).

A similar mechanism for channel expansion was described using CDs, which sequester lipids from the bilayer. CD treatment of reconstituted EcMscS^50–52^ has been shown to induce a conformational shift toward more open-like states; however, in the case of EcMscL^52^ and the MscS-like EcYnal^93^, which both have substantially higher tension activation thresholds compared to EcMscS, high-resolution three-dimensional structure determination of fully open states by cryoEM was not achieved. Similarly, lipid depletion from hydrophobic pockets in MtMscL produces an expanded sub-conducting state^13,30^. According to the lipid-moves-first model for mechanosensing, applicable to EcMscL, lipids behave as weak negative allosteric modulators, stabilising the closed-state channel^32,44^. Although no lipids were resolved in either the closed or expanded state structures, it is possible that upon tension increase, lipids were pulled from the pockets into the bilayer, driving a conformational change to the expanded state, similar to MtMscL^13,30^ and EcMscS^11,94^. Indeed, this is consistent with our MD simulations, where fewer lipids were observed penetrating the pockets in DSPC bilayers (expanded state) compared to DMPC (closed state) (Fig. 5a). Notably, the hydrophobic pockets lining the cytoplasmic side of EcMscL reduce in volume when the channel expands, suggesting they can accommodate fewer lipids in the expanded state (Fig. 5a–c).

A notable difference between EcMscL and MtMscL is the position of the CTD. In EcMscL, the CTD sits ~25 Å below the TMD, whereas in MtMscL, it lies close to the inner membrane leaflet. The ten amino-acid periplasmic loops in EcMscL are proline-rich, likely stabilising a narrow range of CTD orientations and aiding the full-length structure determination. In contrast, the shorter MtMscL loops are more flexible and hydrophilic^8,9^. Structural differences in the CTD may underlie the distinct functional properties of EcMscL and MtMscL. MtMscL requires twice as much tension to open^12,71^, yet its unitary conductance (~2.7 nS) is lower than EcMscL (~3.3 nS)^71^. A C-terminally truncated MtMscL mutant exhibits a similar gating threshold and conductance to wild-type EcMscL^71^, highlighting the CTD’s role in regulating MS channel gating. The CTD appears to constrain the movement of TM2, and shorter cytoplasmic loops appear to enhance this effect.

The CTD has been proposed to act as a molecular sieve, ensuring cells retain crucial metabolites during osmotic downshock^25^. Whilst this may apply to some orthologues like MtMscL, where the TMD and CTD are tightly packed, our EcMscL structures suggest the CTD’s role in filtration is limited. The long cytoplasmic loops create large openings (~25 Å wide) between the domains, and with a pore radius of ~3 Å, the CTD is unlikely to form part of the primary permeation pathway. Our EcMscL structures provide a molecular basis for earlier observations showing that large molecules, antibiotics, and even small proteins can traverse the channel pore^11,73,95,96^. The CTD likely provides structural stability, enabling the protein to retain its oligomeric arrangement during the dramatic close-to-open transition. Moreover, our expanded structure shows that the CTD does not dissociate during channel expansion, contradicting early gating models^19,22^. Whilst CTD dissociation cannot be ruled out for the fully open state, it is unlikely, as it would impose an unnecessary energy cost and may adversely affect the reassembly of the closed-state channel. The considerable tilt of the TMD helices in the expanded state translated to only a moderate 5 Å upward shift in the CTD, and the cytoplasmic helices are of sufficient length to allow the TMD to reach its maximum diameter without the CTD unravelling.

The periplasmic loops in EcMscL are six residues longer than in MtMscL. Both channels feature a short β-hairpin^8^, but EcMscL also has a partial helix formed by Phe^54^, Lys^55^ and Gln^56^, a motif also present in MaMscL^16^. In the expanded MaMscL structure, this loop extends and unravels to facilitate pore opening^16^. Our MD simulations of EcMscL protein–lipid contacts identified residues Phe^57^ and Val^59^, which immediately follow the periplasmic short helix, as important for stabilising the expanded state structure. These findings complement previous functional studies showing that mutations here destabilise the open state, indicating a role in open-state stabilisation. For example, shortening the loops by deleting Gln^56^ increases the tension threshold^86^, and cleaving the loops entirely lowers the activation barrier^97^. Mutating Gln^56^ to an aromatic residue significantly increases the channel’s open dwell time^86^, suggesting the periplasmic helical structure acts as a molecular “spring” to control expansion and retraction at the pore entrance^19^. At the periplasmic end of TM2, residues Lys^101^, Leu^102^ and Lys^105^ were also found to stabilise the open-like state through enhanced lipid contacts. These interactions reflect a shift in the position of TM2 during gating that brings its exposed flanking regions closer to the membrane. Prior work shows that Lys^101^ and Leu^102^ interact with negatively charged lipid head groups^85^, while Lys^105^ is part of a charged cluster important for MscL lipid binding^98^. As a result, these residues located at the boundary between TM2 and the cytoplasmic loops are recruited during gating and play a role in stabilising the open-like state. Conversely, we also identified residues, namely Ile^3^, Leu^4^, Leu^48^ and Leu^98^, with increased lipid contacts in the closed state. The former two are located on the N-terminal S1 amphipathic helix, which is important for conferring mechanosensitivity to the channel^29^. Partial truncations of S1, which remove Ile^3^ and Leu^4^, produce a loss-of-sensitivity phenotype that requires more tension to gate compared to wild-type EcMscL^29^. Taken together, these results indicate that lipid–protein interactions around S1 play a role in maintaining the structural integrity of EcMscL, consistent with previous reports^29^.

DSPC nanodiscs compensate for the bilayer thickness mismatch by locally thinning the bilayer through enhanced interdigitation of the acyl chains, thereby adapting to accommodate the expanded EcMscL conformation. This behaviour is consistently observed at high temperatures (87 °C), but the effect is more prominent at room temperature (30 °C), which is below the melting temperature of DSPC. These findings suggest that the increased interdigitation induced by the nanodisc membrane environment contributes to stabilising the expanded/open-like state of EcMscL.

In summary, by resolving the first EcMscL structure in two distinct conformational states, this study provides insights into its functional properties and gating mechanism. Our structures support the helix-tilt model (Fig. 4b and Supplementary Video 1), previously inferred from computational and spectroscopic analyses. The expanded state, stabilised by altering the lipid composition rather than fusion partners or truncations, highlights the critical role of protein–lipid interactions in modulating the channel’s conformation. Although the precise mechanism by which DSPC lipids promote EcMscL expansion requires further investigation, our findings, informed by multiple experimental and computational approaches, suggest that lipid phase properties influence MS channel conformation in nanodiscs. Generating membrane tension in purified/reconstituted protein samples remains very challenging, and this method constitutes a distinct approach that can be applied to study other MS integral membrane proteins.

## Methods

### Recombinant EcMscL protein expression and purification for cryoEM

A pET-52b vector encoding full-length wild-type EcMscL (UniProt P0A742) with a C-terminal His-tag was transformed into BL21 (DE3) cells for protein expression. Cells were grown in Luria-Bertani (LB) medium with ampicillin (100 µg mL^−1^) at 37 °C, 180 rpm to an OD_600_ of 0.8, then expression was induced by the addition of 1 mM isopropyl-β-D1-thiogalactopyranoside (IPTG) for 4 h at 25 °C, 180 rpm. Cells were harvested by centrifugation, resuspended in 25 ml lysis buffer (50 mM sodium phosphate, 300 mM NaCl, pH 7.5) per litre of cell culture, and lysed using a cell disruptor (Constant Systems) at 30 kpsi. Intact cells and debris were removed by centrifugation (5000 × *g*, 4 °C, 20 min), and the supernatant was centrifuged (125,000 × *g*, 4 °C, 1 h) to pellet the membrane. Membranes were resuspended in 25 mL solubilisation buffer (50 mM sodium phosphate, 300 mM NaCl, 10% (v/v) glycerol, 50 mM imidazole, 1.5% (w/v) DDM, pH 7.5) per gram of membrane, then solubilised for 1 h at 4 °C. The insoluble fraction was removed by centrifugation (125,000 × *g*, 4 °C, 1 h), and the supernatant was incubated with 0.5 mL Ni^2+^-nitrilotriacetic acid (Ni^2+^-NTA) resin per litre of cell culture overnight at 4 °C. The supernatant-resin suspension was poured through a gravity-flow column and washed with 25 column volumes (CV) wash buffer (50 mM sodium phosphate, 300 mM NaCl, 10% (v/v) glycerol, 50 mM imidazole, 0.05% DDM, pH 7.5), and protein was eluted in 1 mL fractions in wash buffer supplemented with 300 mM imidazole. The eluates were pooled and applied onto a Superdex 200 Increase 10/300 column (Cytiva) equilibrated with SEC buffer (50 mM sodium phosphate, 300 mM NaCl, 0.05% (w/v) DDM, pH 7.5). The purified protein was snap-frozen in liquid nitrogen and stored at -80 °C.

### Purification and spin-labelling of EcMscL for EPR spectroscopy

The expression and purification of EcMscL for EPR spectroscopy were identical to that for cryoEM, with the exception that the protein was spin-labelled with the nitroxide spin label MTSSL before eluting from the gravity-flow column. Following the column wash, an additional wash step was performed using 5 CV of wash buffer supplemented with 3 mM Tris (2-carboxyethyl) phosphine hydrochloride (TCEP) to reduce disulphide bonds between cysteines. The TCEP was subsequently removed using 10 CV of wash buffer. For on-column spin-labelling, a 100 mM stock solution of MTSSL was prepared in 100% (v/v) dimethyl sulfoxide (DMSO), then diluted in wash buffer to a final concentration of 1.5 mM. The EcMscL-bound Ni^2+^-NTA resin was incubated with 2 CV wash buffer containing MTSSL for 20 min at room temperature, and subsequently at 4 °C overnight. Free label was removed with 10 CV wash buffer, then the protein was eluted in 1 mL fractions in wash buffer supplemented with 300 mM imidazole.

### Quantification of EcMscL spin-labelling efficiency

Quantification of EcMscL spin-labelling efficiency by CW-EPR is challenging, because robust determination of protein concentration is not highly accurate. This arises due to a low molar extinction coefficient (ε=1490 M^−1^ cm^−1^), preventing accurate concentration determination. We therefore use modulation depths from PELDOR experiments as an indirect measure to constrain both labelling efficiency and oligomeric distribution. EcMscL exists in equilibrium between monomeric and pentameric states. The observed modulation depth (Δ) can then be described as a weighted mixture by eq. (1)^99,100^:

1 $$\Delta=1-\frac{{\sum }_{k=1}^{n}\left(\begin{array}{c}n\\ k\end{array}\right){f}^{k}{\left(1-f\right)}^{n-k}{\left(1-\lambda \right)}^{k-1}k\,x\left(k\right)}{{\sum }_{k=1}^{n}\left(\begin{array}{c}n\\ k\end{array}\right){f}^{k}{\left(1-f\right)}^{n-k}k\,x\left(k\right)}$$

Where: n is the total number of labelling sites (i.e., here, each monomer contains a single cysteine residue), k is the number of distinct states containing k spins, f is the labelling efficiency, λ is the inversion efficiency at the pump frequency, and $x\left(k\right)$ is the fractional population of oligomeric species containing k spins. For nitroxide spin labels at Q-band frequencies and using rectangular microwave pulses, λ is typically 0.25–0.35^99,101^. Therefore, we use the conservative value of 0.25 and note that while a full calibration is most reliable to calculate λ, this is beyond the scope of this study. For a fully labelled pentamer (n=5), the maximum observable modulation depth (${\Delta }_{\max }$) is defined as:

2 $${\Delta }_{\max }\,=1-{\left(1-\lambda \right)}^{n-1}$$

This defines the upper physical limit for Δ under experimental conditions, assuming quantitative labelling efficiency (f= 1) and pentamer fraction (${{{{\rm{x}}}}}_{5}=$1). The SEC profiles in DDM detergent (prior to reconstitution) indicated two peaks (Supplementary Fig. 17b), likely corresponding to a pentamer-enriched fraction (peak 1) and a monomer-enriched fraction (peak 2) for both I79R1 and M94R1. Though it is understood that the profile is not quantitatively symmetric, we assume that the pentamer and monomer fractions are roughly inverted across peaks 1 and 2. We measured PELDOR and observed ${\Delta }_{{{{\rm{peak}}}}1}$ of 0.496 and 0.476 for I79R1, in detergent and after reconstitution into DSPC MSP1D1 nanodiscs, respectively. Similarly, we observed ${\Delta }_{{{{\rm{peak}}}}1}$ of 0.562 and 0.563 for M94R1, in both detergent and after reconstitution into DSPC MSP1D1 nanodiscs, respectively. We estimated a modulation depth error of 2-4%, corresponding to ±0.01–0.02, consistent with previous literature^102–104^. Global fitting and subsequent bootstrapping (over 500 trials) of the experimental modulation depths to estimate parameters ${{{{\rm{x}}}}}_{5}$, ${{{{\rm{f}}}}}_{{{{\rm{I}}}}79{{{\rm{R}}}}1}$, and ${{{{\rm{f}}}}}_{{{{\rm{M}}}}94{{{\rm{R}}}}1}$ yielded 2σ confidence intervals: ${{{{\rm{x}}}}}_{5}=$ 0.794$\,\pm$ 0.020, ${{{{\rm{f}}}}}_{{{{\rm{I}}}}79{{{\rm{R}}}}1}=$ 0.645$\,\pm$ 0.024, and ${{{{\rm{f}}}}}_{{{{\rm{M}}}}94{{{\rm{R}}}}1}=$ 0.797$\,\pm$ 0.024. As an independent validation, we subsequently measured PELDOR of I79R1 and observed ${\Delta }_{{{{\rm{peak}}}}2}$ of 0.285 (Supplementary Fig. 17a), in excellent agreement with the predicted value of 0.288 (assuming ${x}_{5}=$ 0.206, ${{{{\rm{f}}}}}_{{{{\rm{I}}}}79{{{\rm{R}}}}1}=$ 0.645, and λ= 0.25). The scripts used for this analysis will be submitted in the underpinning data.

We note that the reconstitution efficiency in MSP1E3D1 nanodiscs was lower and demonstrated greater variability than for MSP1D1 nanodiscs in DSPC lipids—modulation depths were 0.301 and 0.548 for I79R1 biological replicates, and 0.371 for M94R1, respectively; thus, we exclude the MSP1E3D1 data from the fit.

### Reconstitution of EcMscL into lipid nanodiscs

Purified EcMscL was reconstituted into nanodiscs composed of MSP1D1 or MSP1E3D1^105^ and DMPC, DOPC or DSPC lipids (Anatrace). The His-tag on MSP1D1 was removed by incubating purified MSP1D1 with TEV protease at a 1:50 molar ratio (TEV:MSP) overnight at 4 °C. For the reconstitution, DMPC, DOPC or DSPC were dissolved in lipid buffer (50 mM sodium phosphate, 300 mM NaCl, 20 mM sodium cholate, 0.05% (v/v) DDM, pH 7.5), then added to the purified EcMscL and MSP1D1 at a 1:1:80 (EcMscL:MSP:lipids) molar ratio and incubated at room temperature for 40 min. The reconstitution assembly mixture was incubated with Bio-Beads SM-2 resin (Bio-Rad) overnight at 4 °C, then added to Ni^2+^-NTA resin for 2 h at 4 °C. The assembly mixture was poured through a gravity-flow column, washed with 20 CV wash buffer (50 mM sodium phosphate, 300 mM NaCl, 40 mM imidazole, pH 7.5), then eluted in wash buffer supplemented with 250 mM imidazole. Imidazole was removed from the sample by exchanging the protein into an imidazole-free wash buffer during concentration. The SDS–PAGE gels and associated size-exclusion chromatogram after reconstitution are shown in Supplementary Fig. 18.

### EPR sample preparation

EPR samples were prepared to a final volume of 65 µL to ensure complete filling of the active volume of the resonator cavity. To ensure formation of a glassy (i.e., homogeneously distributed) frozen solution, 35% (v/v) ethylene glycol d-6 (Deutero GmbH) was added and thoroughly mixed by pipetting. Samples were transferred by gas-tight syringe (Hamilton) and PTFE tubing to quartz EPR tubes (o.d. 3 mm; Wilmad). All EPR samples were immediately frozen by direct immersion into liquid nitrogen.

### EPR instrumentation and measurement parameters

All pulse EPR measurements were performed using a Bruker ELEXSYS 580 pulse EPR spectrometer, and temperatures were maintained using a cryogen-free variable temperature cryostat (Cryogenics Ltd). All two-pulse electron spin-echo (ESE) measurements of the electron spin transverse dephasing time (${T}_{m}$) and all four-pulse dead-time-free PELDOR^106,107^ measurements were performed at 50 K, using a 300 W travelling wave tube (TWT; Applied Systems Engineering) at Q-band frequency (34 GHz) in a 3 mm cylindrical resonator (Bruker ER 5106QT-2w in TE012 mode), in over-coupled mode. All CW-EPR spectra were recorded using a Bruker Magnettech ESR5000 spectrometer operating at X-band (9.4 GHz) and at 298 K, before the addition of cryoprotectant.

The CW-EPR measurements were performed using a sweep time of 60 s, over 60,000 points, with a sweep width of 20 mT (magnetic field values between 325 and 345 mT), 10 dB high-power attenuation (2 mW power), a modulation amplitude of 0.1 mT, a modulation frequency of 100 kHz, and a variable number of scans to reach sufficient signal-to-noise ratio (SNR).

The 4-pulse PELDOR experiment^106^ (π/2(${{{{\rm{\nu }}}}}_{{{{\rm{A}}}}}$)-${{{{\rm{\tau }}}}}_{1}$-π(${{{{\rm{\nu }}}}}_{{{{\rm{A}}}}}$)-(${{{{\rm{\tau }}}}}_{1}$+t)-π(${{{{\rm{\nu }}}}}_{{{{\rm{B}}}}}$)-(${{{{\rm{\tau }}}}}_{2}$-t)-π(${{{{\rm{\nu }}}}}_{{{{\rm{A}}}}}$)-${{{{\rm{\tau }}}}}_{2}$-echo) was used, where ${{{{\rm{\nu }}}}}_{{{{\rm{A}}}}}$ and ${{{{\rm{\nu }}}}}_{{{{\rm{B}}}}}$ indicate the pulse excitation at the observer and pump frequencies, respectively. For lambda-reduced PELDOR measurements, a nominal inversion efficiency (${{{{\rm{\lambda }}}}}_{0}$) of 0.25 was assumed from literature for rectangular microwave pulses at Q-band frequency^99^. In all cases, pulses of lengths 16 and 32 and 16–32 ns were used for observer and pump pulses (*π*/2, *π* and *π*), respectively. The magnetic field and microwave frequency were adjusted for the maximum of the nitroxide spectrum to coincide with the pump pulse position, while the observer pulse was placed at +80 MHz frequency offset (pump-detection frequency). All EcMscL PELDOR measurements used a ${{{{\rm{\tau }}}}}_{1}$ of 380 ns, time window (${{{{\rm{\tau }}}}}_{2}$) of 3000–3500 ns, a dipolar increment of 12 ns, 265–348 points, 10 shots-per-point, a shot repetition time (SRT) of 2000 μs, and acquisition times of 16-48 hours. Unwanted echoes were suppressed using a 16-step phase cycle, and electron spin-echo envelope modulation (ESEEM) couplings arising from deuterium nuclei were suppressed using an 8-step tau-averaging cycle^108^ with a 16 ns increment. All samples, except for the M94R1 MSP1E3D1 condition in DSPC, were measured as technical repeats (n=2). Additionally, biological replicates (n=2) of I79R1 and M94R1 MSP1D1 conditions in DSPC yielded similar results.

### PELDOR data processing, analysis, and in silico modelling

For each experimental condition, technical replicate (n=2) PELDOR data were fitted globally inside DeerLab^109^, using compactness regularisation^110^ (Fig. 2; Supplementary Fig. 5). The regularisation parameter (α) was optimised using the general cross-validation (GCV) functional^111^. For cross-validation of the output distance distributions, analyses were also replicated, with automated data processing performed inside DeerAnalysis2022^112^, using the ComparativeDeerAnalyzer (CDA) 2.0^109,113,114^ (Supplementary Fig. 5). For PELDOR measurements performed without lambda-reduction, power-scaling assuming 5 spins per MscL pentamer was applied^115,116^ (when processed using the CDA 2.0). DeerLab global fitting was performed without post-processing methods to suppress multi-spin effects, owing to removal of negative time-points during CDA processing, obfuscating determination of the zero-time. PDF outputs from CDA 2.0 are supplied in the underpinning data.

All in silico modelling of distance distributions was performed using the closed and expanded structures determined in this study (PDB closed: 28OA; PDB expanded: 28OC), implemented in the mtsslSuite webserver^117^, using an accessible volume approximation to calculate spin label rotamers^118^, and allowing the maximum number of steric clashes (i.e., “very loose” setting). For calculation of the local (Bhattacharyya coefficient; BC) and global (first Wasserstein distance; W_1_) similarity metrics, distance distributions were first linearly interpolated onto a shared distance axis over 600 points, with outlier values set to zero. Next, BCs were calculated for each experimental distance distribution compared to either the closed- or expanded-state in silico distance distribution as previously described^119,120^ using eq. (3):

3 $${{{\rm{BC}}}}={\sum}_{n\in N}\sqrt{P(n)\times Q(n)}$$

Where: P(n) and Q(n) are normalised probability distributions over the same domain N. Conversion from probability density distributions to probability distributions is performed using eq. (4):

4 $$P\left(n\right)=\,\frac{P(n)}{{\sum }_{n\in N}P(n)}$$

Finally, ${W}_{1}$ values were calculated in discretized form^121,122^ using eq. (5):

5 $${W}_{1}\approx {\sum}_{i=1}^{N}\left|{F}_{\exp }\left({r}_{i}\right)-{F}_{{sim}}\left({r}_{i}\right)\right|\Delta r$$

Where: Δr is the discrete interval over the domain N, while ${F}_{\exp }$ and ${F}_{{sim}}$ are cumulative distribution functions corresponding to the experimental distance distribution and the closed- or expanded-state in silico distance distribution, respectively, defined using eq. (6):

6 $$F\left({r}_{i}\right)={\sum}_{j=1}^{i}P({r}_{j})\Delta r$$

Where: $P({r}_{j})$ is the probability density function evaluated at the discrete grid-point ${r}_{j}$ on the discrete axis with step ∆r. The MATLAB scripts used for this analysis are available from the authors upon request.

### CryoEM data acquisition

Reconstituted EcMscL was concentrated to 0.8–1.1 mg mL^−1^ ( ~ 50 µM) and centrifuged (20,000 × *g*, 4 °C, 10 min) to remove protein aggregates before preparing grids. 3 µL sample was applied to glow-discharged (12 mA, 45 sec) Quantifoil R2/2 or UltrAUfoil R2/2 300 mesh grids, then plunged into liquid ethane using a Vitrobot Mark IV (4 °C, 100% humidity, blot force 6, blot time 6). Grids were transferred to and stored in liquid nitrogen. Data were collected on an FEI Titan Krios with a Falcon 4i detector. For the DOPC dataset, the detector was equipped with a Selectris energy filter operated with a 10 eV slit width. For the DMPC condition, two independent datasets were collected using identical parameters and combined during image processing. Supplementary Table 5 summarises the microscope parameters and model refinement statistics for all datasets.

### CryoEM image processing

#### EcMscL MSP1D1/DMPC nanodiscs

The raw data from one collection comprising 4177 movies were imported into RELION 4.0^123,124^ for motion correction and CTF estimation using MotionCor2^125^ and CTFFIND4^126^. Particles were picked using the reference-free Laplacian of Gaussian algorithm in RELION 4.0, then extracted with binning and imported into CryoSPARC^127^. Two rounds of 2D classification were performed before three initial models were generated by ab-initio reconstruction. A template for picking was generated from the best model. The raw data from two separate collections comprising 13,105 movies were imported into CryoSPARC and pre-processed using Patch Motion Correction and Patch CTF. Micrographs with an estimated CTF fit resolution greater than 5 Å were rejected. 7,229,245 particles were extracted at a box size of 336 Å, binned by 2, following template picking and inspection. After one round of 2D classification, 16% of the particles were selected to generate five initial models by ab-initio reconstruction without symmetry, followed by heterogeneous refinement. The particles in the best class (439,098) were reextracted at 324 Å without binning and then subjected to another round of 2D classification. Ab initio reconstruction was carried out on the re-extracted particles to assess conformational heterogeneity in the sample. Non-uniform refinement with C_5_ symmetry was performed on the best class (121,887 particles) to yield a 3D reconstruction at 4.08 Å resolution. The density map was subsequently sharpened by DeepEMhancer to improve the quality and interpretability^128^.

#### EcMscL in MSP1D1/DOPC nanodiscs

A total of 7095 movies were imported into RELION 4.0^123,124^ for motion correction and CTF estimation using MotionCor2^125^ and CTFFIND4^126^. Particles were picked using the general model in crYOLO^129^ with a threshold of 1.5, resulting in 868,750 particles, which were extracted at a box size of 300 Å, with binning. The particles were imported into CryoSPARC^127^ and subjected to one round of 2D classification. A subset comprising 29% of the particles was used to generate six initial models using ab-initio reconstruction without symmetry, followed by two rounds of heterogeneous refinement. Particles from the best class (33%) were further assessed by 2D classification and subsequently re-extracted at 352 Å without binning, before running ab-initio reconstruction with three classes. The best class (78%; 52,307 particles) was refined using non-uniform refinement. A mask encompassing only the TMD and CTD, while excluding the nanodisc density, was generated in UCSF ChimeraX^130^, and imported into CryoSPARC with a soft padding of 40 pixels. Local refinement using the non-uniform model and this mask yielded a final 3D reconstruction at 4.50 Å resolution.

#### EcMscL in MSP1D1/DSPC nanodiscs

A total of 20,195 movies were motion corrected in RELION 4.0^123,124^, then imported into CryoSPARC for Patch CTF estimation^127^. Micrographs with an estimated CTF fit resolution >5 Å were rejected. 4,746,208 particles were picked using a template-free blob picker, then extracted at a box size of 300 Å with binning. Three rounds of 2D classification were performed to remove bad particles and empty nanodiscs, then 118,115 particles were used to generate five initial models by ab-initio reconstruction without symmetry, followed by heterogeneous refinement. Particles in the two best classes (43,828 particles) were extracted at 300 Å without binning, then ab-initio was used to separate the particles into three classes. The particles and initial model in the best class (17,211 particles) were refined using non-uniform refinement to yield a 3D reconstruction at 6.95 Å resolution.

### Model building and refinement

To build a model of EcMscL, a chimeric model featuring the TMD of EcMscL from the AlphaFold 3^80^ predicted structure (residues 1–107) and the CTD of EcMscL from the published crystal structure^7^ (residues 108–136) was generated in UCSF ChimeraX^130^. The model was rigid-body fit into the cryoEM density map of EcMscL in MSP1D1/DMPC nanodiscs using Coot 0.9^131^, then refined using Coot 0.9 and ISOLDE v1.6^132^. Due to insufficient density at the periplasmic loops, some residues were removed from the models (residues 64–69 in DMPC; 63–70 in DOPC; and 50-74 in DSPC). Refinement was performed in cycles and validated using Phenix v1.17.1-3660^133^. The model validation statistics calculated by MolProbity^134^ for the closed state conformation in DMPC or DOPC lipids, and the expanded state in DSPC lipids, are listed in Supplementary Table 5.

### Hydrogen-deuterium exchange mass spectrometry

Reconstituted EcMscL was diluted to 15 µM in equilibration buffer (50 mM sodium phosphate, 150 mM NaCl, pH 7.5), then 5 µL of sample was added to 95 µL of deuterated buffer (50 mM sodium phosphate, 150 mM NaCl, pD 7.5) and incubated at 4 °C for 0, 0.5, 5 or 30 min. Deuterium exchange was quenched by combining 75 µL of the labelling reaction with 75 µL of a low-pH buffer (50 mM sodium phosphate, 0.2% (v/v) DDM, pH 1.8). 50 µL of the quenched sample was passed through an Enzymate Protein Pepsin Column (Waters) (40 µL min^−1^, 20 °C) and a VanGuard Pre-column Acquity UPLC BEH C18 (1.7 µm, 21 mm × 5 mm, Waters). The peptides were transferred to a C18 column (75 µm × 150 mm, Waters) and separated by gradient elution of 0 − 40% (v/v) MeCN (0.1% (v/v) formic acid) in H_2_O (0.3% (v/v) formic acid). Trapping and gradient elution of peptides were performed at 0 °C. The HDX system was interfaced with a Synapt G2Si mass spectrometer (Waters) operating in HDMS^E^ mode. HDX data were analysed using PLGS (v3.0.2) and DynamX (v3.0.0) software. Restrictions for identified peptides in DynamX were as follows: minimum intensity—1000, minimum products per MS/MS spectrum—3, minimum products per amino acid—0.3, maximum sequence length—25, maximum ppm error—20, file threshold—4/6. Summary plots were generated using Deuteros 2.0^135^. The mass spectrometry proteomics data have been deposited to the ProteomeXchange Consortium via the PRIDE^136^ partner repository with the dataset identifier PXD077332. A reporting summary based on community guidelines^137^ can be found in Supplementary Table 6.

### Atomistic MD simulations

CHARMM-GUI’s membrane builder tool^57^ was used to build the nanodisc and bilayer systems containing the closed and expanded states of EcMscL. Supplementary Table 7 summarises the environment, composition and simulation details for each system. *E. coli* lipid compositions consisting of (POPE 67%, POPG 23%, and cardiolipin 10%) were used. The systems were solvated using the TIP3 water model and 0.15 M NaCl or KCl. The systems were initialised from NAMD input files and equilibrated in six steps over 1125 ps before starting the production runs. The first two equilibration steps were performed in the NVT ensemble, while the NPT ensemble was used for the remainder of the simulation, including the production. The systems were built with a default pH setup of 7.0 and a constant pressure of 1 atm. Nosé–Hoover Langevin piston^138^ was used for pressure coupling in all simulations, while Langevin thermostat^139^ was used for temperature coupling. Semi-isotropic pressure coupling was employed for planar lipid bilayer simulations, whereas isotropic pressure coupling was used for nanodisc systems. The expanded-state systems were built using DSPC lipids in MSP1E3D1 nanodiscs, while the closed-state systems were built using DMPC lipids in MSP1D1 nanodiscs. NAMD 3.0^140^ was used to equilibrate and produce the simulations, with a default timestep of 2 fs in the CHARMM36 force field^141–143^. Constraints were used for the protein backbone, lipid and water during equilibration, which were gradually scaled to zero in the final equilibration step. In the repeat simulation, we introduced additional harmonic positional restraints for residues 110–136 in the production run, monitoring the fluctuating and highly dynamic EcMscL CTD, but observed no significant effect on the dynamics of the TMD. Simulations were replicated (*n* ≥ 2) for each system, and trajectories were visualised using VMD^144^.

Pore hydration for each lipid bilayer trajectory was computed using CHAP singularity version 2^84^, dependent on GROMACS 2018.8^145^. For nanodiscs, the hydration was calculated using an in-house developed Python code by defining the pore restriction site and filter. Lipid-protein contact analysis was performed as previously described^146^, but included full lipid molecules (i.e., the lipid head group and tails) for the calculation. Contacts were considered when any lipid atoms were within 3 Å of protein atoms for at least 10 ns. Lipid–protein contacts were calculated every 0.1 ns throughout the production trajectories for each MscL monomer. Time-averaged contact frequencies were calculated for each monomer, and the reported values represent the mean ± standard deviation across all monomers. The resulting outputs were analysed and plotted using custom Python scripts developed in-house. Lipid interdigitation was calculated using the correlation fraction metric implemented in MEMBPLUGIN in VMD, based on the overlap of opposing leaflet density distributions along the bilayer normal^147^.

### Statistics and reproducibility

No statistical method was used to predetermine sample size. No data were excluded from the analyses. The experiments were not randomised. The Investigators were not blinded to allocation during experiments and outcome assessment.

### Reporting summary

Further information on research design is available in the Nature Portfolio Reporting Summary linked to this article.

## Supplementary information

Supplementary Information

Transparent Peer Review file

Reporting Summary

Description of Additional supplementary files

Supplementary Video 1

## Source data

Source Data File

## Data Availability

The cryoEM structures of closed-state EcMscL resolved in nanodiscs with DMPC or DOPC lipids are deposited in the Protein Data Bank (PDB) and Electron Microscopy Data Bank under the accession codes 28OA and EMD-56663 and 28OB and EMD-53335, respectively. The expanded state resolved in DSPC lipids can be accessed using 28OC and EMD-56666. The mass spectrometry proteomics data have been deposited to the ProteomeXchange Consortium via the PRIDE^136^ partner repository with the dataset identifier PXD077332. The EPR source data underlying Fig. 2b, c and Supplementary Figs. 5–8 and 17 can be found in the Source Data File. MD source data underlying Fig. 5 and Supplementary Figs. 13–15 are provided on Figshare [https://doi.org/10.6084/m9.figshare.31306639] Source data are provided with this paper.
