# Supplementary material for: Structural basis of the gating mechanism of the large-conductance mechanosensitive channel from Escherichia coli
Source: Nat Commun. 2026 Aug 22;17:10777. doi: 10.1038/s41467-026-76193-0 (PMC13661626; doi:10.1038/s41467-026-76193-0)
Supplement: Supplementary file 1 [file 41467_2026_76193_MOESM1_ESM.pdf]

Supplementary Information for

Structural basis of the gating mechanism of the large-conductance mechanosensitive channel from *Escherichia coli*

Katie Hardman[1,2^], Joshua L. Wort[5,6^], Qaiser Waheed[5,6^], Xinyu Liu[5,6], Diya Arul[5,6], Sebastian A. Porav[1,4], Antonio N. Calabrese[1,3], Stephen P. Muench[1,2], and Christos Pliotas[1,2,5,6*]

[1] Astbury Centre of Structural Molecular Biology, University of Leeds, Leeds LS2 9JT, UK
[2] School of Biomedical Sciences, Faculty of Biological Sciences, University of Leeds, Leeds LS2 9JT
[3] School of Molecular and Cellular Biology, Faculty of Biological Sciences, University of Leeds, Leeds LS2 9JT, UK
[4] Leeds Institute of Cardiovascular and Metabolic Medicine, LIGHT Laboratories, University of Leeds, Leeds LS2 9JT, UK
[5] BioEmPiRe Centre for Structural Biological EPR Spectroscopy, School of Biological Sciences, Faculty of Biology, Medicine and Health, The University of Manchester, Manchester M13 9PT, UK
[6] Manchester Institute of Biotechnology, The University of Manchester, Manchester M1 7DN, UK
^Joint first author contribution
*Corresponding author. Email: christos.pliotas@manchester.ac.uk

The file includes:

Supplementary Figures: 1 to 18

Supplementary Tables: 1 to 7

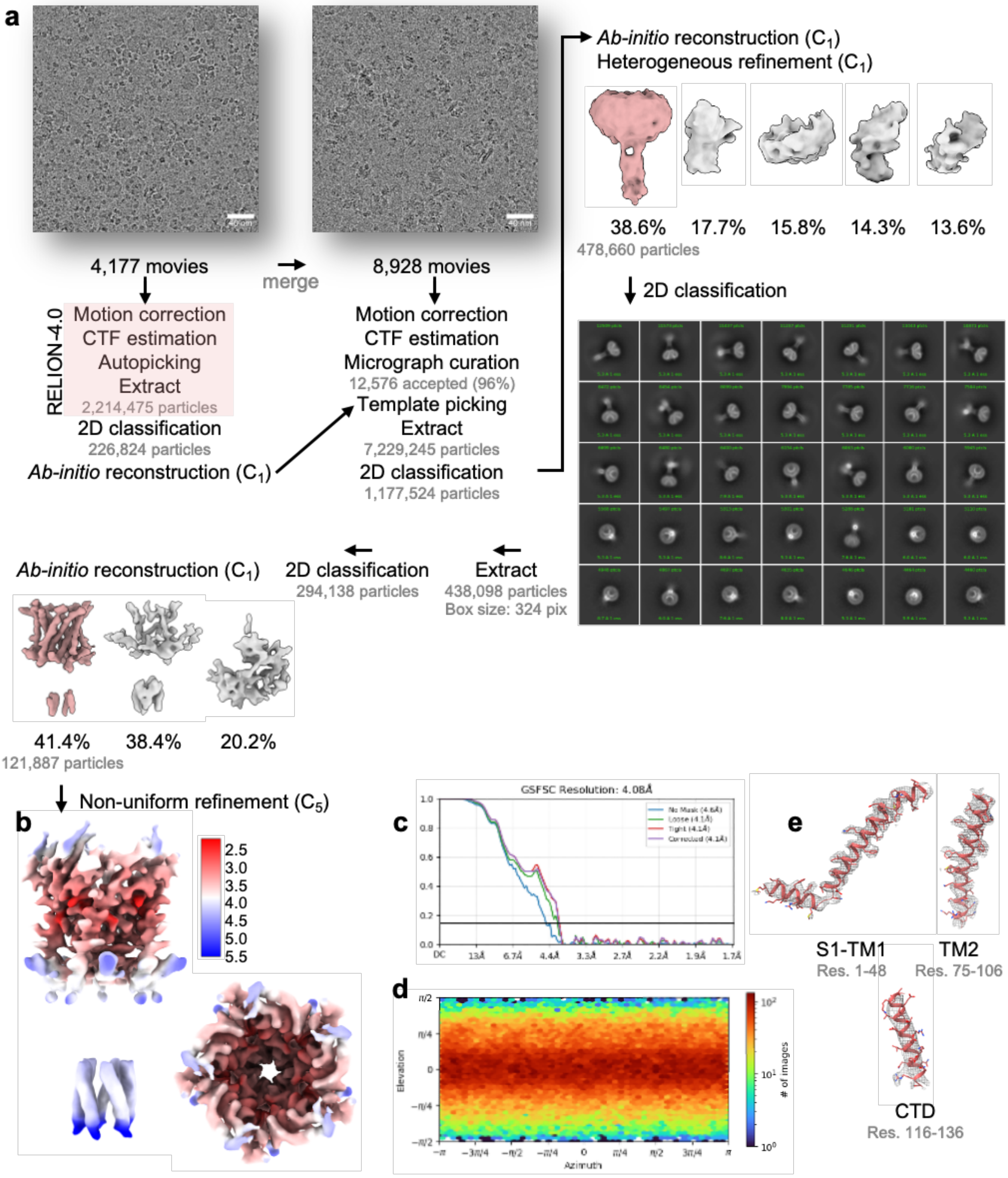

Supplementary Fig. 1: The cryoEM processing pipeline for EcMscL in MSP1D1/DMPC nanodiscs (PDB 28OA). (a) An initial dataset of 4,177 movies was processed in RELION 4.0, yielding 2.21 million particles that were classified in CryoSPARC to generate a template for particle picking. An additional dataset (8,929 movies) was collected and merged with the initial dataset and processed in CryoSPARC, yielding 7.23 million particles after CTF filtering and template picking. Following 2D classification, *ab initio* reconstruction and heterogeneous refinement, the best class (493,098 particles) was re-extracted and further classified. Non-uniform refinement with C_5 symmetry of the final subset (121,887 particles) produced a 4.08 Å reconstruction. (b) The 4.08 Å map coloured by local resolution. (c) Gold-standard FCS curve. (d) Angular distribution of particles in the final reconstruction. (e) Structural

model of EcMscL helices S1 (residues 1-15), TM1 (residues 16-48), TM2 (residues 75-106) and CTD (residues 116-136) in the density map contoured to σ8, shown in ChimeraX.

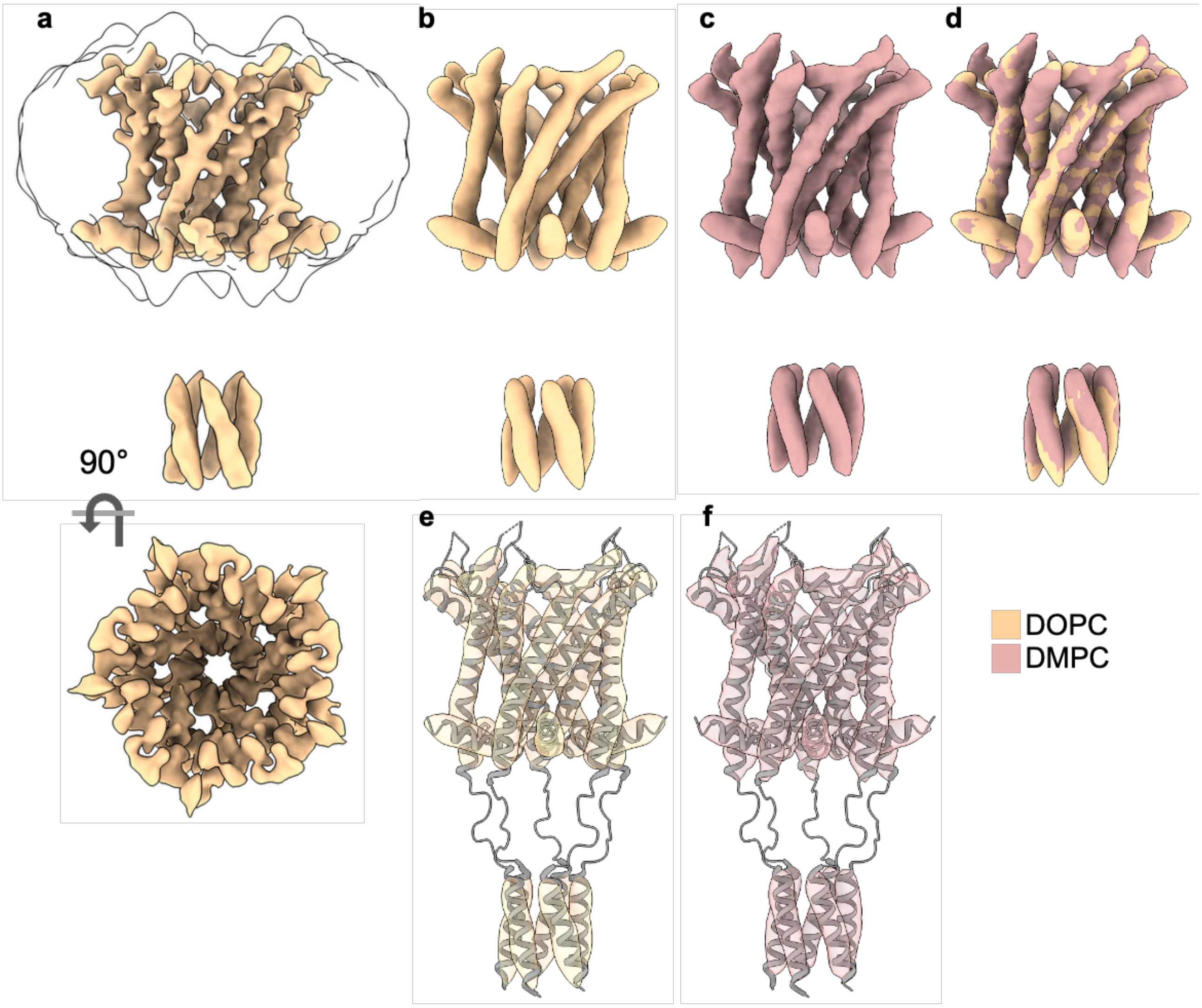

Supplementary Fig. 2. DOPC and DMPC lipid nanodiscs stabilise the closed-state conformation of EcMscL. (a) The 4.50 Å resolution density map of EcMscL in MSP1D1/DOPC nanodiscs shown from side-on (top) and top-down (bottom) views. (b) The unsharpened map of EcMscL in DOPC nanodiscs. (c) The unsharpened map of EcMscL in MSP1D1/DMPC nanodiscs. (d) Comparison between EcMscL in DOPC and DMPC nanodiscs. (e-f) The model of the closed-state EcMscL fits into the density maps from DOPC (orange) and DMPC (pink) conditions.

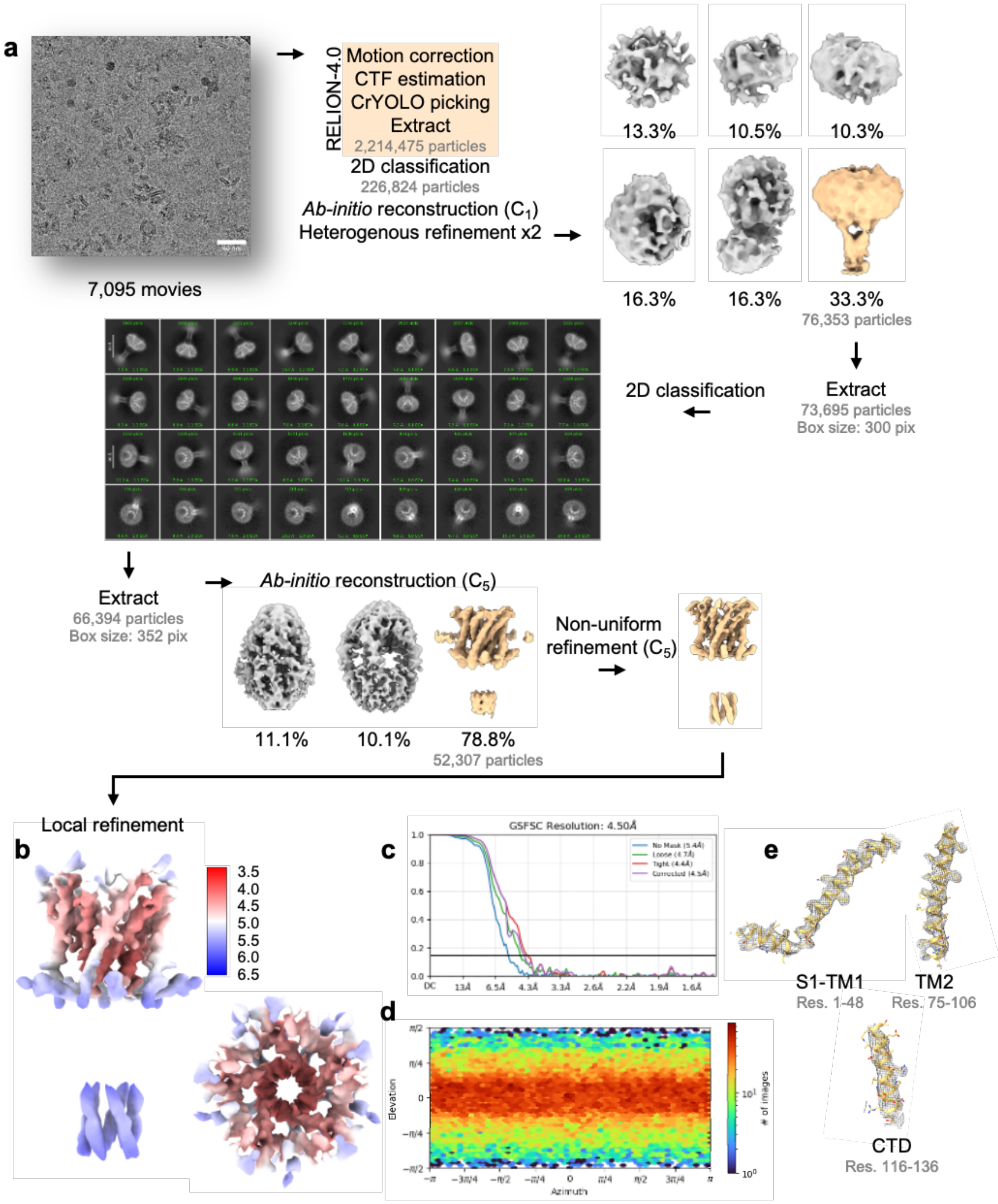

Supplementary Fig. 3: The CryoEM processing pipeline for EcMscL in MSP1D1/DOPC nanodiscs (PDB 28OB). (a) A total of 7,095 movies were processed in RELION 4.0 for motion correction and CTF estimation, yielding 868,750 particles after crYOLO picking. Following 2D classification, *ab initio* reconstruction and heterogeneous refinement in CryoSPARC, particles from the best class were re-extracted without binning and subjected to further classification. The final subset (52,307 particles) was refined by non-uniform refinement and masked local refinement excluding nanodisc density, producing a 4.50 Å reconstruction. (b) The 4.50 Å map coloured by local resolution. (c) Gold-standard FCS curve. (d) Angular distribution of particles in the final reconstruction. (e) Structural model of EcMscL helices S1 (residues 1-15), TM1 (residues 16-48), TM2 (residues 75-106) and CTD (residues 116-136) in the density map contoured to σ8, shown in ChimeraX.

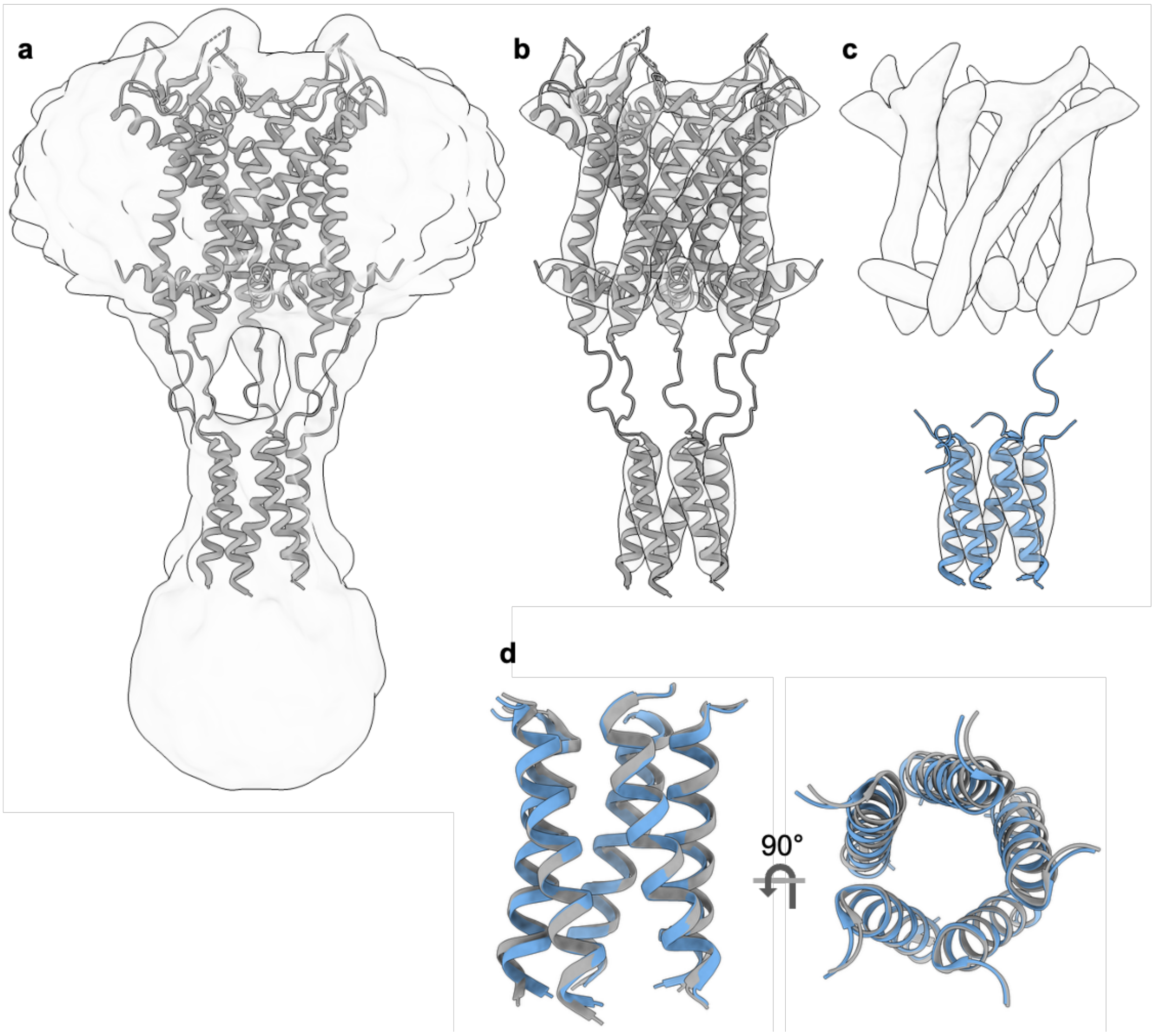

Supplementary Fig. 4: The cytoplasmic loops between the EcMscL TMD and CTD are resolved at a high threshold. (a) The density map of EcMscL in MSP1D1/DMPC allows modelling of loops between domains. (b) The cytoplasmic loop signal is poor at lower thresholds, suggesting flexibility between the domains. (c) The 1.45 Å resolution crystal structure of EcMscL CTD (residues 108-136) (PDB 4LKU) docked into the density map for EcMscL in MSP1D1/DMPC nanodiscs. (d) The crystal structure of the CTD was used to model this region of the full-length protein resolved by cryoEM.

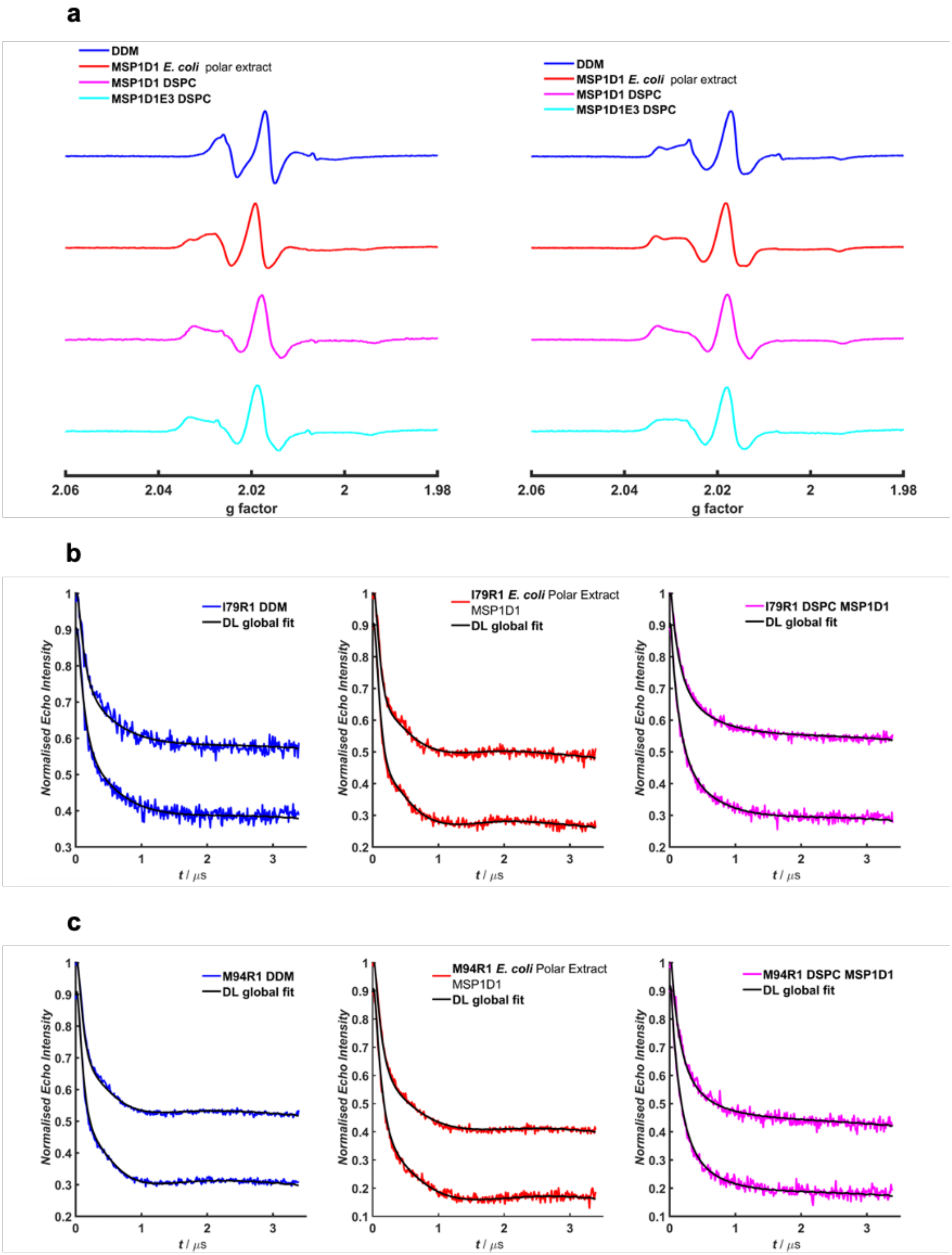

Supplementary Fig. 5: CW-EPR and PELDOR measurements of EcMscL under various conditions. (a) CW-EPR spectra of I79R1 (left) and M94R1 (right) EcMscL recorded at room-temperature under different conditions. DDM solution (blue), *E. coli* polar extract lipids (red), MSP1D1/DSPC nanodiscs (magenta) and MSP1E3D1/DSPC nanodiscs (cyan). (b-c) Raw PELDOR traces of I79R1 (top) and

M94R1 (bottom) under different conditions. The colour scheme is consistent with panel (a). Technical replicates were fitted globally and processed using compactness regularization in DeerLab (black).

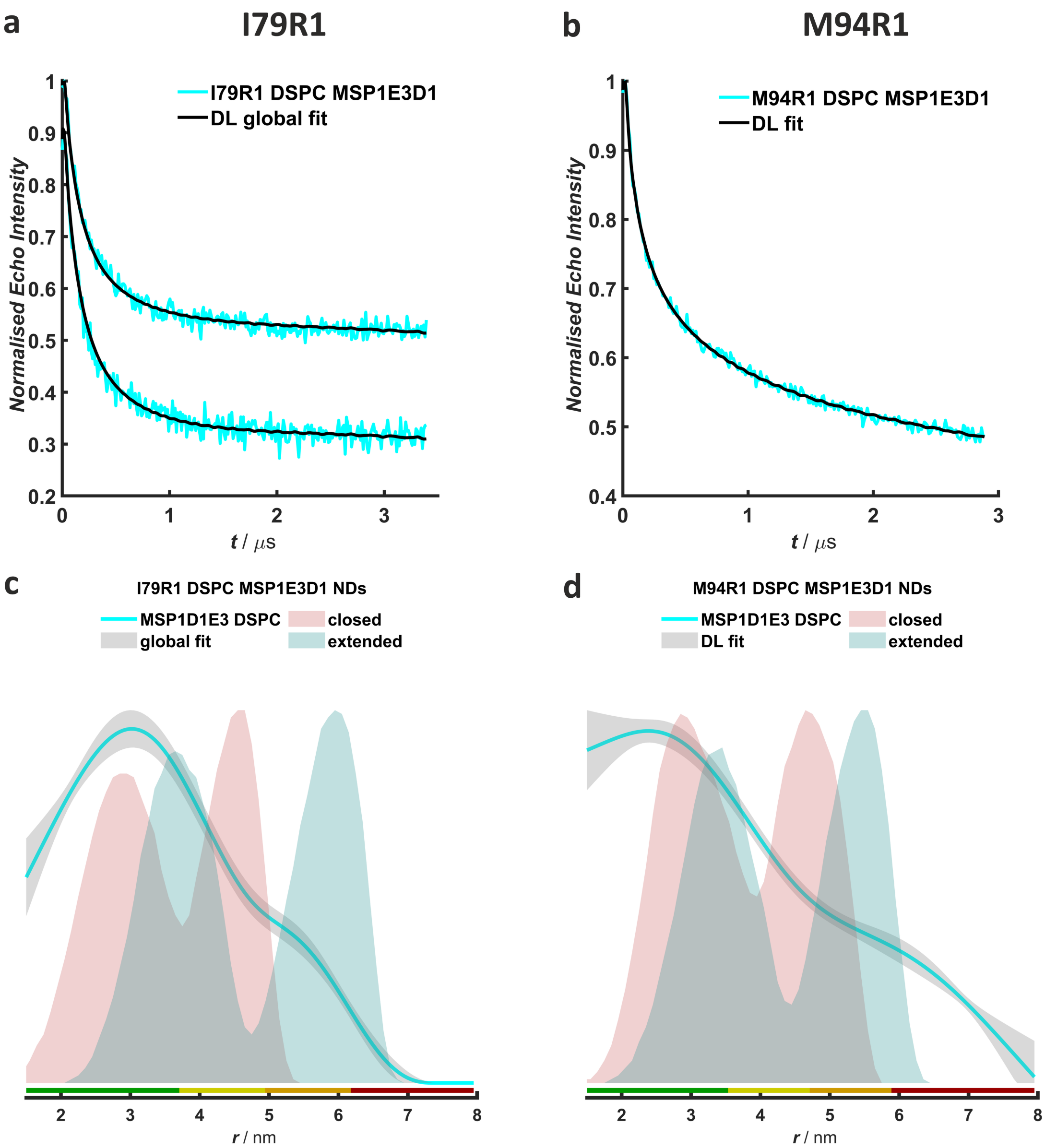

Supplementary Fig. 6: Global fitting of time-domain traces of I79R1 (a) and M94R1 (b) and corresponding distance distributions (c) and (d), in MSP1D1E3 and DSPC lipid. (a-b) Raw PELDOR traces recorded in MSP1D1E3 DSPC lipid (cyan) and corresponding Tikhonov fits (black). I79R1 was performed with or without lambda reduction (as a technical replicate), while M94R1 was performed without lambda reduction. (c-d) The corresponding DeerLab distance distributions are shown in cyan. Shaded areas (pink and teal) depict predicted distance distributions for the EcMscL closed- and expanded-state(s). The grey shaded regions are the $\pm 2\sigma$ confidence intervals, and colour bars indicate reliability. The green region denotes reliable shape, width and mean; yellow denotes reliable width and mean; orange indicates only mean reliability; and red indicates that no quantification is possible.

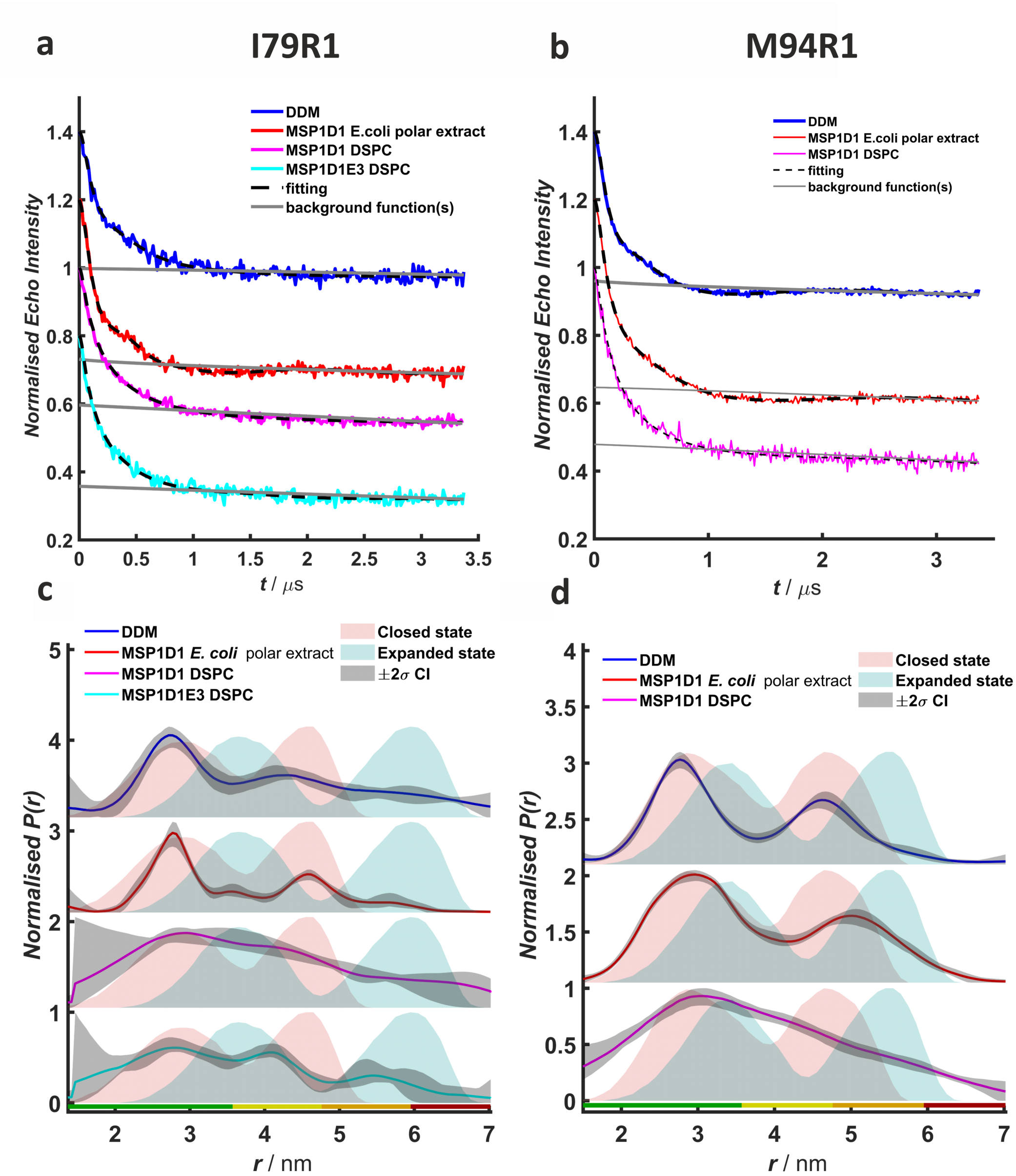

Supplementary Fig. 7: CDA analysis of PELDOR data of EcMscL I79R1 and M94R1 under different conditions with lambda reduction. (a-b) Tikhonov fitting from CDA processing. Raw PELDOR/DEER traces are shown in blue (DDM), red (MSP1D1 nanodiscs in *E. coli* lipids), magenta (MSP1D1 nanodiscs in DSPC), and cyan (MSPD1E3 nanodiscs in DSPC), with Tikhonov fits shown as black dash lines. Gray lines represent the respective background function(s). (c-d) The corresponding consensus distance distributions generated by CDA automatic analysis. Shaded areas (pink and teal) depict predicted distance distributions for the EcMscL closed- and expanded-state(s), respectively. *In silico* distance modelling was performed using MtsslWizard. The grey shaded regions represent the $\pm 2\sigma$ confidence intervals, and colour bars indicate reliability. The green region denotes reliable shape, width and mean; yellow denotes reliable width and mean; orange indicates only mean reliability; and red indicates that no quantification is possible.

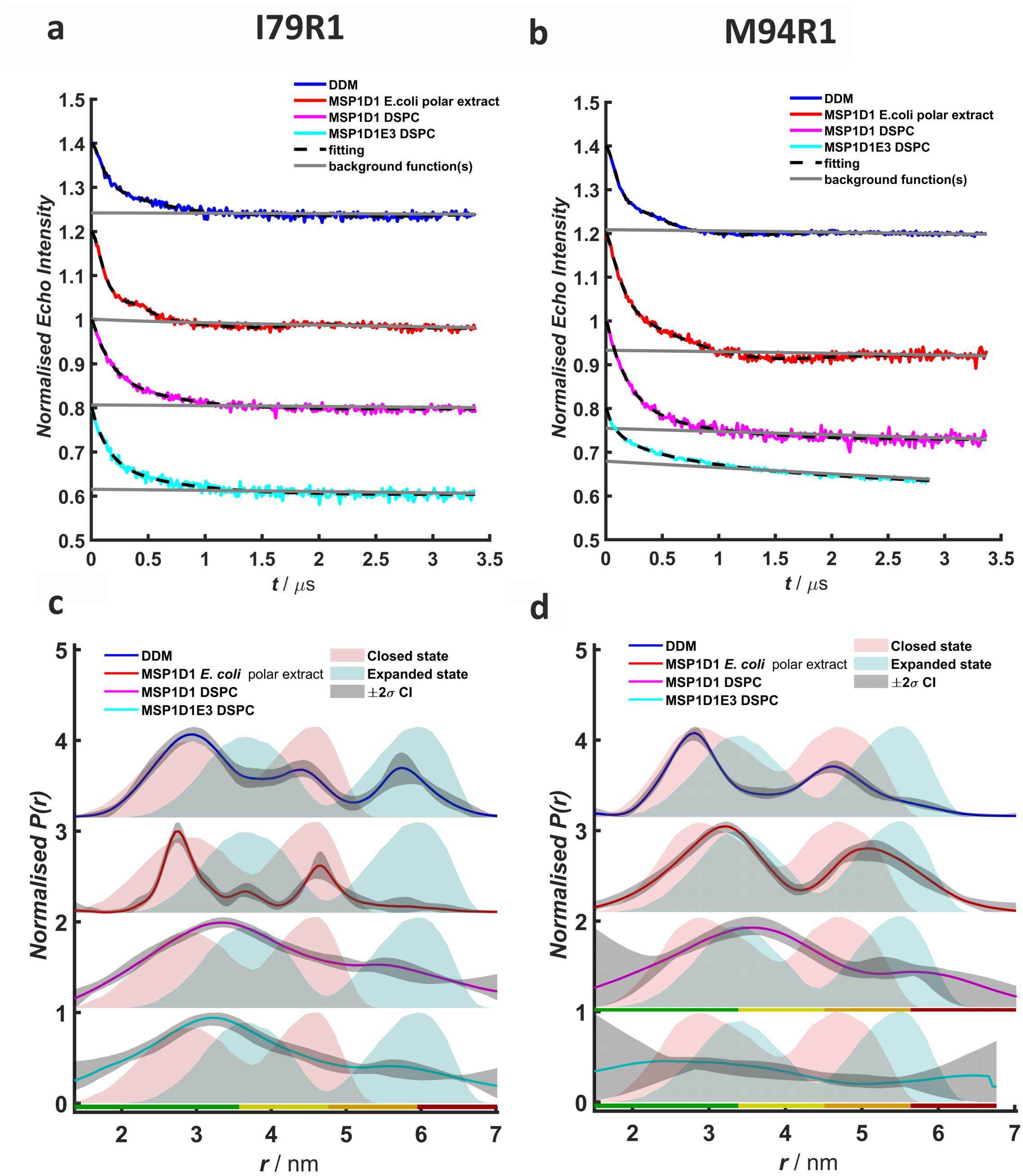

Supplementary Fig. 8: CDA analysis of PELDOR/DEER data of EcMscL I79R1 and M94R1 under different conditions. (a-b) Tikhonov fitting from CDA processing after suppression of multi-spin effects by power-scaling for $N = 5$, of I79R1 and M94R1, respectively. Raw PELDOR/DEER traces are shown in blue (DDM), red (MSP1D1 nanodiscs in *E. coli* polar extract lipids), magenta (MSP1D1 nanodiscs in DSPC), and cyan (MSPD1E3 nanodiscs in DSPC), with Tikhonov fits shown as black dash lines. Gray lines represent the respective background function(s). (c-d) The corresponding consensus distance distributions generated by CDA processing. Shaded areas (pink and teal) depict predicted distance distributions for the EcMscL closed- and expanded-state(s), respectively. *In silico* distance modelling was performed using MtsslWizard. The grey shaded regions represent the $\pm 2\sigma$ confidence intervals, and colour bars indicate reliability. The green region denotes reliable shape, width and mean; yellow denotes reliable width and mean; orange indicates only mean reliability; and red indicates that no quantification is possible.

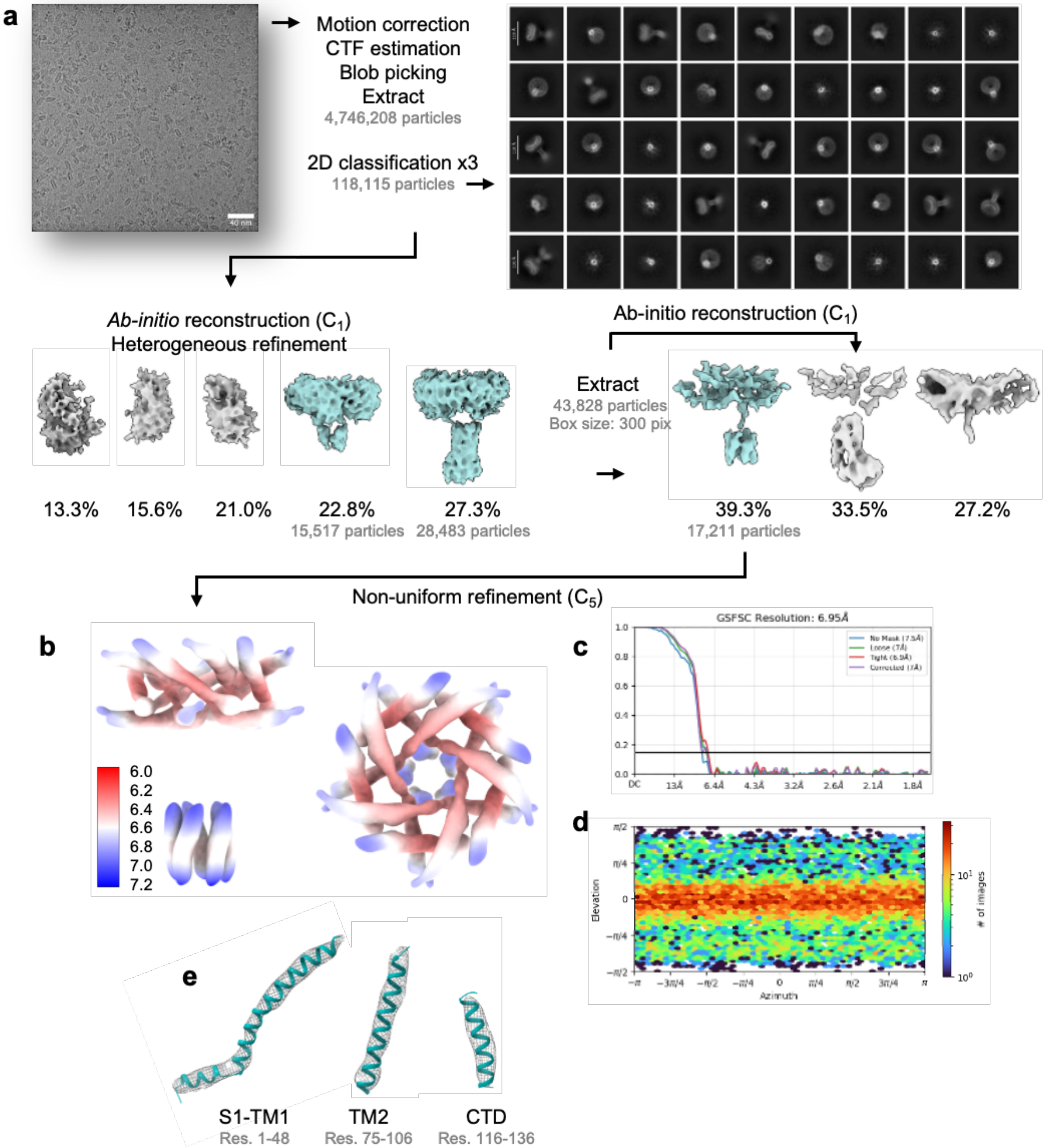

Supplementary Fig. 9: The CryoEM processing pipeline for EcMscL in MSP1D1/DSPC nanodiscs (PDB 28OC). (a) A total of 20,195 movies were motion corrected and processed in CryoSPARC, yielding 4.75 million particles after template-free picking and CTF filtering. Following three rounds of 2D classification, *ab initio* reconstruction and heterogeneous refinement, particles from the two best classes were re-extracted without binning and further classified. The final subset (17,221 particles) was refined by non-uniform refinement, producing a 6.95 Å reconstruction. (b) The 6.95 Å map coloured by local resolution. (c) Gold-standard FCS curve. (d) Angular distribution of particles in the final reconstruction. (e) Structural model of EcMscL helices S1 (residues 1-15), TM1 (residues 16-48), TM2 (residues 75-106) and CTD (residues 116-136) in the density map contoured to σ12, shown in ChimeraX.

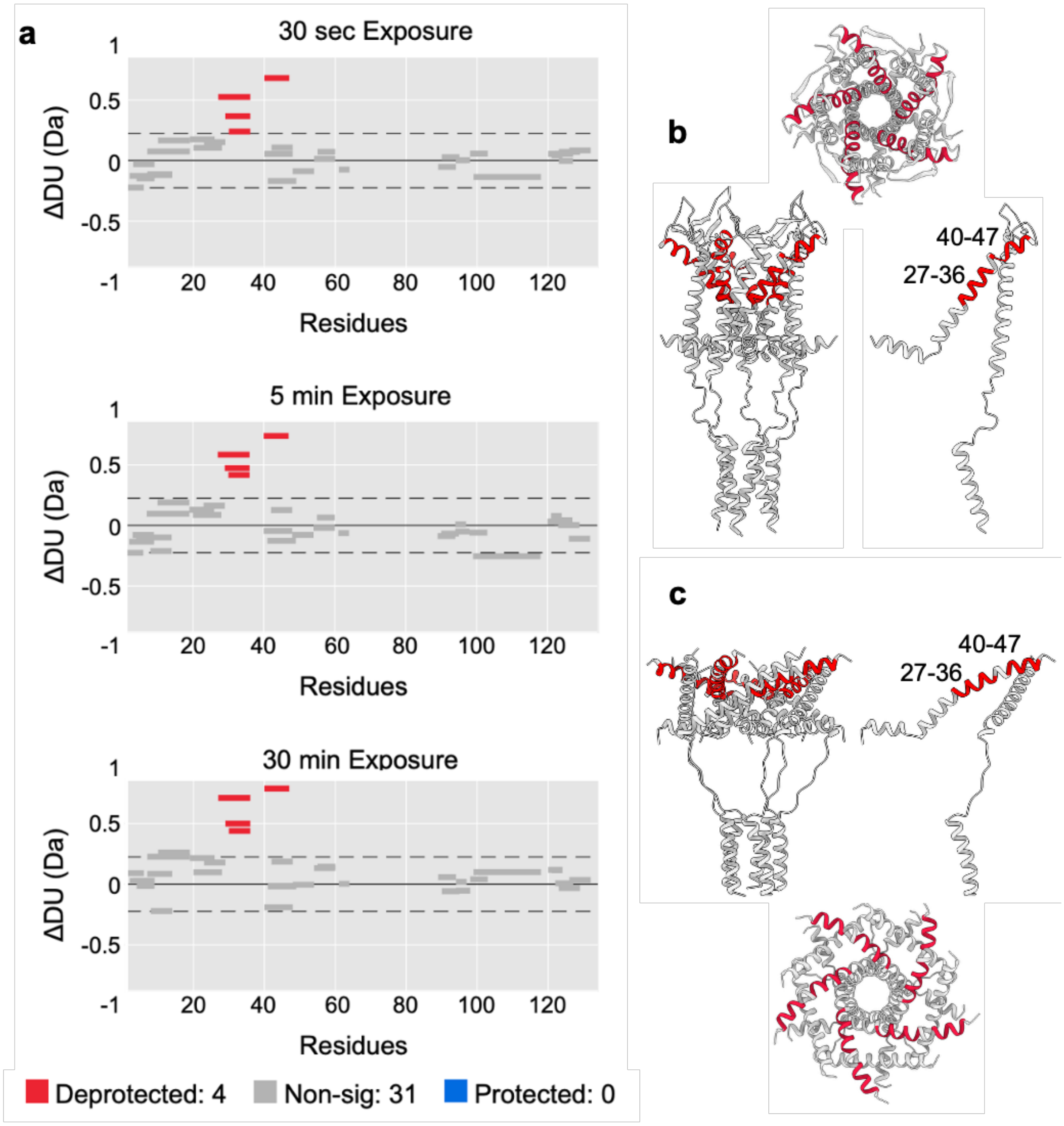

Supplementary Fig. 10: Hydrogen-deuterium exchange mass spectrometry (HDX-MS) analysis of EcMscL in DMPC or DSPC lipid nanodiscs. (a) The difference in deuterium uptake (ΔDU) between EcMscL in MSP1D1/DMPC and MSP1D1/DSPC nanodiscs at three different labelling time points (30 sec, 5 min, 30 min) are presented as Woods plots. Statistical analyses (98% confidence limit, p-value <0.02) were applied to identify peptides with significant ΔDU. Four peptides (spanning residues 27-36 and 40-47) are deprotected in DSPC nanodiscs compared to DMPC. No peptides were protected from deuterium uptake. The deprotected residues in the closed (b) and expanded (c) structures are coloured red.

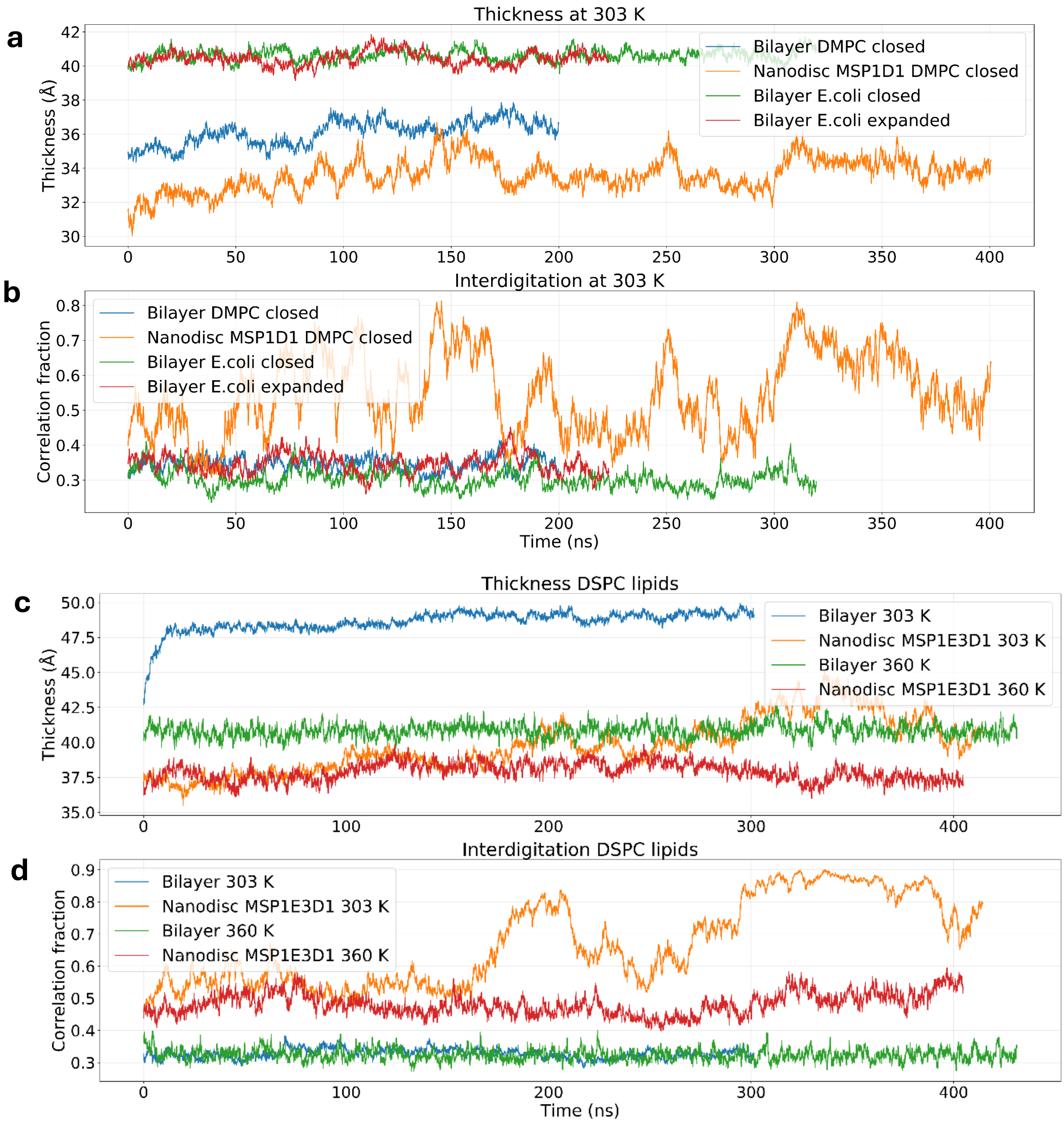

Supplementary Fig. 11: Lipid bilayer thickness and leaflet interdigitation in DMPC, DSPC and *E. coli* membrane lipid compositions. (a) Head-to-head bilayer thickness as a function of simulation time. Closed-state MscL in a DMPC bilayer is shown in blue, closed-state MscL in a DMPC MSP1D1 nanodisc in orange, closed-state EcMscL in a native *E. coli* membrane (POPE 67%, POPG 23%, cardiolipin 10%) in green, and expanded-state EcMscL in the same native membrane composition in brown. (b) Corresponding leaflet interdigitation, quantified as the correlation fraction between opposing leaflet acyl-chain density profiles. (c-d) Bilayer thickness and interdigitation, respectively, for expanded-state MscL in DSPC bilayers (blue) and DSPC MSP1E3D1 nanodiscs (orange) at 303 K, and in DSPC bilayers (green) and DSPC MSP1E3D1 nanodiscs (brown) at 360 K.

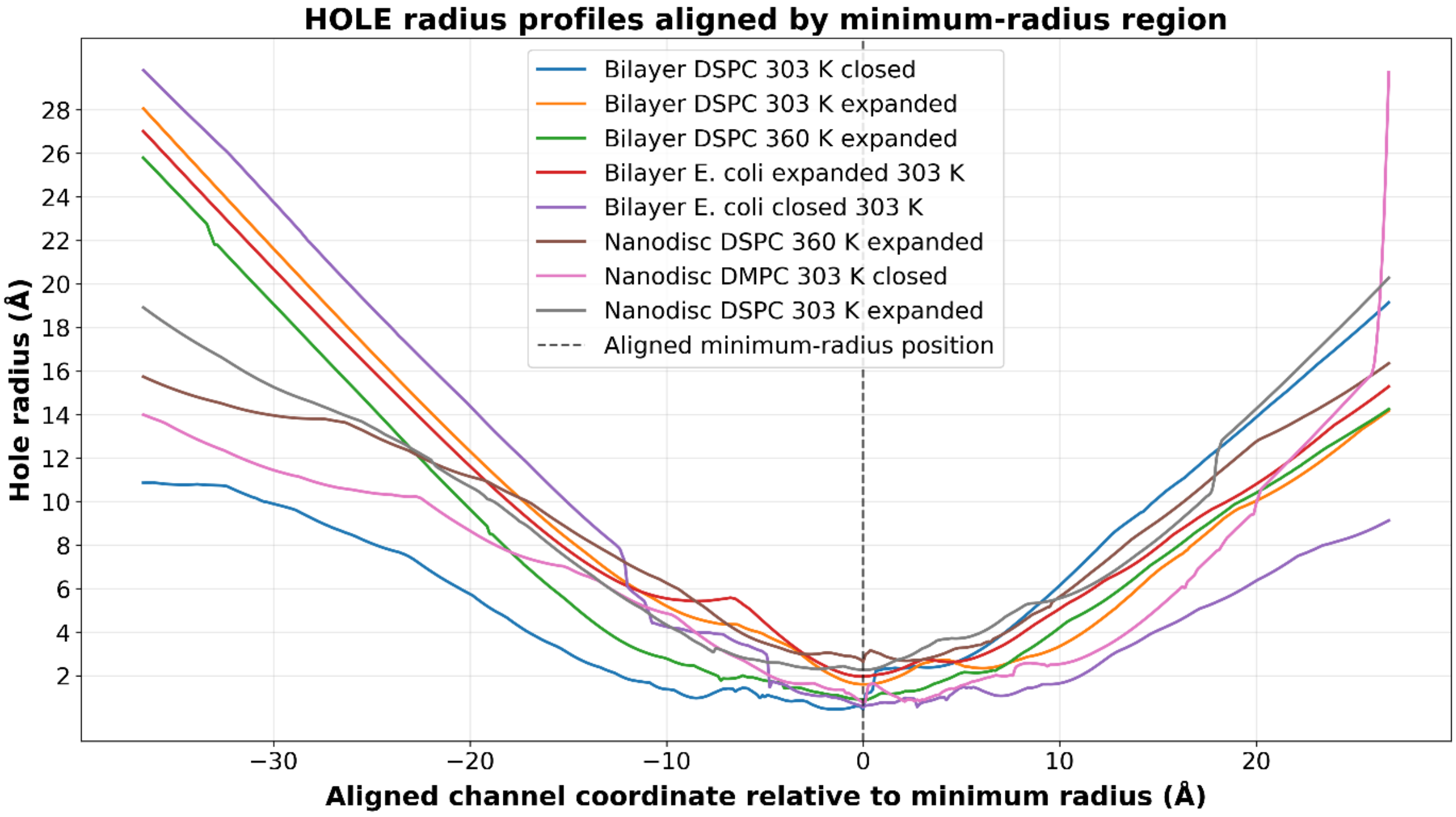

Supplementary Fig. 12: Pore radius profiles of EcMscL in different bilayer and nanodisc lipid compositions. Pore radius profiles were calculated using HOLE for the truncated EcMscL channel (residues 1–100), encompassing the transmembrane helices and periplasmic loop while excluding the cytoplasmic loops and helices. All profiles were aligned and centred on the minimum-radius pore-forming region, defined as 0 Å on the x-axis. All simulated systems exhibit some degree of pore relaxation and compression during equilibration relative to their initial expanded conformations, with most converging to a minimum pore radius of approximately 1 Å**,** indicative of a closed or highly constricted pore. In contrast, EcMscL embedded in DSPC nanodiscs at 303 K (olive) and 360 K (magenta) retains a substantially larger minimum pore radius of approximately 2.5 Å (corresponding to a pore diameter of ~5 Å), indicating that the channel remains considerably closer to the expanded state than in the other simulated systems. This interpretation is further supported by the corresponding pore hydration profiles together with the enhanced membrane thinning and lipid interdigitation observed in the DSPC nanodiscs.

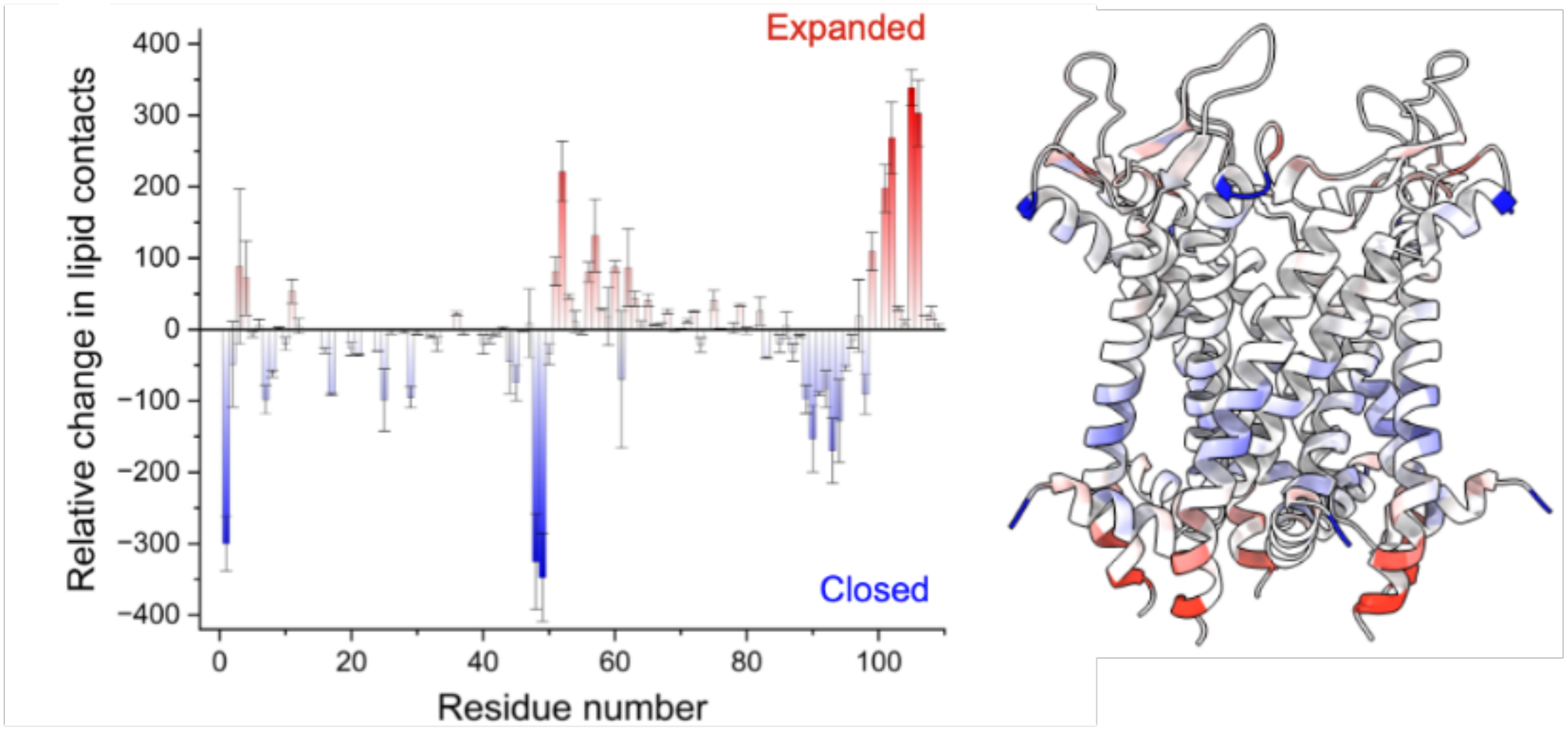

Supplementary Fig. 13: Molecular dynamics (MD) simulations of the EcMscL closed and expanded states in planar lipid bilayers. The net change in protein-lipid contacts between the closed state in DMPC (PDB 28OA) and the expanded state in DSPC (PDB 28OC) bilayers is mapped onto the closed-state EcMscL structure. The coloured the black error bars represent the average over 10 ns and bars represent the standard deviation (SD) across all the monomers and between the states.

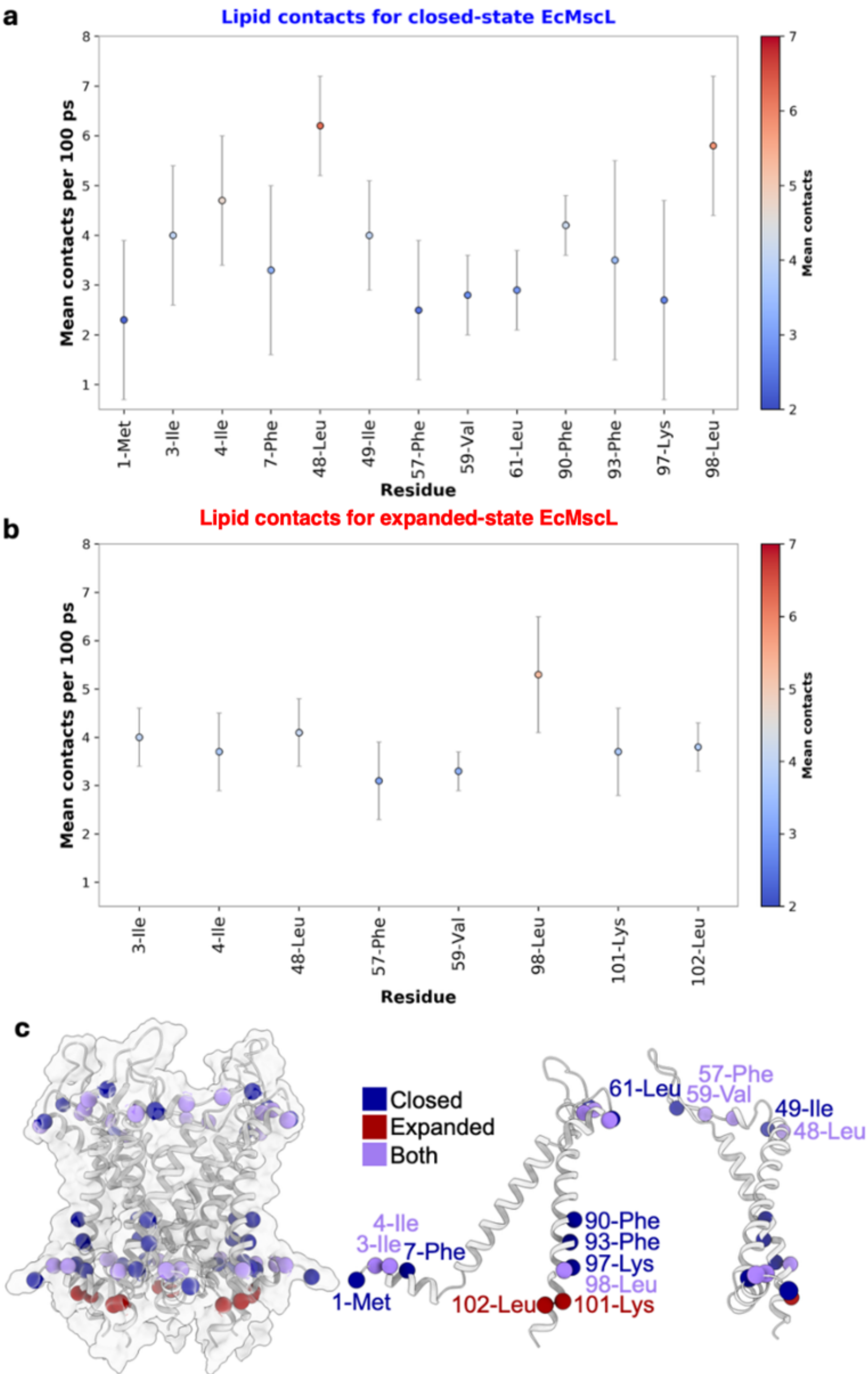
a
Lipid contacts for closed-state EcMscL
Mean contacts per 100 ps
Residue
1-Met
3-Ile
4-Ile
7-Phe
48-Leu
49-Ile
57-Phe
59-Val
61-Leu
90-Phe
93-Phe
97-Lys
98-Leu
Mean contacts
b
Lipid contacts for expanded-state EcMscL
Mean contacts per 100 ps
Residue
3-Ile
4-Ile
48-Leu
57-Phe
59-Val
98-Leu
101-Lys
102-Leu
Mean contacts
c
Closed
Expanded
Both
4-Ile
3-Ile
7-Phe
1-Met
61-Leu
57-Phe
59-Val
49-Ile
48-Leu
90-Phe
93-Phe
97-Lys
98-Leu
102-Leu
101-Lys

Supplementary Fig. 14: EcMscL residues with the highest mean lipid contacts. (a) Stabilising residues in closed-state EcMscL (PDB 28OA), which contribute the most protein-lipid contacts. (b) Stabilising residues in the EcMscL expanded conformation (PDB 28OC). The SD is calculated across the monomers. (c) Residues with the highest mean lipid contacts per 0.1 ns are mapped onto the closed-state channel. Residues unique to the closed (blue) or expanded (red) states, and residues that contribute to both states (purple) are coloured accordingly. Note that all other EcMscL residues form fewer than one protein-lipid contact per 0.1 ns.

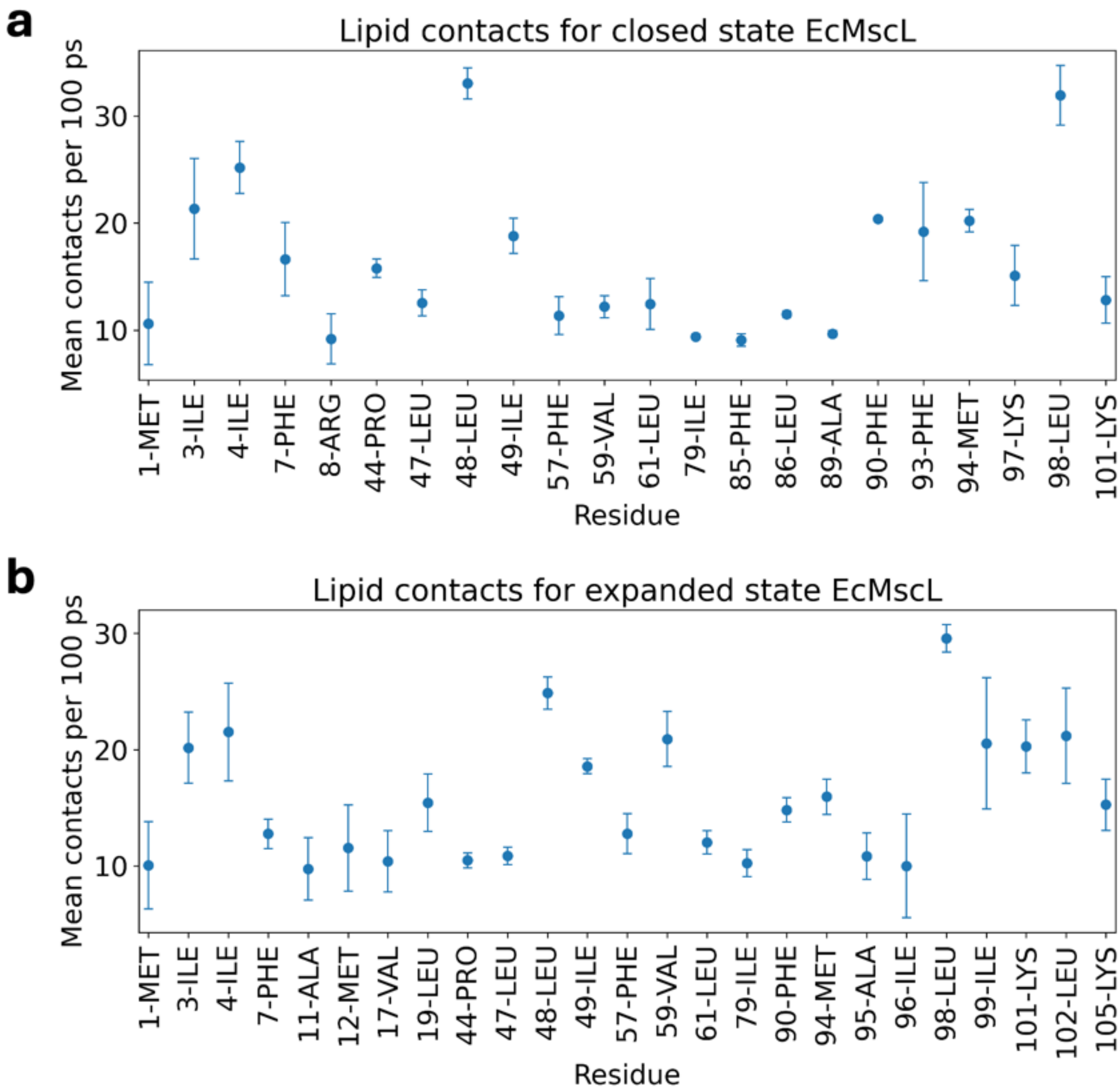

Supplementary Fig. 15: EcMscL residues with the highest mean lipid contacts in nanodiscs. (a) Stabilising residues in closed-state EcMscL, embedded in MSP1D1 nanodisc containing DMPC lipids, which contribute the most protein-lipid contacts. (b) Stabilising residues in the EcMscL expanded conformation embedded in MSP1E3D1 nanodisc containing DSPC lipids. SD is calculated across all the monomers.

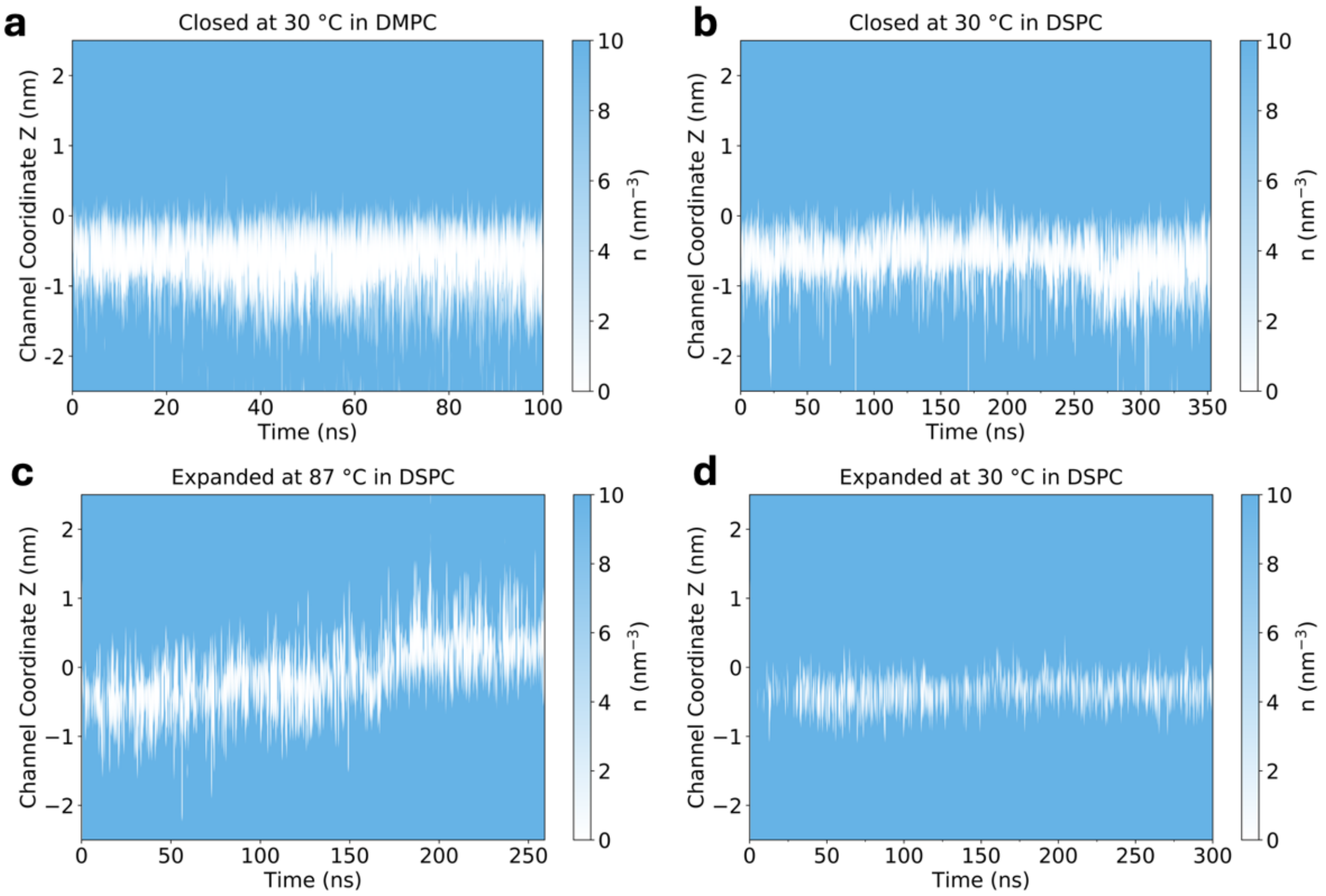

Supplementary Fig. 16: CHAP analysis of closed- and expanded-state EcMscL under different bilayer conditions. The CHAP profiles provide the water density across the channel, highlighted for the pore region as a function of time. (a-b) CHAP profiles of closed-state EcMscL in DSPC and DMPC bilayers, respectively, and at 30 °C. (c-d) CHAP profiles of expanded-state EcMscL at 87 °C and 30 °C, respectively, and in DSPC bilayers. The CHAP profiles provide the water density across the channel, highlighted for the pore region as a function of time.

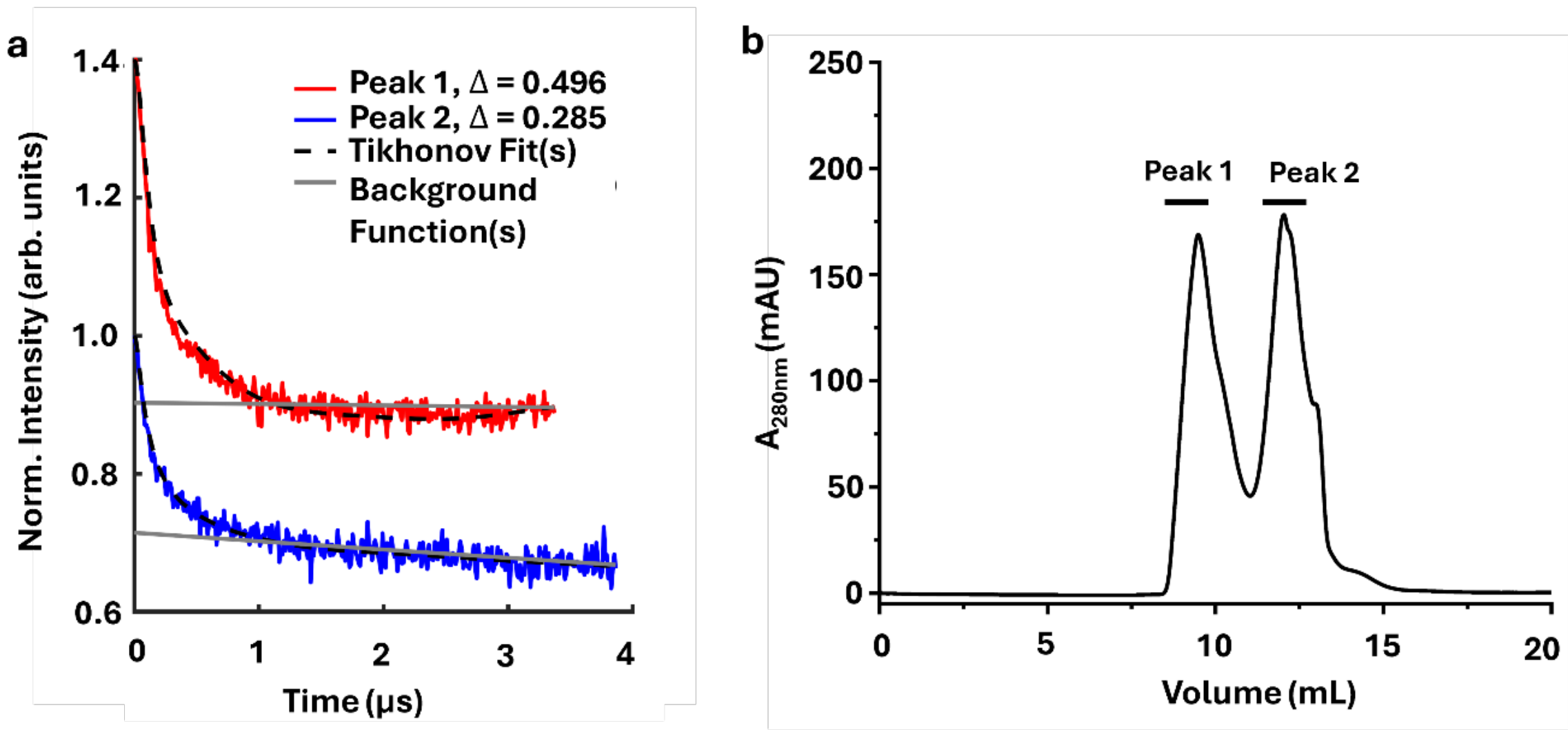

Supplementary Fig. 17: Quantitation of EcMscL labelling efficiency *via* modulation depth analysis. (a) Raw PELDOR traces of EcMscL samples prepared from distinct size-exclusion chromatography (SEC) profile peaks. (b) Representative SEC profile of EcMscL in DDM, indicating the presence of two distinct peaks, assumed to be mixtures of predominantly pentameric (peak 1) and monomeric (peak 2) EcMscL.

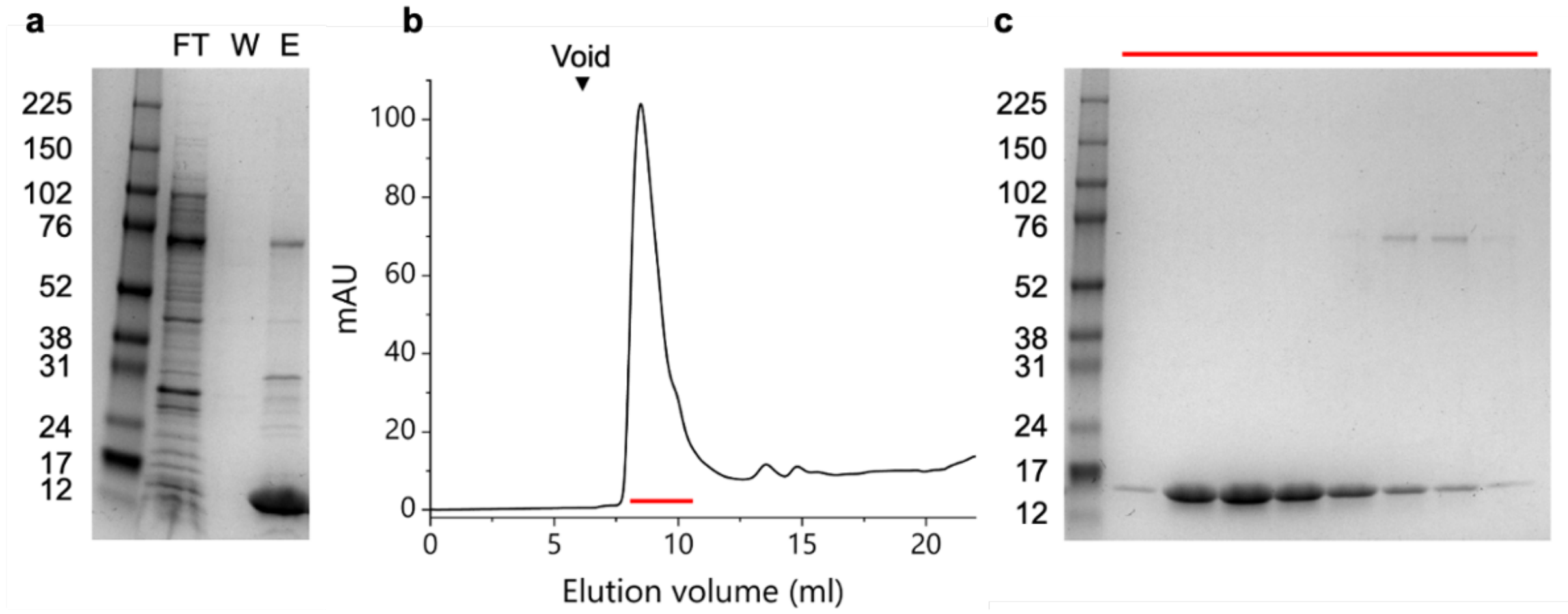

Supplementary Fig. 18: EcMscL protein purification. (a) SDS-PAGE gel of the initial IMAC purification, showing the flow-through (FT), wash (W) and elution (E) fractions. (b) The eluate was loaded onto a Superdex 200 Increase 10/300 GL column (Cytiva) for SEC. Fractions corresponding to the main peak (red line) were analysed by SDS-PAGE (c), revealing pure monomeric EcMscL (15.2 kDa). The purified protein was concentrated, then flash-frozen in liquid nitrogen and stored at -80 ℃.

Supplementary Table 1: First Wasserstein distances calculated for I79R1 and M94R1 PELDOR distance distributions from DeerLab global fitting.

	I79R1		M94R1	
Condition	Closed	Expanded	Closed	Expanded
DDM	3.54 Å	8.01 Å	1.72 Å	6.57 Å
E. coli lipids MSP1D1 nanodisc	2.28 Å	10.59 Å	2.28 Å	4.47 Å
DSPC MSP1D1 nanodisc	3.88 Å	8.21 Å	2.79 Å	5.69 Å
DSPC MSP1E3D1 nanodisc	3.18 Å	9.60 Å	4.79 Å	7.11 Å

Supplementary Table 2. First Wasserstein distances calculated as the arithmetic mean of technical replicates for I79R1 and M94R1 PELDOR distance distributions from CDA processing ($n = 2$)*. *The M94R1 DSPC MSP1E3D1 nanodisc condition was not replicated (i.e., $n = 1$).

	I79R1		M94R1	
Condition	Closed	Expanded	Closed	Expanded
DDM	1.88 Å	10.14 Å	2.77 Å	8.28 Å
E. coli lipids MSP1D1 nanodisc	2.18 Å	12.34 Å	2.21 Å	6.82 Å
DSPC MSP1D1 nanodisc	2.49 Å	11.58 Å	3.22 Å	7.83 Å
DSPC MSP1E3D1 nanodisc	3.06 Å	11.72 Å	5.17 Å*	7.91 Å*

Supplementary Table 3. Bhattacharyya coefficients calculated for I79R1 and M94R1 PELDOR distance distributions from DeerLab global fitting.

	I79R1		M94R1	
Condition	Closed	Expanded	Closed	Expanded
DDM	0.91	0.86	0.98	0.88
E. coli lipids MSP1D1 nanodisc	0.95	0.72	0.96	0.94
DSPC MSP1D1 nanodisc	0.92	0.82	0.94	0.90
DSPC MSP1E3D1 nanodisc	0.91	0.83	0.86	0.83

Supplementary Table 4: Bhattacharyya coefficients calculated as the arithmetic mean of technical replicates for I79R1 and M94R1 PELDOR distance distributions from CDA processing ($n = 2$)*. *The M94R1 DSPC MSP1E3D1 nanodisc condition was not replicated (i.e., $n = 1$).

	I79R1		M94R1	
Condition	Closed	Expanded	Closed	Expanded
DDM	0.88	0.89	0.97	0.89
E. coli lipids MSP1D1 nanodisc	0.93	0.79	0.94	0.95
DSPC MSP1D1 nanodisc	0.88	0.88	0.91	0.90
DSPC MSP1E3D1 nanodisc	0.89	0.86	0.85*	0.84*

Supplementary Table 5: Microscope parameters and model refinement statistics for all datasets

	EcMscL in DMPC<br>PDB 28OA	EcMscL in DOPC<br>PDB 28OB	EcMscL in DSPC<br>PDB 28OC
**Data collection and processing**			
Magnification	96k	165k	96k
Voltage (kV)	300	300	300
Electron exposure (e^-/Å^2)	43.7	43.0	43.0
Defocus range (μm)	-0.9 to -3.0	-0.9 to -3.0	-0.9 to -3.0
Pixel size (Å)	0.82	0.74	0.85
Symmetry imposed	C5	C5	C5
Final particle no.	121,887	53,307	17,211
Map resolution (Å)	3.90	4.31	6.51
FSC threshold	0.143	0.143	0.143
**Refinement**			
Map sharpening *B* factor (A^2)			
Model composition			
Non-hydrogen atoms	5,135	4,995	2,750
Protein residues	660	640	555
Bonds (RMSD)			
Length (Å) (# > 4 σ)	0.004 (0)	0.003 (0)	0.007 (0)
Angles (°) (# > 4 σ)	0.658 (0)	0.608 (0)	0.734 (0)
**Validation**			
**MolProbity score**	2.10	2.15	1.81
Clash score	14.72	12.50	6.42
Ramachandran plot (%)			
Outliers	0.00	0.00	0.00
Allowed	1.56	3.23	7.10
Favoured	98.44	96.77	92.90
Rotamer outliers (%)	3.64	2.80	0.00
B factors			
Protein	21.3/207.2/106.1	73.5/228.7/143.4	458.8/794.1/618.0
**Model vs. Data**			
CC (mask)	0.80	0.77	0.81
CC (box)	0.59	0.57	0.70
CC (peaks)	0.44	0.38	0.27
CC (volume)	0.78	0.76	0.82

Supplementary Table 6: HDX Data Summary Table. SD = standard deviation, CI = confidence interval

Data Set	MscL (DMPC)	Mscl (DSPC)
**HDX reaction details**	15 μM MscL, 50 mM sodium phosphate, 150 mM NaCl, pD 7.5, 4 °C	
**HDX time course (min)**	0.5, 5, 30 min	
**HDX control samples**	Maximally-labelled controls were not performed	
**Back-exchange**	Not calculated	
**Sequence coverage**	77.61 %	77.61 %
**Number of peptides**	35	35
**Average peptide length / redundancy**	7.31 / 2.46	7.31 / 2.46
**Replicates (biological or technical)**	3 (technical)	3 (technical)
**Repeatability (average SD)**	0.0539	0.0544
**Significant differences in HDX (Da)**	Control	0.22 Da<br><br>98 % Confidence, $p < 0.02$, hybrid significance test

Supplementary Table 7: Summary of the composition and simulation parameters for each system.

Structure	Lipid bilayer/nanodisc	Temp(K)	System area (Å^2)	Lipid no. (upper leaflet)	Lipid no. (lower leaflet)	Length of simulation (ns)
Closed	DMPC	303	15291.8	198	205	100
						100
	DSPC	303	15291.8	198	205	350
						250
Expanded	DSPC	303	15291.8	198	205	320
		360				430
Closed	E. *coli*	303	21747.4	307	300	400
Expanded	*E. coli*	303	22864.5	300	313	400
Closed	DMPC (MSP1D1)	303	23663.6	57	60	200
Expanded	DSPC (MSP1E3D1)	303	27852.0	111	93	400
Expanded	DSPC (MSP1E3D1)	360	27852.0	111	93	400

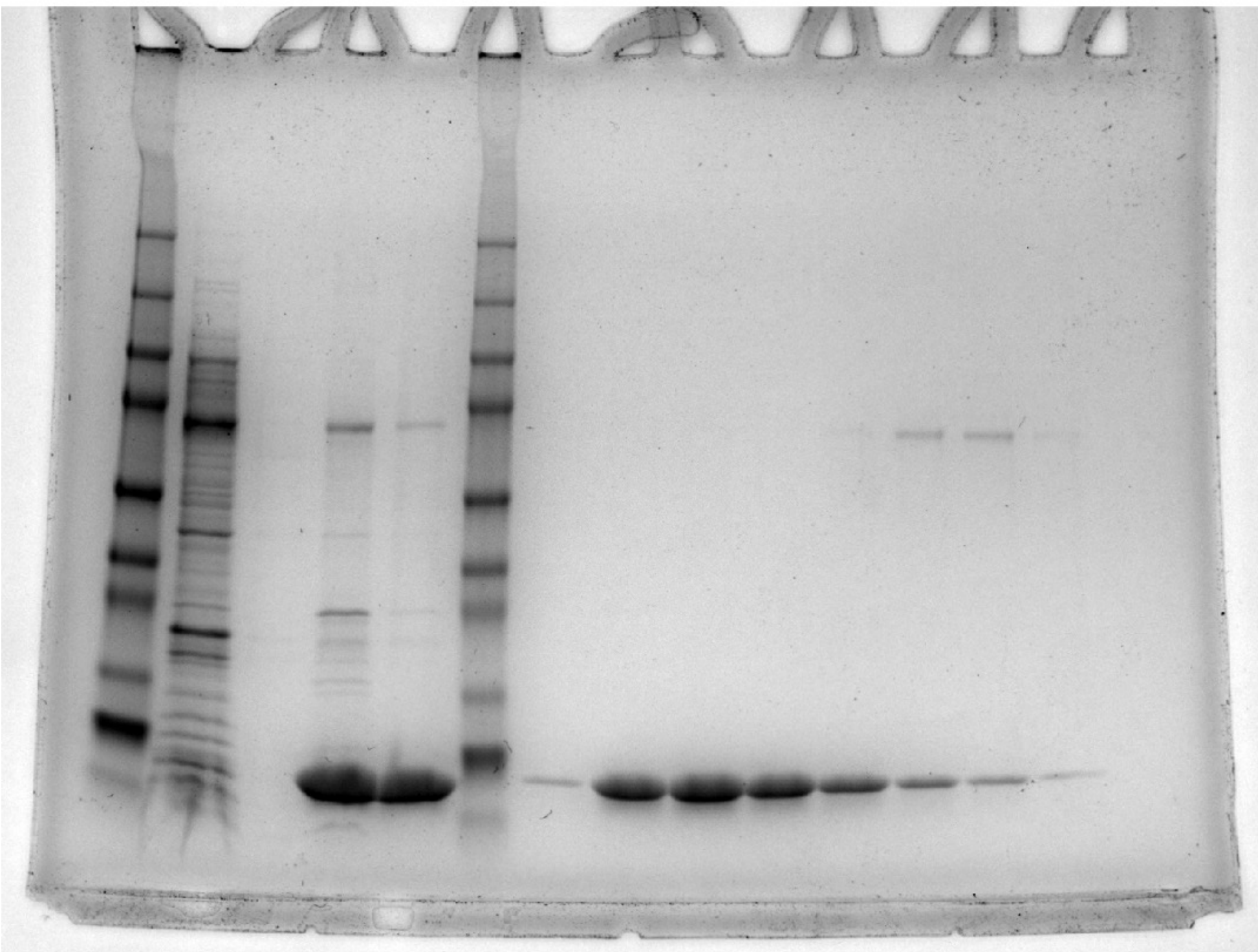

Uncropped SDS-PAGE gel (Supplementary Fig. 18)
