# Supplementary material for: Structural basis of the gating mechanism of the large-conductance mechanosensitive channel from Escherichia coli
Source: Nat Commun. 2026 Aug 22;17:10777. doi: 10.1038/s41467-026-76193-0 (PMC13661626; doi:10.1038/s41467-026-76193-0)
Supplement: Supplementary file 2 [file 41467_2026_76193_MOESM2_ESM.pdf]

nature portfolio

Peer Review File

Structural basis of the gating mechanism of the large-conductance mechanosensitive channel from Escherichia coli

Corresponding Author: Dr Christos Pliotas

Parts of this Peer Review File have been redacted as indicated to remove third-party material.

This file contains all reviewer reports in order by version, followed by all author rebuttals in order by version.

Version 0:

Reviewer comments:

Reviewer #1

(Remarks to the Author)
Key results
The authors determined the structure of MscL of E.coli (EcMscL) in different lipids stabilized in nanodiscs by electron cryo microscopy. They observed a narrow, closed channel in DOPC and DMPC. This channel closely resembled the crystal-structure of MscL from Mycobacterium tuberculosis (MtMscL, 2OAR) .
Another conformation of EcMscL with an extended cross section was seen in DSPC. Despite its modest resolution of some 6-7Å, the map clearly showed an expanded trans-membrane domain (TMD) with tilted TM-helices and a slightly widened pore.
Analysis of the two conformations with MD-simulations, "hole" and "CHAP" suggested that the narrow conformation had a closed pore that was not penetrated by water and the extended conformation was occasionally penetrated by water with an open probability of some 20%.
Further analysis with EPR spectroscopy gave distance constraints that could be mapped onto the structural models of the two conformations but did not show a strong agreement.
The authors claim that the extended conformation is related to gating of EcMscL and frequently refer to it as an "open" structure.
Significance
This is the first structure of EcMscL. It is also the first structure of MscL, which is not a crystal structure, and which shows MscL in a lipid rich environment. The structure is a major step forward in interpreting the rich functional data that was gathered on EcMscL over the last four decades.
Observing an "open" structure of any MscL would have been a breakthrough in the field. However, the represented data of the expanded conformation does not fully convince that it is really related to gating or an open structure as the pore diameter does not increase to the size that is expected for the typical, large conductance.
Data methodology and analytical approach
The cryo-EM maps are of high quality at their respective resolutions. The maps allowed building of a confident model of the closed conformation. The model of the extended conformation is also plausible, however the low resolution does not permit the identification of side chains. Therefore, deviations in the rotation around helical axes or small shifts by ½ turn along the helical axes are possible. Nonetheless, it is an exciting model which shows large conformational changes with a small expansion of the pore.
There are orthogonal tests by EPR spectroscopy, which inform on distances between labelled residues. However, the distances observed by EPR do not match the once calculated from the expanded and closed state (Figure 2b,c) For example, M84R1 in MSPD1 DSPC (Fig2C red trace) seems to be a mixture of the expanded and closed state for the short distance but do not match the expected longer distances at all. Other distance curves show even less similarity to the distances calculated from the structures. Therefore, the distance constraints contributed by the PELDOR experiments only mildly support the cryo-EM structure of the expanded state or vice versa.
This raises several questions: 1) How does the labelling for EPR change the distances in the channel? Is it really the same conformation that is observed in "EPR"-labelled and unlabelled channels? This should be checked by determining a cryo-EM map of the labelled species to align the two methods. 2) If the distance measurements represent a mixture of the

expanded and the closed state, why is this mixture not resolved in the cryo-EM maps of EcMscL in DSPC.
It is evident that in this sample the authors started with the largest dataset (> 20,000 movies, 4.5 Mio particles) and rescued the smallest percentage of particles in the final map (17 k particles). Although it is not unusual, that the majority of particles is excluded during processing of very small particles, the recovery rate is much lower than for the other two data sets. This raises the question, what is the "typical conformation" in DSPC and whether there are other conformations hidden in the data that might explain the diverging EPR profiles and might better agree with the expected larger width of a DSPC bilayer.

It is difficult to align the extended conformation with the other results and the expectancies raised by the authors. For example, they introduce that the opening threshold is lowered by shorter acyl chains (Introduction lines 109-112). This is probably why the authors chose DMPC (14:0 PC) to stabilize the open state? However, no change in the structure was observed in DMPC. The structural change occurs in DSPC (18:0 PC), which has much longer acyl chains…This is a rather unexpected observation and need some more explanation. Then they observe these very flat nano-discs in DSPC, which do not match the expected, increased width of a DSPC bilayer (see Figure S7). Do DSPC nanodiscs without MscL have the expected bilayer width or is there a problem in reconstituting these longer lipids.
Similarly, the MD-simulation snapshot (Figure 4f) shows a much larger bilayer width than what is observed by cryo-EM in nanodiscs. The authors explain that the MD simulation system of the expanded state is set-up using DSPC lipids in MSP1E3D1 nanodiscs (lines 759 – 760), but then there is no scaffolding protein visible in the system (Figure 4f) and the width of the bilayer in Figure 4F does not fit the cryo-EM structure. So, structural studies and the simulated system do not align well.

The analysis with CHAP suggests that the models in DSPC and DMPC both represent a closed conformation as the pore has a lower solvent molecule density than bulk water. In addition, the estimated pore diameter does not fit the expected large pore of an open MscL, which was shown to be large enough to release small proteins and has a conductance of several nano-Siemens. Thus, the expanded structure is incompatible with an open MscL channel. It is also unclear, whether the channel is on its way to gate or whether the expanded structure is forced by the stressed environment in the nanodisc. MSP-nanodiscs are known to alter the structure of membrane proteins and to force them into unnatural conformations (e.g. MsbA, Hoffmann et al. Structure 2025, doi.org/10.1016/j.str.2025.02.002). This possibility should be considered as an alternative to a structure in the gating transition. The authors should refrain from referring to the structure of the expanded channel as "open".

Suggested improvements
1. Lines 100-102: It is not clear which pockets are referred to in MscL. In which respect are they similar to MscS-channels. In MscS the pockets are outside of the plane of the membrane. There is no equivalent position in MscL. Please explain what you mean for outsiders.
2. Line 119-120 "75 kDa, placing it at the lower limit of current cryoEM capabilities" – is probably a bit overstated in the light of the structure of the 45 kDa NADPH oxidase (Dubach et al. 2024 NSMB). It suggests that as long as the structure is sturdy, a 75 kDa membrane protein should not be a problem for structure determination by cryo-EM. Please rephrase
3. Line 123 "… without the use of auxiliary binding partners" – In this context the MSP can be considered as an auxiliary binding partner, because the two rings of helices are clearly resolved in the map of MscL in DSPC. Please rephrase
4. Lines 133-136 – please add the full names of the lipids, their chemical composition (head group, length of acyl chain, saturation) and the rational of the choice here).
5. Lines 148-150 "minimal conformational heterogeneity" – In the CTD the side chains are not resolved, whereas they are resolved in the membrane part. This does not fit to minimal "conformational heterogeneity". Please show the variability analysis in the supplements. How was the symmetry dealt with in the variability analysis. Either explain and show or remove the statement.
6. Line 152 "only visible in the density map at a high threshold" – Should this read "lower threshold"? At "low threshold" the His-tag also becomes massive. Does the His-tag have an impact on the structure or the stability of the CTD?
7. Figure 1: It would be helpful to add some more annotations: Add the membrane spanning width in b) (I guess this is smaller than 5.8 nm?) Similarly add the membrane spanning width in Figure 3b (I guess this fits to the labelled 2.5 nm?). 1f) the pore radius is difficult to appreciate as the Y-axis does not align with pore radius 0. Perhaps add an additional thin/grey line for orientation at 0. It is also irritating that the distance on the Y-axis is longer than the 11.5 nm of the length of the channel shown in b). Which probe size was used in the pore analysis?
8. Lines 169-170 " C-terminal end of TM1 in EcMscL is tilted by ~25° compared to the equivalent region in MtMscL" – This helix is not visible in Figure 1. Please annotate and show what you refer to.
9. Lines 176-178 "The extended length of the cytoplasmic loop in EcMscL was predicted from sequence analysis, but its orientation relative to the protein was unexpected." – what was expected? Please explain in more detail.
10. Line 179 "pore radius of ~2 Å" – just to confirm… is this the radius or the diameter. It is difficult to see the distance in figure 1f and a diameter of 4 Å can be large enough to accommodate water molecules inside the pore.
11. Lines 192-193 "We hypothesised that MscL gating is primarily influenced by (a) lipid phase and order, and (b) nanodisc size. " – The hypothesis is not entirely clear to me. Please explain in more detail the rational why you think it is the phase and why it is the size of the nanodisc? … Or do you just want to anticipate the results?
12. Lines 202-203 "Here, two distinct sites on TM2 of EcMscL (Ile79R1 and Met 202 94R1) were individually labelled to serve as reporters for detecting channel expansion" – Please show the labelling efficiency of the sites. Do you expect that all 5 sites of a channel are labelled. How does the labelling alter the structure. The spin-labels appear to be very bulky and the channels are very small. Therefore, the question arises whether the labelled channel in EPR and the unlabelled channel in cryo-EM represent the same structure. This is important as the calculated and measured distances do not match(Figure 2). Perhaps check the structure of the labelled MscL by cryo-EM. (This need to be in 3D and resolve the helices… 6-7 Å resolution should be sufficient).
13. Lines 218-219 "For comparison, previous measurements of Met94R1 EcMscL in DMPC, the same lipid in which we

resolved the EcMscL closed state, showed a more homogenous distribution, in a more compact configuration"- In DMPC (Figure 2C) there are three distances. None seems to match the closed or the expanded structure observed in cryo-EM. Please explain.
14. Lines 251-252 "This state is characterised by a significant reduction in TMD thickness and a wider pore entrance" … The wider pore entrance shown in Figure 3F seems to sample different regions. It looks much more narrow in 3b/3a. than in 3f. Please make a better presentation of the pore at the narrowest site. For example, use a slice in Van der Walls representation at the narrowest site viewed perpendicular to the symmetry axis and also parallel to the symmetry axis. Make sure that side chain density is present in this representation. Otherwise the pore diameter is overestimated in the Can der Walls presentation. There is also a discrepancy between the labelling in 3f (4 Å and 8 Å) vs 2 Å and 8 Å in the figure legend. Probably radius and diameter got mixed up. Please fix.
15. Lines 279-281 "In contrast to earlier models proposing its dissociation and lateral repositioning to the membrane plane, the CTD appears to remain intact" …. But then the expanded conformation does not show a pore opening that would fit to 3-4 nS conductance. Therefore, the structure does not tell what happens when the channel opens properly. Please rephrase.
16. Line 315-315 "Although DSPC lipids form thicker bilayers than DMPC (3.6 nm vs 2.5 nm)," – The thicker bilayer does not seem to match with what is observed in the cryo-EM structure in 3a/3b. Please explain. There are also later referrals to the thicker bilayer (e.g. line 326). It would be helpful if this apparent contradiction between the expected thicker bilayer and the thinner structure would be explained.
17. Figure 4 – solvent density in b-d suggest that all three channels are closed or de-wetted. It would help to have the energy plot from CHAP to show that water can freely pass the pores in DSPC. Figure legend: Please explain what the "blue balls" are. If this is water, why are these water molecules shown and others are not shown. At which time points are the snapshots taken. The DSPC snapshot in f looks less flat than the cryo-EM structure. Did they change or is it an optical illusion? Please add more information to the figure legend and compare the MD snapshot with the starting model.
18. Discussion – Do not refer to the expanded structure as "open". This is misleading as the solvent density in the simulations does not support free passage of water. It is not even clear that the cryo-EM structure in DSPC is a structure that is relevant to gating. You might want to add some experimental evidence, which shows that the channel becomes conductive in DSPC at lower temperatures to support your claim.
19. Lines 418-420 "… speculate… more lipid depletion from the nanodiscs" – This is not entirely clear. Is there a phase transition after the reconstitution into nanodiscs?
All components are packaged simultaneously. Proteins and lipids should occupy the available space within the nanodisc. At the end of the reconstitution the nanodiscs should just package more or less lipids to form the most stable system rather than a tensed system. Why should there be more tension? In addition, the surface area is not that tightly fixed in a nanodisc as often seen in class averages of other membrane proteins.
20. Lines 434 " … although no lipids were resolved" The maps of the closed channel in DMPC is really nice and shows clear side chain density. There is no reason to believe that the map quality is not sufficient to resolve lipids. Therefore, the absence of lipids in the closed structure suggests that there are no tightly bound lipids. They rather seem to exchange and have some mobility as they are not resolved. Therefore, a model in which gating is initiated by lipids being pulled out of the pockets is not convincing based on the represented structures. Perhaps as no lipids are resolved, this is not the study that enlightens the role of lipids for the gating mechanism and should be discussed elsewhere in a different context?
21. "fewer lipids in the expanded state" – This is not visible in 4e/f please make clear which lipids in which pockets you refer to and show how the pocket size changes.
22. Figure 5b – Gating mechanism – Please add more information to the figure legend. What is the mechanism? The channel expands but the scheme does not end with an open pore of the expected size for large conductance. How do you imagine that this leads to an open pore with 2-3 nm diameter. Otherwise, this model probably does not really explain the gating mechanism and it is somewhat overstated.
23. Line 476 "upward shift" – avoid phrases like "upward" and "downward". This is confusing as there is no absolute orientation. Refer to "cytosolic" and "periplasmic" side or, towards the plane of membrane or away from the plane of membrane
24. Line 48ß-481 "partial helix" – annotate the partial helix and the three residues in Figure 1 and refer to it,
25. Add data availability statement
26. MscL Purification 535-538 – show the outcome of the purification in the SI (SEC, SDS page of fractions, which fractions have been used for analysis), same for EPR labelling experiments. Show labelling efficiency. This is really an absolutely necessary part to understand what the properties of the purified MscL were.
27. Nanodisc (lines 574-588) – What is the starting material for MscL. Did you use the sample from the SEC if so, show which fractions of the SEC were reconstituted.
28. EPR sample preparation (589-597) This involved freezing of 65 µl with glycerol. During this step every lipid sample should transform into a gel phase. So, all EPR measurements should be in gel phase. How would this effect the expected outcome/distances, when the hypothesis of the phase transition for opening is correct?
29. Lines 650-651 – Are these the two access codes of the structures determined in this study – please add in the data availability statement or give an appropriate reference.
30. Line 661 "10 eV Selectris energy filter" . it is a slit width of 10 eV – not a 10eV filter. Rephrase
31. Lines 708, 712 "at 300 Å/pixel" – this must be wrong. Do you mean 300Å width of the box?
32. Lines 758-760 … built … in nanodiscs" – is "nanodisc" true as the scaffolding protein is nowhere visible in the MD simulation snapshot in Figure 4
33. Lines 840-841 – please show the position of the deprotected residues in the structure of the closed and expanded state. Is this exposed in the periplasmic cavity?
34. lines 853-860 CHAP annotation – show delta G plots instead or together with the solvent density
35. EM workflows – indicate where C5 symmetry was applied. For example, in the ab-initio in S9 C5 was applied and in the ab-initios of the others not. The appkication of the C5 in the ab-ibitio gives rise to the funny structure in which the symmetry axis was aligned wrongly.

36. Figure S10 / lines 954 and 958 “300 Å/pixel” – please correct

Reviewer #2

(Remarks to the Author)
In this manuscript, Hardman and colleagues report the first full-length cryo-EM structures of the E. coli mechanosensitive channel MscL in both closed and expanded conformations, reconstituted in lipid nanodiscs. By combining cryo-EM with PELDOR spectroscopy, HDX-MS and atomistic MD simulations, the authors identify lipid conditions (DSPC nanodiscs) that stabilize an expanded, open-like state and characterize the associated structural rearrangements. Their data support a helix-tilt gating mechanism in which TM1 and TM2 undergo substantial radial expansion and tilting, while the C-terminal domain remains assembled as a pentamer and shifts slightly without dissociation. The integrative approach further highlights changes in protein–lipid interactions and solvent accessibility that accompany channel expansion, providing a structural framework for understanding lipid-driven mechanosensitive gating in EcMscL.
This is altogether excellent work and a very nice example of integrated structural biology. I am not in the mechanosensitive channel field and honestly thought this would have been an “easy enough” target for cryo-EM — clearly, I was naïve. Congratulations to the authors for solving this long-standing structural enigma. The lipid-dependent stabilization of distinct conformations is particularly elegant.
My main question here is more out of curiosity than as a real reservation, and concerns the choice of lipids themselves. None of them are found in the E. coli membrane lipid composition. Did the authors screen other lipid compositions, including E. coli-like ones? If yes, can they share what they observed? I understand that, for a mechanosensitive channel, the physical properties of the membrane used here were likely key to stabilizing and resolving the observed conformations. How do the authors think this opening occurs in the cell, where the lipid composition is much more complex and the membrane is crowded with other proteins? I would appreciate some speculation on this in the discussion.
Besides this, I only have minor comments.
L122. The authors rightfully highlight that the structures were obtained without the use of fiducials, nanobodies or scaffolds. What was the key factor that enabled structure determination in this case? What prevented EcMscL from being solved earlier? Was the use of nanodiscs decisive? Could the authors elaborate on what made the difference here compared to previous attempts?
L147. “The ten-amino-acid loop connecting the C-terminus of TM2 to the CTD is proline-rich (Pro109, Pro113, Pro115), reinforcing its stability.”
The statement that the proline-rich loop “reinforces its stability” is somewhat ambiguous. As prolines are typically helix-disrupting residues, could the authors clarify whether they mean reduced conformational flexibility or stabilization of a specific loop geometry?
L225. The authors state that PELDOR identified DSPC-MSP1D1 nanodiscs as favouring opening of MscL. I must admit that I do not immediately see this clearly in the PELDOR data (fig2b and c). In DMPC, the measured distances did not match the modelling of the closed state; therefore, what was the rationale for concluding that DSPC was the most promising condition for cryo-EM based on the PELDOR data? The manuscript mentions an EPR-based screen — did the authors test additional lipid compositions and/or nanodisc sizes beyond those shown? Why was DSPC selected specifically?
An another question related to the use of PELDOR on a pentameric channel. It is assumed that the changes are symmetric and coordinated? Is this the case? Do the authors observe asymmetry between the monomers in specific cryo EM subclasses?
L286. The HDX-MS data are mapped onto the structure, but I find the visualization somewhat difficult to interpret. Could the authors present the HDX differences on a representation similar to Fig. 3f, with helices clearly visible, to facilitate interpretation?

Reviewer #3

(Remarks to the Author)
The translation of mechanical force into intracellular signals (mechanotransduction) is a crucial process present across living organisms. The MscL from E. coli (EcMscL) is a paradigm example, and here the authors solved full-length structures of EcMscL for the first time. Using cryoEM, they observed both the closed and expanded structures in DMPC/DOPC and DSPC lipid nanodiscs. Further, by combining PELDOR spectroscopy, hydrogen-deuterium exchange mass spectrometry, and molecular dynamics simulations, the authors identified sites relevant for the closed-open transition. Overall, the manuscript presents novel information on the structural and mechanistic aspects of mechanotransduction and further advances the field.

Comments:
- Please state the overall resolution for the solved structures wherever relevant for clarity
- As the channel forms a homopentamer, the modulation depth of the PELDOR data must be very large, which is not the case for the presented data sets (Figure S3: modulation depth <
10% relative to a much higher expected value). Please comment on the explanations for that, including reporting the spin labeling efficiency for each variant.
- Displaying PELDOR-derived distributions exclusively from DEERNet analysis is not appropriate (despite the given justification for that). The corresponding consensus distribution must
be shown at least in the supporting information (with appropriate discussion) for the readers.
- Figure 2b-c: The conclusion that the smaller MSP1D1 scaffold shifted the conformational equilibrium toward more open or expanded states, compared to the larger nanodisc
(MSP1E3D1), is not evident. Considering that the distributions are predicted from neural network (DEERNEt) analysis and

are equally broad, a meaningful interpretation suggests a rather
similar heterogeneous distribution covering multiple states (as the authors themselves stated afterwards).
- Figure 4b-d, please define/describe the y-axis parameter in the legend
- Line 375, 'By contrast, in DSPC bilayers at 87 °C,…' this needs more clarity in the main text. Why 87 °C and what happens at lower temperatures (the stability of the protein and the
expanded structure)?
- Line 378-381: 'Together, these results indicate that temperature and the associated changes in lipid phase behaviour (Supplementary Fig. 7) strongly influence EcMscL hydration
in DSPC bilayers, leading to more frequent yet transient openings at lower temperatures.' - Complex conclusion, which is incompletely comprehensible from the results discussed.
Please explain more about how temperature, lipid phase, and hydration are correlated and the functional relevance of that.
- Line 440: 'Indeed, during our MD simulations of both states, fewer lipids were observed penetrating the pockets in DSPC bilayers compared to DSPC.' – Please refer to an appropriate
image. Also, please discuss any evidence that the authors might have seen from the MD simulations in support of the described 'lipid-moves-first'model.
- In Figure 5b, the authors need to specify the novel information on the gating mechanism obtained from the current study in relation to the existing literature.

Reviewer #4

(Remarks to the Author)
Hardman et al. present an extensive structural analysis of the structure of the E. coli MscL channel. The authors combine CryoEM, electron-electron double resonance spectroscopy and molecular dynamics simulations to characterize the closed and expanded states of the channel. The manuscript is written clearly and figures are of high quality. I outline below my questions and concerns:

1. It is very interesting and unexpected that the authors observe expansion of the channel when reconstituted in DSPC lipids. However, given what is known in the literature about the phases that saturated lipids such as DPPC (di-C16:0) and DSPC (di-C18:0) form below their transition temperatures, it seems highly unlikely that DSPC is in a gel phase in the lipid nanodiscs with MscL. The headgroup-to-headgroup distance (DHH) for DMPC in the fluid phase is ~36 A, while DHH = 44 A for gel phase DPPC (see Nagle et. al BBA 1469, 159, 2000). As such, DHH for DSPC in the gel phase would be even larger, which would be inconsistent with the ~25 A thickness of the transmembrane portion of the expanded EcMscL. The thickness of the membrane in the nanodiscs (Fig. 3a and d) is also consistent with a DHH ~25-30 A, which would not correspond to DSPC in the gel phase. Instead, the authors should consider that the DSPC lipids are forming an interdigitated phase, where the lipid tails become fully intercalated leading to significant reduction in the bilayer thickness (see Slater and Huang Prog. Lipid. Res. 27: 325, 1988; Seto et al. J Phys Soc JPN 2007; 76: 054602). The headgroup to headgroup thickness for fully interdigitated DPPC under high pressure is ~29 A (Braganza LF, Worcester DL. Biochemistry 1986; 25: 2591–6). Atomic force microscopy measurements on supported lipid bilayers of DPPC below the transition temperature have shown that lateral tension may also induce formation of the fully interdigitated phase (Vanegas et al. Langmuir 2010; 26: 10415–8). As such, it seems more likely that belt protein around the nanodisc is creating a tension that stabilizes the interdigitated phase of DSPC. I don't know if it would be possible to analyze the lipid tail and/or headgroup densities in the CryoEM maps for any signs of the interdigitated phase. For example, the area per lipid will be significantly larger compared to the fluid or gel phases.

2. Related to point 1, the authors will likely need to simulate the expanded EcMscL in the interdigitated phase of DSPC. This may need to be done under tension in order to stabilize this phase. I don't see the point in simulating the protein under high temperature in DSPC as this does not match any of the other experiments or provide physiologically relevant information.

3. The observation that the loop region that connects TM2 to the CTD is fully extended and places the CTD 25 A below the protein is somewhat striking. The authors mention in lines 147-148 that this is likely due increased rigidity from the three prolines in the loop, but there's not much of a follow up to this. The snapshot of the MD simulation in Fig. 4e shows some flexibility in the loops. I think that the rigid nature of these loops should be assessed in more depth. Is it possible that the image classification procedure is somehow 'pre-selecting' conformations where the CTD is essentially aligned with the axis of the TM region? Could the PELDOR analysis be used to characterize the flexibility of the loop region? For example one could place probes on the lower part of TM2 and the top of CTD and use this to estimate the typical distance between the CTD from the membrane.

4. Minor comment: With regards to the MD simulation parameters, the authors do not provide many details on the type of pressure coupling. I'm assuming that a semi-isotropic barostat was used, but this should be explicitly stated. For gel/interdigitated phase simulations the authors may need to use an anisotropic barostat in order to correctly model the membrane properties. The authors should also provide additional detail about the vdw/coulomb cutoffs and long-range electrostatic interactions for completeness and reproducibility. It was also not clear what criteria was used to determine the length of the simulations? What determines convergence?

Version 1:

Reviewer comments:

Reviewer #1

(Remarks to the Author)
All my concerns have been addressed appropriately.
Thanks for the insightful replies.

Reviewer #2

(Remarks to the Author)
Overall, I think the revised manuscript is significantly improved. Both the other reviewers and are to be thanked for this work. The authors have performed a lot of additional work, new PELDOR measurements in native-like E. coli lipid nanodiscs and additional MD simulations. These new data help clarify an important point of the initial submission, namely whether the DSPC nanodisc condition reflects a meaningful shift in the conformational ensemble.
I also appreciate that the authors have softened the interpretation of the DSPC structure. The manuscript now more clearly describes this conformation as an expanded, open-like, or sub-conducting intermediate rather than as a fully open MscL channel.
The additional PELDOR data in E. coli polar lipids are very useful. They support the idea that the MSP1D1 scaffold alone does not force EcMscL into an expanded conformation, since the channel appears predominantly closed in a native-like lipid environment. The new MD simulations also provide a more plausible explanation for the behaviour of DSPC nanodiscs, especially through bilayer thinning and interdigitation. I still think that this remains a somewhat artificial condition but the authors now discuss this more transparently and no longer overstate it as a direct mimic of the fully tension-gated open state.
I therefore think that the manuscript is totally suitable for publication.

Minor comments:

- There are now both Supplementary Figures and Supporting Information, which makes the manuscript a bit confusing to navigate. It would be helpful if the authors could harmonize this, using either “Supporting” or “Supplementary”. Also, the information presented in the supporting information is not consistently referenced to in the main article file.
- The caption of Supporting Figure 6 still mentions an “open” state instead of an “expanded” state.

Reviewer #3

(Remarks to the Author)
The new experiments and analyses addressing my concerns about PELDOR data acquisition and analysis are highly welcome and significantly improve the coherence of the manuscript. However, a few information discussed in the authors' response are currently missing from the manuscript and supporting information. The authors are strongly enciuraged incorporate these details into the final files.

1) Regarding the query on the modulation depth of the PELDOR data, authors state that that they performed PELDOR experiments from two distinct peaks of the SEC profile to estimate the pentamer fraction and labeling efficiency. However, neither the SEC profile nor the PELDOR data for the second peak (peak 2) are presented.

2) For the determination of the distances from PELDOR data, now authors present output from the DeerLab program. Please briefly explain in the methods, how the multi-spin effects were accounted for during this analysis specific in the methods section. Authors mention that the output from the Consensus Deer Analyzer (CDA) is presented in the supporting information, which cannot be found and must be added.

Reviewer #4

(Remarks to the Author)
While the authors have addressed many of the issues I brought up in my earlier review, I have some new questions/comments from the substantial changes/additions to the manuscript.

The subsection about the bilayer thickness and interdigitation (lines 439-455) feels like an afterthought at the end of the Results section. Given the importance of using DSPC in the nanodiscs to capture the expanded state of EcMscL, I think that the possible mechanisms to why a long-chain, high melting T, lipid could induce a protein state characterized by a reduced membrane thickness should be presented early on. The results presented starting at lines 190 through 308 are completely focused on the protein features and ignore what's happening in the surrounding membrane environment. The authors need to clearly state early on that the nanodiscs formed by DSPC cannot possibly be in the conventional gel or liquid crystalline phases as the thickness (~25 A) shown by the CryoEM maps suggests otherwise. E.g., in lines 342-343 the authors state "Although DSPC lipids form thicker bilayers than DMPC (3.6 nm vs 2.5 nm), our results identified two regions of increased deuterium uptake in DSPC, spanning residues 27-36 and 40-47 (Fig. 3h)." This statement hints at that there is something unusual going on with the DSPC nanodiscs, but at the same time the first part implies that the bilayer thickness of DSPC 'should' be larger. My point is that it's confusing and somewhat deceiving to present the results of the expanded state in DSPC assuming that it always forms a thick gel phase membrane and then at the end say nevermind it was likely an interdigitated phase all along!

I think the authors should not refer to DSPC as gel-phase lipids (e.g., line 484) and instead use the term high melting T or high transition T lipids to avoid confusion that DSPC always forms a gel phase at physiological or room temperatures.

Additionally, the bilayer thickness and interdigitation subsection seems to only refer to the simulation results to support the idea that the DSPC nanodiscs are interdigitated, but the CryoEM maps also provide strong support for this given the observed hydrophobic thickness.

Given the time evolution of the channel in the MD simulations (e.g., Fig. Supp. 6), where the system may be transitioning from an expanded to a closed state over time. I don't think it is appropriate to make statements about the percent of time spent in a particular configuration or generate probabilities (e.g., lines 403-425) as this assumes that the simulations are exploring a well-sampled equilibrium. This would require running much longer simulations with multiple replicas. It's ok to describe the time evolution and make statements about how it only remains in a particular state for some period of time out of a long simulation, but presenting percentages and probabilities requires much more rigorous sampling.

The newly added paragraph at the end of the discussion section (lines 491-509) seems highly speculative about how lipid removal from crevices and the 'lipids move first' could be playing a role in the transition to the expanded state. The resolution of the CryoEM maps does not allow the authors to directly probe this hypothesis. The authors state in line 504 "Notably, the hydrophobic pockets lining the cytoplasmic side of EcMscL reduce in volume when the channel expands, suggesting they can accommodate fewer lipids in the expanded state (Fig. 4e-f)." Yet the correlation between the crevices not accommodating lipids in the expanded state does not have to be the cause for the channel to transition to this state.

I believe there's a typo in line 494 "however, in the case of EcMscL51,100 and the MscS-like EcYnal101 which both have substantially higher tension activation thresholds compared to EcMscL," should the first be EcMscS?

Open Access This Peer Review File is licensed under a Creative Commons Attribution 4.0 International License, which permits use, sharing, adaptation, distribution and reproduction in any medium or format, as long as you give appropriate credit to the original author(s) and the source, provide a link to the Creative Commons license, and indicate if changes were made.
In cases where reviewers are anonymous, credit should be given to 'Anonymous Referee' and the source.
The images or other third party material in this Peer Review File are included in the article’s Creative Commons license, unless indicated otherwise in a credit line to the material. If material is not included in the article’s Creative Commons license and your intended use is not permitted by statutory regulation or exceeds the permitted use, you will need to obtain permission directly from the copyright holder.
To view a copy of this license, visit https://creativecommons.org/licenses/by/4.0/

Response to reviewer's comments

Reviewer #1 (Remarks to the Author):

Key results

Reviewer: "The authors determined the structure of MscL of *E. coli* (EcMscL) in different lipids stabilized in nanodiscs by electron cryo microscopy. They observed a narrow, closed channel in DOPC and DMPC. This channel closely resembled the crystal structure of MscL from Mycobacterium tuberculosis (MtMscL, 2OAR).

Another conformation of EcMscL with an extended cross-section was seen in DSPC. Despite its modest resolution of some 6-7Å, the map clearly showed an expanded trans-membrane domain (TMD) with tilted TM-helices and a slightly widened pore. Analysis of the two conformations with MD-simulations, "hole" and "CHAP", suggested that the narrow conformation had a closed pore that was not penetrated by water and the extended conformation was occasionally penetrated by water with an open probability of some 20%.

Further analysis with EPR spectroscopy gave distance constraints that could be mapped onto the structural models of the two conformations but did not show a strong agreement. The authors claim that the extended conformation is related to gating of EcMscL and frequently refer to it as an "open" structure."

Significance

Reviewer: "This is the first structure of EcMscL. It is also the first structure of MscL, which is not a crystal structure, and which shows MscL in a lipid-rich environment. The structure is a major step forward in interpreting the rich functional data that was gathered on EcMscL over the last four decades."

Reply: *We appreciate that the reviewer recognises the importance of the long-standing challenges in the field and the large and wide impact of our findings in successfully solving the first structure of the EcMscL channel, one of the most studied and highly decorated mechanosensitive channel proteins.*

Reviewer: "Observing an "open structure" of any MscL would have been a breakthrough in the field. However, the represented data of the expanded conformation does not fully convince that it is really related to gating or an open structure, as the pore diameter does not increase to the size expected for the typical large conductance."

Reply: *Indeed, a fully open MscL state consistent with a ~3 nS conductance is yet to be determined, and we agree this would constitute a key breakthrough in the field. We note that the expanded state we first resolved here corresponds to an intermediate sub-conducting state, rather than a fully open structure and this is still a significant step forward as it allows to study EcMscL gating at a molecular level. Electrophysiology studies of MscL suggest that the channel adopts at least four sub-conducting states (Sukharev et al. 1999). In this regard, our structures, which resemble both the closed and sub-conducting expanded states, contribute to understanding the first crucial steps of this channel's transition (i.e., along the pathway before full pore opening is achieved). We acknowledge that we have not obtained experimental data for the fully open state, and we have amended the text accordingly to reflect the sub-conducting "expanded" state we obtained.*

Data methodology and analytical approach

Reviewer: "The cryo-EM maps are of high quality at their respective resolutions. The maps allowed building a confident model of the closed conformation. The model of the extended conformation is also plausible; however, the low resolution does not permit the identification of side chains. Therefore, deviations in the rotation around helical axes or small shifts by ½ turn along the helical axes are possible. Nonetheless, it is an exciting model which shows large conformational changes with a small expansion of the pore."

Response to reviewer's comments

Reply: *We thank the reviewer for acknowledging that our maps are of high quality and provide reliable models, given the limitations imposed by the respective resolution of each map. We agree that the lower resolution of the expanded state does not permit side chain identification. The observation that the CTD shifts "one full helix turn" closer to the membrane upon channel expansion was made by measuring the distance between the terminal ends of TM2 and the CTD (residues 106-116) in the closed and expanded states. As the distance reduces by ~5 Å, we equated the change to a helix turn; however, we now amend the text given that we cannot confidently observe side chain density in this region. Further, in our HDX-MS analysis, we state that TM2 "tilts and rotates to facilitate pore expansion". We acknowledge that both HDX and our expanded state cryoEM structure cannot fully resolve helical rotation, and we have now amended the revised manuscript accordingly to reflect this.*

Reviewer: "There are orthogonal tests by EPR spectroscopy, which inform on distances between labelled residues. However, the distances observed by EPR do not match the ones calculated from the expanded and closed state (Figure 2b,c). For example, M84R1 in MSPD1 DSPC (Fig 2C red trace) seems to be a mixture of the expanded and closed state for the short distance but do not match the expected longer distances at all. Other distance curves show even less similarity to the distances calculated from the structures. Therefore, the distance constraints contributed by the PELDOR experiments only mildly support the cryo-EM structure of the expanded state or vice versa."

Reply: *We thank the reviewer for their comment and agree in part with their statement. We have now performed multiple additional PELDOR measurements of I79R1 and M94R1 EcMscL in DDM, as well as in MSP1D1 nanodiscs using E. coli polar extract lipids, which constitute EcMscL's native lipid membrane environment. We performed these measurements as a control, because EcMscL should be in the closed state in native lipid conditions. Indeed, we observe better agreement between experiment and prediction upon global fitting of technical replicate data. As such, we have now reconstructed Fig. 2 using new data to prioritize comparison within MSP1D1 nanodiscs between E. coli native lipid environment and DSPC lipids, as we find DSPC alters the conformational equilibrium (when compared to the native lipid environment) more dramatically than a specific nanodisc. We now move the MSPD1E3 nanodisc condition for both EcMscL modified mutants to the SI and removed the previously reported measurement of MSP1D1 nanodisc in DMPC lipid, instead citing the primary research in the text. We also note that PELDOR is an ensemble method, meaning it captures all possible states present in a protein sample, even in cases when these cannot be fully resolved (i.e., broad distance distributions). In contrast, cryoEM is a single particle technique, which is partially "biased" towards high resolution, and resulting in cases like the ones the reviewer mentioned (i.e., 4.5 M particles initially picked and only 17 K particles used in the final structure, therefore only ~0.3% of particles used). We agree with this excellent point and explain in our other responses below. We now revise the manuscript to clarify and reflect these key points.*

Reviewer: "[…]This raises several questions

How does the labelling for EPR change the distance in the channel? Is it really the same conformation that is observed in "EPR-labelled" and unlabelled channels? This should be checked by determining a cryo-EM map of the labelled species to align the two methods."

Reply: *We appreciate that this is only raised as a possibility, but we respectfully disagree. EPR combined with site-directed spin labelling (SDSL) is a powerful and highly established high-resolution method which has provided key insights in addressing the dynamics of a large variety of membrane protein classes across life kingdoms (Shah et al., 2025). Notably, conformational changes (i.e. in the range of angstroms) were accurately detected by PELDOR,*

much smaller than changes associated with EcMscL. Moreover, Perozo, Martinac and co-workers have previously spin labelled the same EcMscL protein at the same labelling positions used here (L79C and M94C), using the same spin label (MTSSL), combined with CW-EPR spectroscopy to structurally characterise EcMscL in lipids. Further, an extended screen of EcMscL implemented by Bount and co-workers (using over 60 single-Cys EcMscL mutants modified with [2-(Trimethylammonium)ethyl] methane thiosulfonate bromide (MTSET)), demonstrated that after labelling with MTSET at positions L79R1 and M94R1, no change in EcMscL behaviour in osmotic shock cell viability assays was observed with respect to WT EcMscL (Iscla et al. 2015). This suggests that the function of these mutants is not altered in native cellular conditions, and notably, MTSET is sterically bulkier (and thus potentially more structurally perturbative) than MTSSL (Bartlett et al. 2004) and still induces no functional changes to this particular channel.

We took account of these results when first designing our study and carefully selected our labelling sites, so that these mutations (and consequently modifications) would be minimally disruptive to the structure and function of EcMscL. Further, in our previous study on MtMscL (Kapsalis et al. 2019), we showed that for more than a dozen TM sites, PELDOR distances were within 1-2 Å of in silico modelling, again suggesting the conformation of TbMscL (with a similar architecture to EcMscL, as noted by the reviewer), is not altered. These findings were also confirmed by electrophysiology in GUVs (Kapsalis et al. 2019). Indeed, MTSSL is comparable in size to an amino acid (i.e. His) and is minimally disruptive, especially compared to fluorophores used for interrogating such changes in integrative membrane proteins. This is unlike FRET, which is widely used to report conformational changes, despite suffering from large orientational factor uncertainty and potentially inducing structural asymmetries to symmetric oligomeric complexes (i.e. a donor-acceptor pair is required compared to a single spin label type in EPR).

Further, we minimise systematic error by including two distinct sites, consistent with the white paper of the EPR community (Schiemann et al., 2021). We previously obtained a high-resolution X-ray structure of the spin-labelled EcMscL channel (Pliotas et al. 2015), and even before determining this structure, demonstrated sub-Angstrom resolution in the resulting PELDOR distances (Pliotas et al. 2012). Finally, even in the case we obtain cryoEM structures of spin labelled MscL on these two sites, present incomplete labelling under those conditions will "contaminate" the resulting cryoEM map densities for the resolved ("spin labelled") EcMscL state compared to the non-modified mutant channel (i.e. the aim of the reviewer's suggested experiment), adding ambiguity and leading to inconclusive data and conclusions. Nevertheless, we now amend the text in the revised manuscript to summarise these points and we also cite Martinac/Perozo and Blount studies to rationalise how these particular labelling sites were selected for PELDOR.

Reviewer: "If the distance measurements represent a mixture of the expanded and the closed state, why is this mixture not resolved in the cryoEM maps of EcMscL in DSPC?"

Reply: *PELDOR is an ensemble method and thus captures more heterogeneity (i.e., more states) present in solution than observed by cryoEM. This is a unique strength of PELDOR and our multidisciplinary approach to tackle a single problem. Indeed, cryoEM being a single particle method only captured the dominant state of EcMscL, while PELDOR informed on additional expanded/sub-opened and closed states within the equilibrium, which shifted between different lipid and MSP scaffold conditions. Notably, the flash-freezing rates of cryoEM and PELDOR sample preparation can differ by more than an order of magnitude, with cryofixation in liquid nitrogen progressing on the high-ms timescale. As the transition from the closed state to the sub-conducting S1 state of EcMscL is rate limiting (on the high-ms timescale), with additional transitions progressing much more quickly (Sukharev et al. 1999),*

the relative heterogeneity observed by PELDOR is expected, owing to the slow rate of sample freezing when compared to cryoEM cryofixation (Haubner et al., 2025).

Reviewer: “It is evident that in this sample, the authors started with a large dataset (>20,000 movies, 4.5 Mio particles) and rescued the smallest percentage of particles in the final map (17 K particles). Although it is not unusual that the majority of particles are excluded during the processing of very small particles, the recovery rate is much lower than for the other two datasets. This raises the question, what is the “typical conformation” in DSPC and whether there are other conformations hidden that might explain the diverging EPR profiles and might better agree with the expected larger width of a DSPC bilayer.”

Reply: *We thank the reviewer for their comment. Please also see the response above. As the reviewer rightly pointed out, we collected a significantly larger dataset for this condition, and as a result, picked more initial particles. One key difference between the DSPC dataset and DMPC or DOPC was that there were a larger number of empty nanodiscs present in the sample. This occurred despite the reconstitution method and MSP type being consistent throughout, suggesting the lipid itself was the main differential to reconstitution efficiency. As a result of the larger number of empty nanodiscs, just 2.5% of the initially picked particles were selected after three rounds of 2D classification (118 K of 4.7 M particles). However, as the reviewer will note, particle retention thereafter (for subsequent ab-initio, heterogeneous refinement and extraction jobs) was comparable to that from DMPC or DOPC. For example, for DMPC, the final particle number is 10.4% of the total particles following initial 2D classification (121,887 of 1,117,542), and for DSPC, it is 9.4% (17,221 of 118,115). For DOPC, this number is higher (52,307 of 257,125 or 20.3%) (Supplementary Fig. 8-10).*

The cryoEM dataset for the DSPC condition was very challenging to process because it was significantly more heterogeneous than DMPC and DOPC. For DSPC, we ran almost 500 individual jobs in CryoSPARC, resulting in many diverging and independent processing pathways. In our data, we resolved several 2D and 3D class averages of “less open” (albeit not closed) species, which we were not able to resolve to high resolution, despite our efforts. For example, please refer to the updated main text Fig. 2 detailing the 2D and 3D classes for 310,613 particles. While the largest class (pink; constituting 21% of the particles) represents the expanded state we solved to 7 Å, at least two more less-open structures (orange and purple) were present within the dataset. Please note that these particles and related maps are not associated with the processing pipeline that resulted in our 7 Å structure. Protein dynamics can lead to continuous heterogeneous populations that are very challenging to separate, resulting in poor-quality maps with no discernible structural features, which we found for our “less closed” structures. Notably, our PELDOR data, which shows a broader distribution for DSPC, agrees with our cryoEM data, because it shows the expanded state, as well as a mixture of less open structures within the ensemble. It is evident from the 2D classes that the highest-resolution particles are associated with a much thinner nanodisc, representing our expanded state. As detailed above, we attempted to resolve all states present in the data by cryoEM, but high-resolution refinement did not result in any maps with 2D structural features. While we have alternate processing pipelines that include our other states, none resulted in a final map of the expanded state at a higher resolution than the one presented at 7 Å.

Reviewer: “It is difficult to align the extended conformation with the other results and the expectations raised by the authors. For example, they introduce that the opening threshold is lowered by shorter acyl chains (Introduction lines 109-112). This is probably why the authors chose DMPC (14:0 PC) to stabilize the open state? However, no change in the structure was observed in DMPC. The structural change occurs in DSPC (18:0 PC), which has much longer acyl chains… This is a rather unexpected observation and needs some more explanation. […] Then they observe these very flat nanodiscs in DSPC, which do not match the expected increased width of a DSPC bilayer (see Figure S7). […] Do DSPC nanodiscs without MscL have the expected bilayer width or is there a problem in reconstituting these longer lipids?”

Reply: *We thank the reviewer for raising this important point. Our cryo-EM and PELDOR results consistently show the expanded structure in DSPC (as also explained in detail in the response to the previous comment). These experiments were performed using MSP1E3D1 nanodiscs, whereas the simulations reported earlier were performed only in planar bilayers, which indeed exhibit the thickness mismatch highlighted by the reviewer. To address this issue, we have now included new MD simulation data for DSPC nanodiscs in main text Fig. 5, showing a comparison between planar DSPC bilayers and MSP1E3D1 nanodiscs. The analysis includes bilayer head-to-head thickness as a function of simulation time, as well as interdigitation, calculated from the correlation fraction describing the overlap between the acyl chains of opposite leaflets. These results clearly demonstrate that the DSPC nanodisc compensates for the hydrophobic thickness mismatch by thinning the bilayer through increased interdigitation of the acyl chains, thereby providing a more favourable and stabilised membrane environment for the expanded state of EcMscL. This behaviour is also observed at high temperature (87 °C); however, the effect is considerably stronger at room temperature (30 °C), where the DSPC bilayer is in the gel phase.*

We further extended the analysis to DMPC bilayers and nanodiscs, as well as to bilayers with a native E. coli lipid composition (POPE 67%, POPG 23%, and cardiolipin 10%) containing both open and closed MscL conformations at 30 °C. The results show that DMPC nanodiscs exhibit only a slight reduction in bilayer thickness compared to the corresponding planar bilayer, with a modest increase in interdigitation that is insufficient to compensate for the thickness mismatch. Simulations in native E. coli lipid bilayers show an overall larger bilayer thickness compared to DMPC. Although the different lipid compositions do not allow a direct quantitative comparison, the interdigitation values remain negligible, suggesting that the native E. coli membrane composition predominantly favours the closed conformation of EcMscL.

Reviewer: "Similarly, the MD-simulation snapshot (Figure 4f) shows a much larger bilayer width than what is observed by cryo-EM in nanodiscs. The authors explain that the MD simulation system of the expanded state is set up using DSPC lipids in MSP1E3D1 nanodiscs (lines 759-760), but then there is no scaffolding protein visible in the system (Figure 4f), and the width of the bilayer in Figure 4f does not fit the cryo-EM structure. So, structural studies and the simulated data do not align well."

Reply: *We thank the reviewer for highlighting this point. The snapshot from MD simulations shown in Fig. 4f is taken from the late lipid bilayers simulations with DSPC lipids and this condition does not compensate for the thickness mismatch as explained in the previous comment. However, additional data has been provided now in Fig. 5 of the revised manuscript, showing the simulations of DSPC nanodiscs, indicating the thinning of the bilayer, and hence giving good agreement, aligning well with cryoEM data.*

Reviewer: "The analysis with CHAP suggests that the models in DSPC and DMPC both represent a closed conformation, as the pore has a lower solvent molecule density than bulk water. In addition, the estimated pore diameter does not fit the expected large pore of an open MscL, which was shown to be large enough to release small proteins and has a conductance of several nano-Siemens. Thus, the expanded structure is not compatible with an open MscL channel. It is also unclear whether the channel is on its way to gate or whether the expanded structure is forced by the stressed environment in the nanodisc."

Reply: *Indeed, we do not claim to have reported here the fully open EcMscL state. Instead, we conservatively assign this structure as an expanded state, as it clearly has a larger pore diameter than the closed state derived from DMPC and DOPC lipids, and is likely an intermediate EcMscL substate. Importantly, such intermediate states are key to the gating mechanism of both EcMscL (Sukharev et al. 2001) and MtMscL (Kapsalis et al. 2019; Wang et al. 2022), as well as EcMscS (Cox et al. 2013; Lane et al. 2024). These well-characterised*

states do not present full (i.e. non-selective) ion channel pore expansion, but still serve biological functions, such as adjusting their pore size and properties for ion selectivity (Cox et al. 2013; Flegler et al. 2020), and are keystone intermediates to fulfil full pore expansion. Taken together, these substates are very important to understand gating and function, not only of EcMscL, but also of other MS channels. We now make this point clear in the revised manuscript and use interchangeably the terms “expanded” and “open-like”. The CHAP results were obtained using lipid bilayer simulations, which shows a closure in DMPC bilayers while there are some events of solvation in the case of DSPC. We have now performed multiple new MD simulations of nanodiscs with DSPC. This gives the right lipid phase and compensates the bilayer thickness mismatch for the expanded EcMscL state, (please see our responses to earlier comments).

Reviewer: “MSP-nanodiscs are known to alter the structure of membrane proteins and to force them into unnatural conformations (e.g. MsbA, Hoffmann et al., Structure 2025, 2025, doi.org/10.1016/j.str.2025.02.002). This possibility should be considered as an alternative to a structure in the gating transition.”

Reply: *Please see responses above. We believe that in our case, the MSP acts as a rescue scaffold rather than a restricting and/or compressing belt, as is evident for many other membrane proteins, as suggested by the reviewer. In the rare case where the MSP belt results in some level of compression, which restricts the conformational space of ABC transporters, it is due to their unique structural architecture (i.e., MsbA), which differs dramatically compared to EcMscL. Importantly, we demonstrate that in presence of E. coli polar extract lipid and MSP1D1 nanodiscs, EcMscL adopts a predominantly closed conformation. This is borne out by new PELDOR data (Fig. 2 main text) and is quantified through local and global similarity metrics (Bhattacharyya coefficients and first Wasserstein distances respectively) in the supporting information. As we expect the EcMscL to be closed under native conditions (i.e., in the absence of tension), this demonstrates the MSP1D1 belt-protein does not induce artefactual opening. Furthermore, previous PELDOR measurements in DMPC lipids used the same belt-protein, and yielded distance distributions consistent with the closed state. However, our new MD data suggest that there is an interplay between the MSP nanodiscs and DSPC lipids. See the responses above. We now amend the text to better articulate this point.*

Reviewer: “The authors should refrain from referring to the structure of the expanded channel as “open”.”

Reply: *Please see previous responses on what one defines as “open”. EcMscL has several distinct sub-conducting states (Sukharev et al. 2001), and the pore of the expanded state solved here is compatible with the S1-S2 functionally relevant states, as supported by HOLE analysis. Nevertheless, throughout the manuscript we amend the term “open” to “expanded” or “open-like”. We hope that this will satisfy the reviewer.*

Suggested improvements

Reviewer: “Lines 100-102: It is not clear which pockets are referred to in MscL. In which respect are they similar to MscS-channels. In MscS the pockets are outside of the plane of the membrane. There is no equivalent position in MscL. Please explain what you mean for outsiders.”

Reply: *This is an excellent point, and we thank the reviewer for the opportunity to elaborate on this. Indeed, we first showed that the MscS pockets are important in channel gating (REF), and our findings were further confirmed by multiple leading groups worldwide (REF). The MscS pockets are outside of the membrane plane, but in MscL, which has a much higher gating threshold than MscS, the pockets are smaller and are situated within the lower leaflet. The relatively small size and location of MscL’s pockets mean that when lipid chain density*

decreases (because of membrane tension), it leads to only the first sub-conducting state (S1-S2), with more tension required to elicit the full opening (Kapsalis et al. 2019; Wang et al. 2022)*. We now update Fig. 4a in the revised manuscript, to highlight the pockets in both states (i.e. closed (DMPC) to the expanded (DSPC) state). This demonstrates the reduction of lipid pocket exposure to lipids in the expanded state vs the closed, consistent with the lipid-moves-first model.*

Reviewer: "Line 119-120 "75 kDa, placing it at the lower limit of current cryoEM capabilities" – is probably a bit overstated in the light of the structure of the 45 kDa NADPH oxidase (Dubach et al. 2024 NSMB). It suggests that as long as the structure is sturdy, a 75 kDa membrane protein should not be a problem for structure determination by cryo-EM. Please rephrase"

Reply: *We agree with the reviewer on this point, considering recent significant advances in resolving small proteins by cryoEM, and we have removed this sentence in the revised manuscript, and add only that it is a small membrane protein, amongst others.*

Reviewer: "Line 123 "… without the use of auxiliary binding partners" – In this context the MSP can be considered as an auxiliary binding partner, because the two rings of helices are clearly resolved in the map of MscL in DSPC. Please rephrase"

Reply: *We now write without the use of state-locking antibodies*.

Reviewer: "Lines 133-136 – please add the full names of the lipids, their chemical composition (head group, length of acyl chain, saturation) and the rationale of the choice here."

Reply: *We have now added the full names of each lipid, including head group, chain length and saturation, for their first occurrence in the manuscript, and have further expanded on our reasoning for selecting these lipids.*

Reviewer: "Lines 148-150 "minimal conformational heterogeneity" – In the CTD the side chains are not resolved, whereas they are resolved in the membrane part. This does not fit to minimal "conformational heterogeneity". Please show the variability analysis in the supplements. How was the symmetry dealt with in the variability analysis. Either explain and show or remove the statement."

Reply: *We thank the reviewer for this important comment. We agree that the original phrasing may be overstating the rigidity of the loops, given the differences in local resolution between the TMD and the CTD. The CTD exhibits reduced side-chain definition compared to the membrane region, consistent with increased local flexibility and/or particle alignment uncertainty. We have reviewed the 3D variability analysis for the closed-state structure, which was generated in three components, and two of these (components 0 and 2) show subtle movement in the transmembrane helices at the cytoplasmic side. Additionally, we observe changes to the terminal end of the helical bundle, which we attribute to flexibility in the His-tag (component 1). We have attached the three component "movies" for your review. The initial statement regarding the minimal conformational heterogeneity at the CTD was formed on the basis that the motif does not swing, and in all cases, we do not observe major lateral movements in this region. However, we agree that our description was broad and we now amend the text to reflect this.*

Reviewer: "Line 152 "only visible in the density map at a high threshold" – Should this read "lower threshold"? At "low threshold" the His-tag also becomes massive. Does the His-tag have an impact on the structure or the stability of the CTD?"

Reply: *We thank the reviewer for highlighting our error and have now corrected the manuscript to read as "lower threshold". Regarding the impact of the His-tag, while this should always be*

considered, we are confident that in the case of MscL, the C-terminal His-tag does not affect the protein's structure or function. The first MtMscL structure from Rees and co-workers (Chang et al. 1998) was solved containing a C-terminal His-tag. Additionally, C-terminal His-tagged EcMscL constructs were expressed and tested by patch-clamp electrophysiology in giant spheroplasts, and His-tagged MscL protein was purified and reconstituted into liposomes for functional assays (Mukherjee et al. 2014; Zhong et al. 2014).

Reviewer: "Figure 1: It would be helpful to add some more annotations: Add the membrane spanning width in b) (I guess this is smaller than 5.8 nm?) Similarly add the membrane spanning width in Figure 3b (I guess this fits to the labelled 2.5 nm?). 1f) the pore radius is difficult to appreciate as the Y-axis does not align with pore radius 0. Perhaps add thin/grey line for orientation at 0. It is also irritating that the distance on the Y-axis is longer than the 11.5 nm of the length of the channel shown in b). Which probe size was used in the pore analysis?"

Reply: *The Y-axis is longer than the channel length in the closed state because we have plotted the pore profile comparison of the expanded state (teal) with the closed (salmon) which is longer than open structure. However, the visualization is provided only for the expanded state. We note that CHAP defines the path length and radius profile automatically and calculates the water density along the pore coordinates as a function of time. Therefore, there is no fixed probe size.*

Reviewer: "Lines 169-170 " C-terminal end of TM1 in EcMscL is tilted by ~25° compared to the equivalent region in MtMscL" – This helix is not visible in Figure 1. Please annotate and show what you refer to."

Reply: *We thank the reviewer for bringing this to our attention. We have now added a new panel to Fig. 1e to better highlight the 25° tilt in TM1 in EcMscL when compared to MtMscL.*

Reviewer: "Lines 176-178 "The extended length of the cytoplasmic loop in EcMscL was predicted from sequence analysis, but its orientation relative to the protein was unexpected." – what was expected? Please explain in more detail."

Reply: *We now clarify this point in the revised manuscript, stating "The in silico predictions from homology modelling of the EcMscL CTD are more compact (Zhang et al., 2024; Herrera et al., 2018; Bavi et al., 2017), however from our cryo-EM data, we observe large gaps (of approximately 25 Å) separating the transmembrane and cytoplasmic domains."*

Reviewer: "Line 179 "pore radius of ~2 Å" – just to confirm… is this the radius or the diameter. It is difficult to see the distance in figure 1f and a diameter of 4 Å can be large enough to accommodate water molecules inside the pore."

Reply: *We thank the reviewer for highlighting this point. We refer to a pore radius is approximately 1-2 Å, consistent with a non-hydrated, closed pore. Indeed, TbMscL structure has a closed pore radius greater than 2 Å, and is considered non-conductive (Chang et al., 1998) We have also overlaid the HOLE analysis profiles of both TbMscL and EcMscL and find the structures to be comparable at their pore constriction points, consistent with the closed state.*

Reviewer: "Lines 192-193 "We hypothesised that MscL gating is primarily influenced by (a) lipid phase and order, and (b) nanodisc size. " – The hypothesis is not entirely clear to me. Please explain in more detail the rational why you think it is the phase and why it is the size of the nanodisc? … Or do you just want to anticipate the results?"

Response to reviewer's comments

Reply: We now revise and state that we screened multiple conditions by PELDOR, and we now include these new PELDOR measurements on native E. coli membranes (please see other responses elsewhere). The conditions showing contributions from longer distances, consistent with more open-like and expanded states present in our samples (i.e., DSPC in comparison with DMPC and native E. coli lipids), were taken forward by cryoEM, resulting in our solved structures.

Reviewer: "Lines 202-203 "Here, two distinct sites on TM2 of EcMscL (Ile79R1 and Met94R1) were individually labelled to serve as reporters for detecting channel expansion" – Please show the labelling efficiency of the sites. Do you expect that all 5 sites of a channel are labelled. How does the labelling alter the structure The spin-labels appear to be very bulky and the channels are very small. Therefore, the question arises whether the labelled channel in EPR and the unlabelled channel in cryo-EM represent the same structure. This is important as the calculated and measured distances do not match(Figure 2). Perhaps check the structure of the labelled MscL by cryo-EM. (This need to be in 3D and resolve the helices… 6-7 Å resolution should be sufficient)."

Reply: *We now include new PELDOR measurements, performed both in DDM detergent solution, and under native E. coli lipid conditions for both the I79R1 and M94R1 constructs, and measured technical replicates to substantially higher signal-to-noise (SNR) than before. We note that the extinction coefficient of EcMscL is extremely low (i.e. Trp- and Tyr-free native construct), and so quantification of the protein concentration is non-trivial. We have bypassed this issue through analysis of PELDOR modulation depths to assign labelling efficiencies of 64.5 ± 2.4 % and 79.7 ± 2.4 %, for I79R1 and M94R1, respectively. This analysis has now been added to the Supporting Information. Global fitting of technical replicates we have also now implemented provides more robust fitting and processing. As per our previous response, the small MTSSL label does not disrupt EcMscL, neither functionally nor structurally and particularly on those two sites we purposedly selected based on previous literature studies and screens of functional characterisation of Cys- modified EcMscL (see relevant response above). We now find excellent agreement between experimental and modelled PELDOR distances, using our cryoEM structures and refine the revised manuscript text to reflect these points.*

Regarding the acquisition of spin-labelled structures, although we appreciate the suggestion, this is out of the scope of this study for several reasons (see also relevant response above): (i) as the reviewer pointed out, "the low resolution does not permit the identification of side chains. Therefore, deviations in the rotation around helical axes or small shifts by ½ turn along the helical axes are possible". Thus, this will introduce new uncertainties between the EM and modelling PELDOR distances.
(ii) We will need to obtain resolution better than 3 Å to see density for individual spin labels. We previously reported such a structure for the MscS channel and noticed ~Å agreement between PELDOR and X-ray structure distances. Given that our closed structure of EcMscL took more than 41 years to be reported/solved, and at best the resolution achieved was ~4 Å in DMPC lipids, it would not be possible and is clearly out of the scope to pursue this in the current study. (iii) Even if we solve a high-resolution cryoEM structure, this does not warrant capturing the entire ensemble, as is the case of the expanded state, which the reviewer rightly also highlighted.

Reviewer: "Lines 218-219 "For comparison, previous measurements of Met94R1 EcMscL in DMPC, the same lipid in which we resolved the EcMscL closed state, showed a more homogenous distribution, in a more compact configuration"- In DMPC (Figure 2C) there are three distances. None seems to match the closed or the expanded structure observed in cryo-EM. Please explain."

Reply: *We acknowledge the reviewer's comment. As we have now recorded multiple new PELDOR measurements for both mutants in DDM and E. coli polar extract lipids, we remove the previously reported data in MSP1D1 nanodiscs with DMPC lipids and instead refer to it in the discussion. This decision is partly motivated as this condition was only recorded previously for M94R1, and not I79R1, and used a different, and less sensitive, EPR instrument than measurements in the current study. Importantly, the new PELDOR data agrees strongly with predicted distance distributions from the cryo-EM structures solved in this work. We also see a consistent trend in similarity metrics when transitioning from E. coli polar extract lipids to DSPC lipids. As a note, long distance features (i.e., corresponding to low-frequency oscillations) can arise as artefacts in PELDOR measurements, particularly in the red region of the reliability colour bar. This arises from the limited dipolar evolution time of the system, imposed by the phase memory time, and this is widely documented in literature.*

Reviewer: "Lines 251-252 "This state is characterised by a significant reduction in TMD thickness and a wider pore entrance" … The wider pore entrance shown in Figure 3F seems to sample different regions. It looks much more narrow in 3b/3a. than in 3f. Please make a better presentation of the pore at the narrowest site. For example, use a slice in Van der Walls representation at the narrowest site viewed perpendicular to the symmetry axis and also parallel to the symmetry axis. Make sure that side chain density is present in this representation. Otherwise, the pore diameter is overestimated in the Can der Walls presentation. There is also a discrepancy between the labelling in 3f (4 Å and 8 Å) vs 2 Å and 8 Å in the figure legend. Probably radius and diameter got mixed up. Please fix."

Reply: *In Fig. 3f, to highlight the differences in pore size between the closed and expanded states, only the TMD (residues 1-104) is shown. The pore appears wider in Fig 3f, compared to Fig. 3a-b, because the cytoplasmic bundle (with pore diameter ~3 Å), which does not expand or change conformation upon expansion of the TMD, is not shown, giving the top-down view a narrower appearance. While we point out in the figure legend that only residues 1-104 are shown in Fig. 3f, we agree that this could be clearer and have now amended the figure legend to explicitly highlight that residues 105-136 (encompassing the cytoplasmic loops and CTD), were removed to better visualise the pore region only. Thank you to the reviewer for pointing out our error in reporting the diameter of each channel in Fig. 3f, we have now corrected these to show the pore diameter only (~2 Å and ~8 Å).*

Reviewer: "Lines 279-281 "In contrast to earlier models proposing its dissociation and lateral repositioning to the membrane plane, the CTD appears to remain intact" …. But then the expanded conformation does not show a pore opening that would fit to 3-4 nS conductance. Therefore, the structure does not tell what happens when the channel opens properly. Please rephrase."

Reply: We acknowledge the reviewer comment, and we now amend the text to reflect that the helical bundle of the CTD remains associated, at least up to the expanded state presented in this study.

"Line 315-315 "Although DSPC lipids form thicker bilayers than DMPC (3.6 nm vs 2.5 nm)," – The thicker bilayer does not seem to match with what is observed in the cryo-EM structure in 3a/3b. Please explain. There are also later referrals to the thicker bilayer (e.g. line 326). It would be helpful if this apparent contradiction between the expected thicker bilayer and the thinner structure would be explained."

Reply: *We thank the reviewer for pointing out this discrepancy. We have now included the new data for DSPC and DMPC in nanodiscs, which shows thinning of the bilayer with DSPC lipids in nanodiscs compensating the thickness mismatch. The detailed response is provided in the earlier comments.*

Response to reviewer's comments

"Figure 4 – solvent density in b-d suggest that all three channels are closed or de-wetted. It would help to have the energy plot from CHAP to show that water can freely pass the pores in DSPC. Figure legend: Please explain what the "blue balls" are. If this is water, why are these water molecules shown and others are not shown. At which time points are the snapshots taken. The DSPC snapshot in f looks less flat than the cryo-EM structure. Did they change or is it an optical illusion? Please add more information to the figure legend and compare the MD snapshot with the starting model."

Reply: *The water density calculated from CHAP represented in Fig. 4b-d informs about the total closure of the pore when closed state is simulated with DMPC lipids. While there is very small number of solvation events when closed states are modelled in DSPC lipids, and solvation events occur more frequently for the expanded state, when modelled in DSPC bilayers. Overall, we agree with the reviewer that the channels are mostly closed, even in DSPC. However, our new data shows that DSPC favours expanded state structures in nanodiscs, compared to bilayers in MD simulations. For details see the responses to the earlier comments. The energy plots from CHAP analysis are now included in the supporting information, revealing favourable energy contributions in the pore region for the expanded state in DSPC, as compared to closed state in DMPC.*

"Discussion – Do not refer to the expanded structure as "open". This is misleading as the solvent density in the simulations does not support free passage of water. It is not even clear that the cryo-EM structure in DSPC is a structure that is relevant to gating. You might want to add some experimental evidence, which shows that the channel becomes conductive in DSPC at lower temperatures to support your claim."

Reply: *This is an important point. We refer to this state (please see also our previous responses) as open when compared to the closed state (i.e., DMPC and DOPC conditions). However, we understand the point raised by the reviewer and now refer to this state in the revised manuscript, as an expanded state resembling open-like EcMscL to align with MscL's previously reported sub-conducting states. Regarding the passage of water, the initial structure becomes compressed by the bilayer following equilibration during the MD simulations. The MD simulation begins from the "solved" expanded state, which has a well-hydrated pore, and fluctuates between expanded and closed, throughout the rest of the trajectory. To clarify this, we now present HOLE analysis for water to pass through, as well as CHAP analysis – in the revised manuscript – which shows that the pore stays open and hydrated for the first ~20 ns before bilayer compression. Indeed, the structure is mostly closed in the flat bilayer simulation as shown by the compression and pore analysis, while the new data with nanodiscs exhibit the opening with higher probability as compared to the earlier flat bilayer simulations. We now clarify these points in the revised manuscript. We have implemented the reviewer suggestion. Indeed, the new data included in the manuscript consisting of DSPC lipids in nanodiscs shows that this phase is more favourable for open structure as discussed in the earlier responses. However, nanodisc don't support CHAP calculations. Hence, we have provided the water number density calculation in the pore region and gating filter which show a clear and significant enhancement for expanded state in DSPC nanodiscs with time as compared to closed state in DMPC nanodiscs which stay closed throughout the MD simulation. The data is shown in the Supporting Information.*

"Lines 418-420 "… speculate… more lipid depletion from the nanodiscs" – This is not entirely clear. Is there a phase transition after the reconstitution into nanodiscs? All components are packaged simultaneously. Proteins and lipids should occupy the available space within the nanodisc. At the end of the reconstitution the nanodiscs should just package more or less lipids to form the most stable system rather than a tensed system. Why should there be more tension? In addition, the surface area is not that tightly fixed in a nanodisc as often seen in class averages of other membrane proteins."

Response to reviewer's comments

Reply: *As raised by reviewer 4, we now consider the role of interdigitation and thickness reduction as important factors for this transition in nanodiscs. Please see relevant responses to other comments throughout. In brief, new data consisting of nanodiscs containing DSPC exhibit an interdigitated phase of the lipid bilayer in MD simulations leading to thinning the membrane, which might bring tight packing (and subsequent surface tension) due to interdigitation, constrained also by the belt-protein, which is not seen in lipid bilayers. We have now corrected our discussion and hypothesis and have replaced Fig. 5 with the interdigitation and thickness comparison between the lipid bilayers and nanodiscs simulations.*

"Lines 434 " … although no lipids were resolved" The maps of the closed channel in DMPC is really nice and shows clear side chain density. There is no reason to believe that the map quality is not sufficient to resolve lipids. Therefore, the absence of lipids in the closed structure suggests that there are no tightly bound lipids. They rather seem to exchange and have some mobility as they are not resolved. Therefore, a model in which gating is initiated by lipids being pulled out of the pockets is not convincing based on the represented structures. Perhaps as no lipids are resolved, this is not the study that enlightens the role of lipids for the gating mechanism and should be discussed elsewhere in a different context?"

Reply: *At the resolution we obtained (4 Å), we do not resolve lipids, owing to their inherent flexibility and mobility. Regarding the mapping to the pockets and their role in gating, see response above, and additional points in the Supporting Information and revised manuscript.*

""fewer lipids in the expanded state" – This is not visible in 4e/f please make clear which lipids in which pockets you refer to and show how the pocket size changes."

Reply: *These residues are now included in our histograms in the Supplementary Information. We have now calculated the lipid contacts in the pockets between the expanded and closed state, and a correspond heat contour plot overlaid on the EcMscL structure.*

"Figure 5b – Gating mechanism – Please add more information to the figure legend. What is the mechanism? The channel expands but the scheme does not end with an open pore of the expected size for large conductance. How do you imagine that this leads to an open pore with 2-3 nm diameter. Otherwise, this model probably does not really explain the gating mechanism and it is somewhat overstated"

Reply: *On the point regarding factor that contribute to gating, we cannot say definitively which factor contributes more/most to the gating of EcMscL, as they are interlinked. We here are trying to produce conditions that stabilise expanded or open-like states for structural analysis etc. Now we write a model for "initial channel expansion". We have also indicated in main text Fig. 5 of the revised manuscript, the comparison of thickness and interdigitation in DSPC bilayers and the new MD simulations of DSPC nanodiscs, as discussed in earlier comments. The reduction in thickness and enhanced interdigitation is the favourable phase to accommodate the channel expansion and is seen only in DSPC nanodiscs.*

"Line 476 "upward shift" – avoid phrases like "upward" and "downward". This is confusing as there is no absolute orientation. Refer to "cytosolic" and "periplasmic" side or, towards the plane of membrane or away from the plane of membrane"

Reply: *We thank the reviewer for bringing this to our attention and have now revised the manuscript main text to describe the movement of the CTD towards the cytoplasmic side of the channel.*

"Line 48ß-481 "partial helix" – annotate the partial helix and the three residues in Figure 1 and refer to it […]"

Response to reviewer's comments

Reply: *We have now added a new panel, Fig. 1d, to show the partial helical structure in more detail, including the side chain positions of the three residues that form the motif.*

"Add data availability statement"

Reply: *Thank you for pointing this out. We have now added this statement to the main text, per the reviewer suggestion.*

"MscL Purification 535-538 – show the outcome of the purification in the SI (SEC, SDS page of fractions, which fractions have been used for analysis), same for EPR labelling experiments. Show labelling efficiency. This is really an absolutely necessary part to understand what the properties of the purified MscL were."

"Reply: *We have now included SDS-PAGE gels and SEC analysis for the EcMscL purification in our Supplementary Information. We have now calculated spin labelling efficiencies and made new sample preparations as well as technical replicates for both I79R1 and M94R1 mutants. We now include this information in the Supplementary Information of the revised manuscript. See our comments regarding spin labelling below.*

"Nanodisc (lines 574-588) – What is the starting material for MscL. Did you use the sample from the SEC if so, show which fractions of the SEC were reconstituted."

Reply: *Please refer to our responses to previous comments. Following immobilized metal affinity chromatography (IMAC) purification and size exclusion chromatography (SEC), we obtained highly purified EcMscL. Fractions corresponding to the main SEC peak were analysed by SDS-PAGE, then concentrated and stored at -80 ℃, prior to reconstitution or labelling.*

"EPR sample preparation (589-597) This involved freezing of 65 µl with glycerol. During this step every lipid sample should transform into a gel phase. So, all EPR measurements should be in gel phase. How would this effect the expected outcome/distances, when the hypothesis of the phase transition for opening is correct?"

Reply: *While we appreciate the reviewer's comment, it is important to note that it assumes both that: (i) the timescale of the phase transition of the lipids is fast with respect to the rate of freezing, and (ii) the kinetics of channel expansion/opening are also fast with respect to the rate of freezing (i.e., the transition happens instantaneously). Literature on the kinetics of the closed-S1 transition suggests a timescale of high ms, thus it is more likely the gel-phase transition occurs significantly faster than initial channel expansion/opening. As such, PELDOR distance distributions should reflect a heterogeneous mixture of states (at various stages of hysteresis after the gel-phase transition, depending on the kinetics underlying the specific conformational transitions). Indeed, this is what we observe experimentally by PELDOR, rather than a discrete, monodisperse expanded state.*

Based on the analysis of the new MD data consisting of DSPC nanodiscs, both at 30 °C and 87 °C (thickness, interdigitation, pore analysis and solvation), we can now conclude that channel expansion does not heavily depend on the lipid transition temperature. The interdigitation and the pore radius is even higher under high temperature conditions.

"Lines 650-651 – Are these the two access codes of the structures determined in this study – please add in the data availability statement or give an appropriate reference."

Response to reviewer's comments

Reply: *Yes, the PDB codes listed in the manuscript are those generated for our new structures. We have amended the text to make this clearer and have also included the codes in our data availability statement.*

"Line 661 "10 eV Selectris energy filter" . it is a slit width of 10 eV – not a 10eV filter. Rephrase"

Reply: *Thank you, we have now corrected this statement in the methods section.*

"Line 708, 712 "at 300 Å/pixel" – this must be wrong. Do you mean 300 Å width of the box?"

Reply: *Thank you for bringing this to our attention. The box sizes are now reported in Å.*

"Lines 758-760 … built … in nanodiscs" – is "nanodisc" true as the scaffolding protein is nowhere visible in the MD simulation snapshot in Figure 4"

Reply: *Thank you for spotting this mistake. Our MD simulations were indeed run in lipid bilayers. We have now corrected this in the manuscript.*

"Lines 840-841 – please show the position of the deprotected residues in the structure of the closed and expanded state. Is this exposed in the periplasmic cavity?"
Reply: *We have now added new panels, Supplementary Fig. 4b,c, to highlight the deprotected residues in the closed and expanded structures. We appreciate the opportunity to show that the deprotected residues are more exposed to solvent in the periplasmic cavity in the expanded state structure.*

"Lines 853-860 CHAP annotation – show delta G plots instead or together with the solvent density"

Reply: *Thank you to the reviewer for the suggestion, we have now calculated the delta G plots from CHAP, but observed that the errors associated with these values were on the order of 10^{38} kT, particularly at the pore region, therefore we cannot discriminate differences between expanded and closed-state(s) based on these values.*

"EM workflows – indicate where C5 symmetry was applied. For example, in the ab-initio in S9 C5 was applied and in the ab-initios of the others not. The application of the C5 in the ab-initio gives rise to the funny structure in which the symmetry axis was aligned wrongly."

Reply: *We have now included information regarding the application of either C1 and C5 symmetry for each refinement, for all our EM workflows, within the figure itself and in the figure legend.*

"Figure S10 / lines 954 and 958 "300 Å/pixel" – please correct"

Reply: *Thank you. Please refer to our response above regarding these corrections.*

Reviewer #2 (Remarks to the Author):

"In this manuscript, Hardman and colleagues report the first full-length cryo-EM structures of the E. coli mechanosensitive channel MscL in both closed and expanded conformations, reconstituted in lipid nanodiscs. By combining cryo-EM with PELDOR spectroscopy, HDX-MS and atomistic MD simulations, the authors identify lipid conditions (DSPC nanodiscs) that stabilize an expanded, open-like state and characterize the associated structural rearrangements. Their data support a helix-tilt gating mechanism in which TM1 and TM2

undergo substantial radial expansion and tilting, while the C-terminal domain remains assembled as a pentamer and shifts slightly without dissociation. The integrative approach further highlights changes in protein–lipid interactions and solvent accessibility that accompany channel expansion, providing a structural framework for understanding lipid-driven mechanosensitive gating in EcMscL.
This is altogether excellent work and a very nice example of integrated structural biology. I am not in the mechanosensitive channel field and honestly thought this would have been an "easy enough" target for cryo-EM — clearly, I was naïve. Congratulations to the authors for solving this long-standing structural enigma. The lipid-dependent stabilization of distinct conformations is particularly elegant."

Reply: *We are pleased that the reviewer acknowledges the challenges we faced to solve this long-standing fundamental question and advances to the field through this landmark and highly anticipated protein structure in distinct functionally relevant states.*

"My main question here is more out of curiosity than as a real reservation, and concerns the choice of lipids themselves. None of them are found in the E. coli membrane lipid composition. Did the authors screen other lipid compositions, including E. coli-like ones? If yes, can they share what they observed? I understand that, for a mechanosensitive channel, the physical properties of the membrane used here were likely key to stabilizing and resolving the observed conformations. How do the authors think this opening occurs in the cell, where the lipid composition is much more complex and the membrane is crowded with other proteins? I would appreciate some speculation on this in the discussion."

Reply: *We thank the reviewer for their comment and suggestions. We have now performed new PELDOR measurements (at high SNR) in nanodiscs containing native E. coli lipids to study the gating of the structure of EcMscL under native lipid conditions. We have performed these for both labelling sites (i.e., I79R1 and M94R1). We have also set up independent MD simulations with E. coli membrane composition to further investigate the gating and dynamics. We find that the distances observed by PELDOR for both sites using E. coli polar extract lipids are very similar to those observed using either DMPC or DOPC, reflecting the closed-state conformation of EcMscL suggesting that the nanodisc scaffold or belt-protein is not a determining factor inducing the conformational state of EcMscL*

"[…]Besides this, I only have minor comments.
L122. The authors rightfully highlight that the structures were obtained without the use of fiducials, nanobodies or scaffolds. What was the key factor that enabled structure determination in this case? What prevented EcMscL from being solved earlier? Was the use of nanodiscs decisive? Could the authors elaborate on what made the difference here compared to previous attempts?"

Reply: *We thank the reviewer for their comment. We now elaborate and suggest possible scenarios based on scientific evidence from existing literature and our own data presented here, obtained from our multidisciplinary approach. See also our related response elsewhere. One such possibility is that the belt protein could have acted as a scaffold to enable trapping the EcMscL expanded state. Indeed, in our density map of EcMscL in MSP1D1/DSPC lipids, the outermost regions of the periplasmic side of TM1/TM2 are close to or touching the scaffold protein. While these interactions may have facilitated the stabilisation of the expanded state for structural determination, they are unlikely to be a key contributor to channel expansion, because the same MSP1D1 scaffold was used with both DMPC and DOPC lipids, for which only the closed-state conformation was observed. Previous attempts to determine the cryoEM structure of EcMscL (Cox et al. 2021) used the larger MSP1E3D1 scaffold with DOPC. In this study, the authors resolved 2D class averages of EcMscL in what resembles the closed state conformation determined here, and used beta-cyclodextrin (βCD) to extract lipids from the nanodiscs to induce an alternate conformation that broadly resembles our expanded structure,*

with significant flattening of the TMD (see Fig. below for comparison). In any case, this study did not lead to a high-resolution three-dimensional structure of the EcMscL protein using MSP1E3D1 and DOPC (Cox et al. 2021). We now also include a new figure panel in the main text, showing density of the belt-protein, resolved by cryo-EM in both expanded- and closed-state structures.

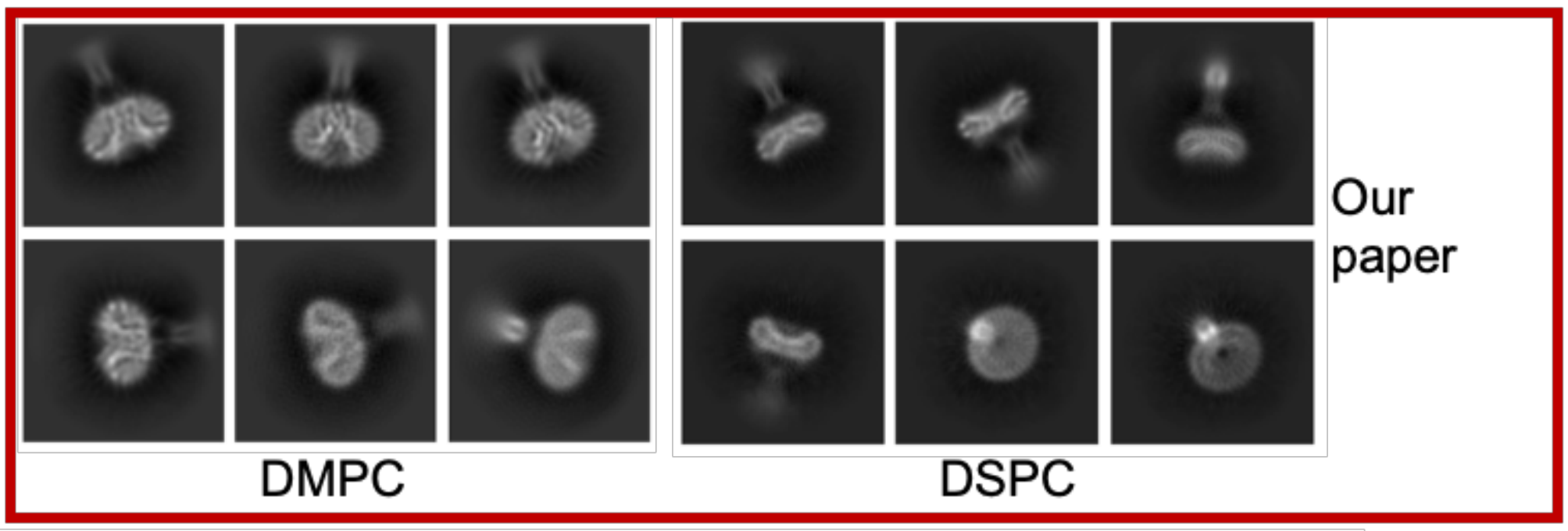

[editorial note: third party material redacted]

"L147. "The ten-amino-acid loop connecting the C-terminus of TM2 to the CTD is proline-rich (Pro109, Pro113, Pro115), reinforcing its stability." The statement that the proline-rich loop "reinforces its stability" is somewhat ambiguous. As prolines are typically helix-disrupting residues, could the authors clarify whether they mean reduced conformational flexibility or stabilization of a specific loop geometry?"

Reply: We thank the reviewer for their comment and suggestion. Indeed, in both the 2D class averages and maps of the protein in the closed and expanded state, the CTD is clearly positioned well below the TMD. Our 3D variability analysis further reveals only moderate shifts in the position of the CTD, indicating that the cytoplasmic loops connecting these domains are relatively rigid. We propose that this rigidity arises from the loop sequence itself, which contains three proline residues (Pro109, Pro113 and Pro115) interspersed with alanine residues. The distinctive cyclic structure of proline strongly constrains the backbone dihedral angles (by fixing the phi angle at ~65°), thereby substantially reducing conformational flexibility (Lassak et al. 2026). Although the density in this region is insufficient to define a precise geometric configuration for the loops, the trajectory of the connecting density can still be followed at high map thresholds. These observations suggest that the loops are comparatively rigid, consistent with the generally reduced flexibility conferred by proline-rich sequences relative to other amino acid compositions.

"L225. The authors state that PELDOR identified DSPC-MSP1D1 nanodiscs as favouring opening of MscL. I must admit that I do not immediately see this clearly in the PELDOR data (fig2b and c). In DMPC, the measured distances did not match the modelling of the closed state; therefore, what was the rationale for concluding that DSPC was the most promising condition for cryo-EM based on the PELDOR data? The manuscript mentions an EPR-based screen — did the authors test additional lipid compositions and/or nanodisc sizes beyond those shown? Why was DSPC selected specifically?"

Reply: *We thank the reviewer for raising this point. Please see also previous responses to similar points throughout. We used PELDOR to screen several conditions as it offers high resolution for detecting such conformational transitions, is substantially faster than cryoEM, and importantly, informs on the conformational ensemble, which was a key objective here. Using PELDOR, we observed that under DSPC conditions for each mutant the distance distributions consistently deviated more from the closed state than in E. coli polar extract lipid in MSP1D1 nanodiscs. This is an important control, as under native conditions we expect EcMscL to adopt a closed conformation. Notably, because the transition from the closed state*

to the S1 sub-conducting state is rate limiting, with transitions from S1-S4 progressing more quickly, this precludes kinetic trapping of the S1/S2 sub-conducting state(s) (which our “expanded state” likely corresponds to) by EPR. Therefore, we monitor channel expansion by deviation from the closed state, rather than better agreement to the expanded state. We have performed a quantitative analysis of the PELDOR distance distributions (by calculating both local and global similarity metrics) and processed data in two distinct analysis pipelines for robustness. We consistently observe that DSPC causes a conformational shift away from the closed state, regardless of nanodisc, compared to MSP1D1 nanodiscs with E. coli lipids. This analysis is now included in the supporting information. Thus, PELDOR was instrumental in guiding these decisions, ultimately leading to the determination of the expanded-state structure. In addition, previously published data validated that DMPC and DOPC favour more closed conformations, in agreement with the cryo-EM observations. As the reviewer correctly noted, there was no major difference between the two DSPC conditions tested. We therefore chose to proceed with the slightly smaller nanodisc MSP1D1, partly because Cox et al., (2021) had used MSP1D1E3 nanodiscs with DOPC lipids. Also, we can exclude the role of the MSP1D1 belt-protein inducing artefactual opening – validated by the E. coli lipid condition in the same nanodisc consistently showing a predominantly closed conformation – but have not shown this in the E3D1 nanodisc. We therefore concluded that these conditions were more likely to facilitate the acquisition of open-like structures by cryoEM. We have now amended the text in the revised manuscript according to better reflect these points.

“Another question related to the use of PELDOR on a pentameric channel. It is assumed that the changes are symmetric and coordinated? Is this the case? Do the authors observe asymmetry between the monomers in specific cryo EM subclasses?”

Reply: *Thank you for allowing us to clarify this point. When processing our data for cryoEM, symmetry is not imposed during the earlier stages of refinement (i.e., ab-initio reconstruction and heterogeneous refinement), but C5 symmetry is applied during high-resolution refinement (in our case non-uniform refinement). Based on analysis of our initial ab-initio models, we see minimal differences between the five subunits that form the channel, however, since at this resolution we cannot resolve side-chain density, we cannot comment on the specific asymmetries of the side chain positions between the chains. Regarding the PELDOR analyses, the distance distributions are fitted non-parametrically (by solving the moderately ill-posed problem via Tikhonov regularisation), and so does not make explicit assumptions about concerted motions or the symmetry of the system.*

"L286. The HDX-MS data are mapped onto the structure, but I find the visualization somewhat difficult to interpret. Could the authors present the HDX differences on a representation similar to Fig. 3f, with helices clearly visible, to facilitate interpretation?"

Reply: *Fig. 3h, representing HDX-MS of EcMscL in DMPC and DSPC, has now been amended in the revised manuscript to show the deprotected residues without the surface view on, like how the protein is represented in Fig. 3e,f.*

Reviewer #3 (Remarks to the Author):

"The translation of mechanical force into intracellular signals (mechanotransduction) is a crucial process present across living organisms. The MscL from E. coli (EcMscL) is a paradigm example, and here the authors solved full-length structures of EcMscL for the first time. Using cryoEM, they observed both the closed and expanded structures in DMPC/DOPC and DSPC lipid nanodiscs. Further, by combining PELDOR spectroscopy, hydrogen-deuterium exchange mass spectrometry, and molecular dynamics simulations, the authors identified sites relevant for the closed-open transition. Overall, the manuscript presents novel information on the structural and mechanistic aspects of mechanotransduction and further advances the field."

Reply: *We appreciate the reviewer for recognising our efforts and multidisciplinary approach to advance the fundamental field of ion channel mechanical sensing.*

Comments

"Please state the overall resolution for the solved structures wherever relevant for clarity."

Reply: *We thank the reviewer for highlighting this oversight and have now added the overall resolution for all structures throughout the text.*

"As the channel forms a homopentamer, the modulation depth of the PELDOR data must be very large, which is not the case for the presented data sets (Figure S3: modulation depth < 10% relative to a much higher expected value). Please comment on the explanations for that, including reporting the spin labelling efficiency for each variant."

Reply: *We thank the reviewer for highlighting this apparent discrepancy. However, we point out that the data shown have a reduced apparent modulation depth because power-scaling (for 5-spins) is applied within the ComparativeDeerAnalyzer (CDA) 2.0. Therefore, only the spin-pair contribution to the modulation depth is accounted for, while the unscaled PELDOR data has higher modulation depths (than ~10% as stated by the reviewer) for all tested conditions. We have now performed biological replicates of most conditions, and observe consistent modulation depths, except for I79R1 in MSPD1E3 nanodiscs and DSPC lipids, where we attribute the previously low modulation depth (~30%) to poor reconstitution efficiency and the possibility of EcMscL monomerization. Minor contribution from monomeric MscL (owing to broad, overlapping peaks in the size exclusion chromatography (SEC) profile, corresponding nominally to monomeric and pentameric MscL) is a probable explanation for the slight deviation in modulation depths (i.e., as compared to a quantitatively spin-labelled pentamer). To test this, we measured a PELDOR sample in DDM taken from a SEC profile peak nominally corresponding to monomeric MscL, and while we observed a significant reduction in modulation depth, the modulation depth was non-zero, suggesting monomeric and pentameric MscL cannot be quantitatively separated (see below). We have now also performed PELDOR measurements in DDM detergent and in E. coli polar extract lipid in MSP1D1 nanodiscs.*

Regarding labelling efficiency, CW-EPR quantification of EcMscL protein concentration is unreliable in this system due to the low molar extinction coefficient ($\varepsilon = 1490\ M^{-1}cm^{-1}$),

preventing accurate concentration determination. We therefore use modulation depths from PELDOR experiments as an indirect measure to constrain both labelling efficiency and oligomeric distribution. EcMscL exists in equilibrium between monomeric and pentameric states. The observed modulation depth (Δ) can then be described as a weighted mixture (Ackermann et al., 2017; Ackermann et al., 2023):

$$\Delta = 1 - \frac{\sum_{k=1}^{n} \binom{n}{k} f^{k}(1-f)^{n-k}(1-\lambda)^{k-1} k\, x(k)}{\sum_{k=1}^{n} \binom{n}{k} f^{k}(1-f)^{n-k} k\, x(k)} \quad (1)$$

Where: n is the total number of labelling sites (i.e., here, each monomer contains a single cysteine residue), k is the number of distinct states containing k spins, f is the labelling efficiency, λ is the inversion efficiency at the pump frequency, and $x(k)$ is the fractional population of oligomeric species containing k spins. For nitroxide spin labels at Q-band frequencies and using rectangular microwave pulses, λ is typically 0.25–0.35. Therefore, we use the conservative value of 0.25. For a fully labelled pentamer (n = 5), the maximum observable modulation depth (Δ_{max}) is defined as:

$$\Delta_{max} = 1 - (1-\lambda)^{n-1} \quad (2)$$

This defines the upper physical limit for Δ under experimental conditions, assuming quantitative labelling efficiency (f = 1) and pentamer fraction (x_5 =1). The SEC profiles indicated two peaks, likely corresponding to a pentamer-enriched fraction (peak 1) and a monomer-enriched fraction (peak 2) for both I79R1 and M94R1. Though it is understood that the profile is not quantitatively symmetric, we assume that the pentamer and monomer fractions are roughly inverted across peaks 1 and 2. We measured PELDOR and observed Δ_{peak1} of 0.496 and 0.476 for I79R1, in detergent and after reconstitution into DSPC MSP1D1 nanodiscs, respectively. Similarly, we observed Δ_{peak1} of 0.562 and 0.563 for M94R1, in both detergent and after reconstitution into DSPC MSP1D1 nanodiscs, respectively. We estimated a modulation depth error of 2-4%, corresponding to ±0.01-0.02, consistent with previous literature (Wort et al., 2019; Wort et al., 2021; Ackermann et al., 2021; Ackermann et al., 2022). Global fitting and subsequent bootstrapping (over 500 trials) of the experimental modulation depths to estimate parameters x_5, f_{I79R1}, and f_{M94R1} yielded 2σ confidence intervals: x_5 = 0.794 ± 0.020 , f_{I79R1} = 0.645 ± 0.024, and f_{M94R1} = 0.797 ± 0.024. As an independent validation, we subsequently measured PELDOR of I79R1 and observed Δ_{peak2} of 0.285 (see above), in excellent agreement with the predicted value of 0.288 (assuming x_5 = 0.206, f_{I79R1} = 0.645, and λ = 0.25).

We thank the reviewer for requesting the labelling efficiencies and directing us towards this analysis. We have now added this into the supplementary materials. Regarding the use of DSPC instead of E. coli polar extract for cryo-EM, our reasoning was two-fold: (i) the resting-state of EcMscL in the native membrane environment is the closed state, and (ii) use of a uniform lipid profile would allow unambiguous assignment of lipid densities in the cryo-EM structures. We also note that, as expected, in presence of E.coli polar extract lipid the EcMscL channel is predominantly in the closed state (see discussion of similarity metrics below). This implies that the MSP1D1 nanodisc does not cause artefactual expansion of the EcMscL channel and motivated us to pursue cryo-EM in this nanodisc. While we observe similar conformational heterogeneity in DSPC lipid (regardless of nanodisc), we cannot exclude that the MSP1E3D1 nanodisc causes artefactual expansion of the EcMscL channel, as we have not run the corresponding native lipid control condition.

"Displaying PELDOR-derived distributions exclusively from DEERNet analysis is not appropriate (despite the given justification for that). The corresponding consensus distribution must be shown at least in the supporting information (with appropriate discussion) for the readers."

Reply: *We thank the reviewer for this comment and agree that the consensus distribution should be shown. We have now added consensus distributions (and time-domain data) for technical replicates to the supplementary material. As a note, we have observed that when relying on power-scaling inside the CDA, fit quality for the consensus fit to time-domain data (i.e., corresponding to the consensus distributions) is sometimes poor. This is even the case when the fit quality by both DeerLab and DeerNet individually is very high. We now provide a rigorous analysis using both DeerLab global fitting and with compactness regularisation (shown in the main text) and the consensus distributions (shown in the Supporting Information). Furthermore, we have moved the MSP1D1E3 DSPC nanodisc data to the supplementary material, instead focussing on comparison of MSP1D1 nanodiscs either in DSPC or E. coli polar extract lipids (see below).*

"Figure 2b-c: The conclusion that the smaller MSP1D1 scaffold shifted the conformational equilibrium toward more open or expanded states, compared to the larger nanodisc (MSP1E3D1), is not evident. Considering that the distributions are predicted from neural network (DEERNet) analysis and are equally broad, a meaningful interpretation suggests a rather similar heterogeneous distribution covering multiple states (as the authors themselves stated afterwards)."

Reply: *Excellent point. We agree with the reviewer that we should further emphasise the heterogeneity present under both conditions. It is important to appreciate that our expanded-state structure has a pore diameter corresponding to an S1-S2 sub-conducting state (Sukharev et al. 1999), whereby the longer distances may correspond to other sub-conducting states with larger pore diameters, consistent with a highly heterogeneous population. Regarding the claim that MSP1D1 nanodiscs shift the conformational equilibria toward a more open/expanded state; to test this, we now calculated the Bhattacharyya coefficients and first Wasserstein distances, as local and global similarity metrics, respectively. We reasoned that a shift to more open states should result in concomitant reduced overlap with the in silico closed state model distribution and increased overlap with the in silico expanded state model distribution. However, because the transition from S1-S4 sub-conducting states progresses on the ms timescale, flash freezing in $N_{2(l)}$ is too slow to kinetically trap the S1 sub-conducting state by EPR. This not only explains the heterogeneity that we observe, but also why the DSPC lipid conditions do not show consistently higher overlap with the in silico expanded state model distribution (i.e., the population is likely comprised of closed, S1, S2, S3, and S4 EcMscL conformations, rather than simply closed, and S1). Instead, we observe consistently reduced overlap with the in silico closed state model distribution for DSPC lipid PELDOR distributions, compared to the E. coli polar extract lipid (i.e., native lipid environment where we expect the channel to be predominantly in a closed conformation). Furthermore, we see this trend for both metrics, and regardless of whether we take the DeerLab (with global fitting of technical replicates and compactness regularisation) or the CDA (i.e., consensus) processed distance distributions. This suggests that DSPC shifts the EcMscL conformational ensemble towards expanded states, regardless of nanodisc size. Please see our previous responses on similar points. We now rephrase and elaborate these points in the main text to reflect this in the revised manuscript.*

"Figure 4b-d, please define/describe the y-axis parameter in the legend."

Reply: *We thank the reviewer for pointing this out. The y-axis label is changed to "channel coordinates" in the figure.*

"Line 375, 'By contrast, in DSPC bilayers at 87 ℃…' this needs more clarity in the main text. Why 87 ℃ and what happens at lower temperatures (the stability of the protein and the expanded structure)?"

Reply: *Thank you. The protein has not experienced 87 ℃. In MD the high temperature was applied only to change lipid phase – well above Tm/below boiling and increases dynamics to reduce computational time and access longer timescales. We acknowledge that the temperature is not physiologically relevant, we only use this temperature in MD because it is well above the transition temperature, and it can accelerate the simulations.*

"Line 378-381: 'Together, these results indicate that temperature and the associated changes in lipid phase behaviour (Supplementary Fig. 7) strongly influence EcMscL hydration in DSPC bilayers, leading to more frequent yet transient openings at lower temperatures.' - Complex conclusion, which is incompletely comprehensible from the results discussed. Please explain more about how temperature, lipid phase, and hydration are correlated and the functional relevance of that."

Reply: *Thank you, we agree with this point. We have now refined our hypothesis considering the new results from MD simulations of DSPC in nanodiscs. This new data revealed that lipids form an interdigitated phase by thinning the bilayer, and this in turn leads to compensation of the thickness mismatch between the expanded state EcMscL and standard DSPC bilayer thickness. Notably, this agrees with our experimental findings, informed by cryo-EM, PELDOR, and HDX-MS, and the process is not heavily temperature dependent.*

"Line 440: 'Indeed, during our MD simulations of both states, fewer lipids were observed penetrating the pockets in DSPC bilayers compared to DSPC.' – Please refer to an appropriate image. Also, please discuss any evidence that the authors might have seen from the MD simulations in support of the described 'lipid-moves-first' model."

Reply: *Thank you. We now provide a new figure and data in the Supporting Information to illustrate a mapping of the lipid pocket regions on EcMscL and calculated the lipid contacts (of the pocket residues) in the expanded and closed state simulations. We find that the lipid contacts are reduced in the expanded state, consistent with the lipid-moves-first model (please see relevant response elsewhere).*

"In Figure 5b, the authors need to specify the novel information on the gating mechanism obtained from the current study in relation to the existing literature."

Reply: *We provide the first high-resolution structure, resembling a closed (non-conducting) state of the first mechanosensitive channel ever to be discovered, and in an expanded state. This is highly novel, and a necessary step towards any mechanistic understanding. Further, our approach to use PELDOR as a "scanning tool", to identify functionally relevant channel states within the EcMscL ensemble and then inform subsequent structure acquisition by cryoEM is highly novel (and unique), and could be applied to a wide range of other membrane protein cases.*

Reviewer #4 (Remarks to the Author):

Hardman et al. present an extensive structural analysis of the structure of the E. coli MscL channel. The authors combine CryoEM, electron-electron double resonance spectroscopy and molecular dynamics simulations to characterize the closed and expanded states of the channel. The manuscript is written clearly, and the figures are of high quality. I outline below my questions and concerns:

Reply: *We are thankful to the reviewer for taking the time to assess our work and appreciate that they found our manuscript, including the figures, to be of a high standard.*

It is very interesting and unexpected that the authors observe expansion of the channel when reconstituted in DSPC lipids. However, given what is known in the literature about the phases

that saturated lipids such as DPPC (di-C16:0) and DSPC (di-C18:0) form below their transition temperatures, it seems highly unlikely that DSPC is in a gel phase in the lipid nanodiscs with MscL. The headgroup-to-headgroup distance (DHH) for DMPC in the fluid phase is ~36 A, while DHH = 44 A for gel phase DPPC (see Nagle et. al BBA 1469, 159, 2000). As such, DHH for DSPC in the gel phase would be even larger, which would be inconsistent with the ~25 A thickness of the transmembrane portion of the expanded EcMscL. The thickness of the membrane in the nanodiscs (Fig. 3a and d) is also consistent with a DHH ~25-30 A, which would not correspond to DSPC in the gel phase. Instead, the authors should consider that the DSPC lipids are forming an interdigitated phase, where the lipid tails become fully intercalated leading to significant reduction in the bilayer thickness (see Slater and Huang Prog. Lipid. Res. 27: 325, 1988; Seto et al. J Phys Soc JPN 2007; 76: 054602). The headgroup to headgroup thickness for fully interdigitated DPPC under high pressure is ~29 A (Braganza LF, Worcester DL. Biochemistry 1986; 25: 2591–6). Atomic force microscopy measurements on supported lipid bilayers of DPPC below the transition temperature have shown that lateral tension may also induce formation of the fully interdigitated phase (Vanegas et al. Langmuir 2010; 26: 10415–8). As such, it seems more likely that belt protein around the nanodisc is creating a tension that stabilizes the interdigitated phase of DSPC. I don't know if it would be possible to analyze the lipid tail and/or headgroup densities in the CryoEM maps for any signs of the interdigitated phase. For example, the area per lipid will be significantly larger compared to the fluid or gel phases.

Reply: *This is an excellent point and central to our new findings, which we also found very interesting. What we propose is (a) potential mechanism(s) to rationalise how we achieved in obtaining the expanded state, by inducing a membrane tension- sensitive transition of EcMscL. We therefore orthogonally tested our findings by employing two other experimental high resolution structural approaches, namely PELDOR and HDX-MS, under the same conditions. Both methods are consistent with the EM findings. We agree with the reviewer’s hypothesis, and we now amend the text to reflect this possibility. Further, following the reviewer’s suggestion, we have now reanalysed our previous MD trajectories (and run additional simulations) and we now conclude that lipid interdigitation (leading to bilayer thickness reduction), as well as the belt protein stabilises the interdigitated bilayer DSPC state (please see responses elsewhere).*

“Related to point 1, the authors will likely need to simulate the expanded EcMscL in the interdigitated phase of DSPC. This may need to be done under tension in order to stabilize this phase. I don’t see the point in simulating the protein under high temperature in DSPC as this does not match any of the other experiments or provide physiologically relevant information.”

Reply: *Again, this is an excellent point. We have now succeeded in stabilising the expanded EcMscL state under computational tension to investigate this phenomenon further. We did not do so previously, to be compatible with the experimental data, however, this is a great suggestion, which we will pursue in the future. We also cite relevant literature.*

“The observation that the loop region that connects TM2 to the CTD is fully extended and places the CTD 25 A below the protein is somewhat striking. The authors mention in lines 147-148 that this is likely due increased rigidity from the three prolines in the loop, but there's not much of a follow up to this. The snapshot of the MD simulation in Fig. 4e shows some flexibility in the loops. I think that the rigid nature of these loops should be assessed in more depth. Is it possible that the image classification procedure is somehow 'pre-selecting' conformations where the CTD is essentially aligned with the axis of the TM region? Could the PELDOR analysis be used to characterize the flexibility of the loop region? For example one could place probes on the lower part of TM2 and the top of CTD and use this to estimate the typical distance between the CTD from the membrane.”

Reply: *We thank the reviewer for their comment and suggestion. We have addressed this point in detail above (see response to reviewer 1). In a previous study, we placed a reporter on the top of the CTD of EcMscL (i.e., V120) and recorded high SNR and robust PELDOR traces in agreement with a fully formed and rigid CTD (Ackermann et al. 2017). While PELDOR measurements of loop regions can inform disorder-order transitions, the conformational flexibility often leads to prohibitively broad distributions.*

“Minor comment: With regards to the MD simulation parameters, the authors do not provide many details on the type of pressure coupling. I'm assuming that a semi-isotropic barostat was used, but this should be explicitly stated. For gel/interdigitated phase simulations the authors may need to use an anisotropic barostat in order to correctly model the membrane properties. The authors should also provide additional detail about the vdw/coulomb cutoffs and long-range electrostatic interactions for completeness and reproducibility. It was also no t clear what criteria was used to determine the length of the simulations? What determines convergence?”

Reply: *We thank the reviewer for their comments, we have now added all these details to the methods section of the main text. In brief, we have used both a semi-isotropic barostat (for bilayers) and an isotropic barostat (for the new nanodisc simulations we have included in the revision). Under these conditions we have still observed an interdigitated phase.*

Reviewer 1 Comments:

Reviewer: “All my concerns have been addressed appropriately. Thanks for the insightful replies.”

Reply: *We appreciate the thoroughness of the reviewer’s initial comments and are glad that they are now satisfied. Thank you.*

Reviewer 2 Comments:

Reviewer: “Overall, I think the revised manuscript is significantly improved. Both the other reviewers and are to be thanked for this work. The authors have performed a lot of additional work, new PELDOR measurements in native-like E. coli lipid nanodiscs and additional MD simulations. These new data help clarify an important point of the initial submission, namely whether the DSPC nanodisc condition reflects a meaningful shift in the conformational ensemble.”

Reply: *We appreciate the reviewer assessment that our manuscript is significantly improved, and we agree. Thank you.*

Reviewer: “I also appreciate that the authors have softened the interpretation of the DSPC structure. The manuscript now more clearly describes this conformation as an expanded, open-like, or sub-conducting intermediate rather than as a fully open MscL channel. The additional PELDOR data in E. coli polar lipids are very useful. They support the idea that the MSP1D1 scaffold alone does not force EcMscL into an expanded conformation, since the channel appears predominantly closed in a native-like lipid environment. The new MD simulations also provide a more plausible explanation for the behaviour of DSPC nanodiscs, especially through bilayer thinning and interdigitation. I still think that this remains a somewhat artificial condition but the authors now discuss this more transparently and no longer overstate it as a direct mimic of the fully tension-gated open state. I therefore think that the manuscript is totally suitable for publication.”

Reply: *We thank the reviewer for acknowledging the value of the additional PELDOR control measurements, new MD simulations, and our transparency.*

Reviewer: “[…] There are now both Supplementary Figures and Supporting Information, which makes the manuscript a bit confusing to navigate. It would be helpful if the authors could harmonize this, using either “Supporting” or “Supplementary”. Also, the information presented in the supporting information is not consistently referenced to in the main article file.

Reply: *We thank the reviewer for identifying an opportunity to harmonize the various supplementary materials. We have now shifted all Supporting Information materials (Supporting Fig(s) and Table(s).) into the Supplementary Figs. and Methods section(s) to streamline for the reader.*

Reviewer: “The caption of Supporting Figure 6 still mentions an “open” state instead of an “expanded” state.”

Reply: *Thank you for identifying this typo. We have now amended this to “expanded state”.*

Reviewer #3 (Remarks to the Author)

Reviewer: “The new experiments and analyses addressing my concerns about PELDOR data acquisition and analysis are highly welcome and significantly improve the coherence of the manuscript. However, a few information discussed in the authors' response are currently missing from the manuscript and supporting information. The authors are strongly encouraged incorporate these details into the final files.”

Reply: *We thank the reviewer for assessing the coherence of the manuscript to be significantly improved. We have now endeavoured to embed the content of the Supporting Information document into the Supplementary Figures and Methods Section(s). Furthermore, we have streamlined the in-text referencing to the Supplementary Figures and Tables to be more consistent.*

Reviewer: “Regarding the query on the modulation depth of the PELDOR data, authors state that that they performed PELDOR experiments from two distinct peaks of the

SEC profile to estimate the pentamer fraction and labeling efficiency. However, neither the SEC profile nor the PELDOR data for the second peak (peak 2) are presented."

Reply: *With respect to this point, we have now added both a representative SEC profile of EcMscL in DDM, indicating the two distinct peaks, and compare I79R1 DDM PELDOR data from peak(s) 1 and 2 in Supplementary Fig. 18.*

Reviewer: "For the determination of the distances from PELDOR data, now authors present output from the DeerLab program. Please briefly explain in the methods, how the multi-spin effects were accounted for during this analysis specific in the methods section. Authors mention that the output from the Consensus Deer Analyzer (CDA) is presented in the supporting information, which cannot be found and must be added."

Reply: *We appreciate the reviewer comment regarding the CDA outputs but note that this data was previously provided in Supporting Fig. 4-5 within the Supporting Information. These time-traces and distributions have now been transferred to the main-text Supplementary Fig. 7-8. Regarding the analysis in DeerLab, we note that implementation of power-scaling or post-processing methods to address multi-spin effects are not part of the current codebase. We utilized DeerLab to provide global fitting of technical replicates. While we appreciate that it is possible to first perform power-scaling on the raw traces inside the CDA, the loss of the negative time-points obfuscates determination of a zero-time during fitting within DeerLab. We therefore neglect these effects during DeerLab processing and rather process both datasets (technical replicates) without post-processing multi-spin suppression. We have added a statement to this effect in the methods section.*

Reviewer #4 (Remarks to the Author)

Reviewer: "While the authors have addressed many of the issues I brought up in my earlier review, I have some new questions/comments from the substantial changes/additions to the manuscript.

The subsection about the bilayer thickness and interdigitation (lines 439-455) feels like an afterthought at the end of the Results section. Given the importance of using DSPC in the nanodiscs to capture the expanded state of EcMscL, I think that the possible

mechanisms to why a long-chain, high melting T, lipid could induce a protein state characterized by a reduced membrane thickness should be presented early on. The results presented starting at lines 190 through 308 are completely focused on the protein features and ignore what's happening in the surrounding membrane environment."

Reply: *We thank the reviewer for their suggestion. We agree that the membrane environment and its structural properties are central to understanding the stabilization of the expanded EcMscL state in DSPC nanodiscs. Thus, we now moved the section entitled "Bilayer thickness and interdigitation in EcMscL gating mechanism" to the beginning of our MD simulations analysis and results. This reorganization introduces the membrane-thinning and interdigitation phenomena before discussing the protein structural changes and lipid-contact analysis, thereby providing the mechanistic framework for understanding how DSPC nanodiscs stabilize the expanded state.*

Reviewer: "The authors need to clearly state early on that the nanodiscs formed by DSPC cannot possibly be in the conventional gel or liquid crystalline phases as the thickness (~25 A) shown by the CryoEM maps suggests otherwise. E.g., in lines 342-343 the authors state "Although DSPC lipids form thicker bilayers than DMPC (3.6 nm vs 2.5 nm), our results identified two regions of increased deuterium uptake in DSPC, spanning residues 27-36 and 40-47 (Fig. 3h)." This statement hints at that there is something unusual going on with the DSPC nanodiscs, but at the same time the first part implies that the bilayer thickness of DSPC 'should' be larger. My point is that it's confusing and somewhat deceiving to present the results of the expanded state in DSPC assuming that it always forms a thick gel phase membrane and then at the end say nevermind it was likely an interdigitated phase all along!"

Reply: *We thank the reviewer for pointing this out. This remained as part of the initial explanation for channel expansion which we have readdressed considering new data and analysis during revision. As per Reviewer's suggestion we now revised the text accordingly and removed the statement which implies that the DSPC nanodiscs retain the thickness expected for a conventional gel-phase DSPC bilayer.*

Reviewer: "I think the authors should not refer to DSPC as gel-phase lipids (e.g., line 484) and instead use the term high melting T or high transition T lipids to avoid

confusion that DSPC always forms a gel phase at physiological or room temperatures."

Reply: *We thank the reviewer for raising this point. We agree that referring to DSPC simply as a "gel-phase lipid" may be misleading in the context of the present study, as the lipid phase behaviour is influenced by the local membrane environment and does not necessarily correspond to a conventional gel phase. To avoid potential confusion, we have now revised the text throughout the manuscript and refer to DSPC as a high-transition-temperature (high-Tm) lipid, where appropriate.*

Reviewer: "Additionally, the bilayer thickness and interdigitation subsection seems to only refer to the simulation results to support the idea that the DSPC nanodiscs are interdigitated, but the CryoEM maps also provide strong support for this given the observed hydrophobic thickness."

Reply: *We agree and now revised the manuscript to highlight that the reduced hydrophobic thickness observed in the cryoEM maps is consistent with the pronounced bilayer thinning and enhanced interdigitation observed in the DSPC nanodisc simulations. This agreement between the experimental and computational data further supports our interpretation that the DSPC nanodisc environment adopts a thin, interdigitated membrane state that facilitates stabilization of the expanded EcMscL conformation.*

Reviewer: "Given the time evolution of the channel in the MD simulations (e.g., Fig. Supp. 6), where the system may be transitioning from an expanded to a closed state over time. I don't think it is appropriate to make statements about the percent of time spent in a particular configuration or generate probabilities (e.g., lines 403-425) as this assumes that the simulations are exploring a well-sampled equilibrium. This would require running much longer simulations with multiple replicas. It's ok to describe the time evolution and make statements about how it only remains in a particular state for some period of time out of a long simulation, but presenting percentages and probabilities requires much more rigorous sampling."

Reply: *The observed time evolution suggests that some systems may undergo gradual relaxation from the initial expanded conformation, and therefore reporting percentages or probabilities could overstate the extent of conformational sampling achieved in the simulations. We now amend the reference to pore-opening probabilities in the revised manuscript. We have revised the corresponding sections to describe the time-dependent behaviour of the systems and the relative persistence of pore hydration and channel expansion during the simulations, without implying equilibrium sampling or population estimates. The revised text now focuses on the qualitative and comparative trends observed across the different membrane environments while avoiding over-interpretation of the simulation data.*

Reviewer: “The newly added paragraph at the end of the discussion section (lines 491-509) seems highly speculative about how lipid removal from crevices and the 'lipids move first' could be playing a role in the transition to the expanded state. The resolution of the CryoEM maps does not allow the authors to directly probe this hypothesis. The authors state in line 504 "Notably, the hydrophobic pockets lining the cytoplasmic side of EcMscL reduce in volume when the channel expands, suggesting they can accommodate fewer lipids in the expanded state (Fig. 4e-f)." Yet the correlation between the crevices not accommodating lipids in the expanded state does not have to be the cause for the channel to transition to this state.”

Reply: *The proposed interpretation is not based solely on the cryoEM data. Rather, it is supported by our new MD simulation analysis (thus the new addition), consistent with previous studies on other large conductance (i.e. MtMscL) and small conductance (i.e. EcMscS) MS channels . We have performed detailed lipid contact analysis on our expanded and closed EcMscL structures, clearly supportive of the lipid moves first model. Specifically, our MD simulations reveal reduced lipid contacts within the hydrophobic pockets (in accordance with cryoEM data, where pocket volume also decreases upon channel expansion) of the expanded compared to the closed EcMscL state (Fig 4). This analysis is consistent with the lipid-moves-first model.*

Reviewer: I believe there's a typo in line 494 "however, in the case of EcMscL51,100 and the MscS-like EcYnal101 which both have substantially higher tension activation thresholds compared to EcMscL," should the first be EcMscS?

Reply: *We thank the reviewer for identifying this typo. We have now corrected this in the revised manuscript.*
