# Supplementary material for: Structural basis of the gating mechanism of the large-conductance mechanosensitive channel from Escherichia coli
Source: Nat Commun. 2026 Aug 22;17:10777. doi: 10.1038/s41467-026-76193-0 (PMC13661626; doi:10.1038/s41467-026-76193-0)
Supplement: Supplementary file 3 [file 41467_2026_76193_MOESM3_ESM.pdf]

nature portfolio

Corresponding author(s): Christos Pliotas

Last updated by author(s): June 23, 2026

Reporting Summary

Nature Portfolio wishes to improve the reproducibility of the work that we publish. This form provides structure for consistency and transparency in reporting. For further information on Nature Portfolio policies, see our Editorial Policies and the Editorial Policy Checklist.

Please do not complete any field with "not applicable" or n/a. Refer to the help text for what text to use if an item is not relevant to your study. For final submission: please carefully check your responses for accuracy; you will not be able to make changes later.

Statistics

For all statistical analyses, confirm that the following items are present in the figure legend, table legend, main text, or Methods section.

n/a	Confirmed	
☐	☒	The exact sample size (n) for each experimental group/condition, given as a discrete number and unit of measurement
☐	☒	A statement on whether measurements were taken from distinct samples or whether the same sample was measured repeatedly
☐	☒	The statistical test(s) used AND whether they are one- or two-sided<br>*Only common tests should be described solely by name; describe more complex techniques in the Methods section.*
☒	☐	A description of all covariates tested
☒	☐	A description of any assumptions or corrections, such as tests of normality and adjustment for multiple comparisons
☐	☒	A full description of the statistical parameters including central tendency (e.g. means) or other basic estimates (e.g. regression coefficient) AND variation (e.g. standard deviation) or associated estimates of uncertainty (e.g. confidence intervals)
☐	☒	For null hypothesis testing, the test statistic (e.g. F, t, r) with confidence intervals, effect sizes, degrees of freedom and P value noted<br>*Give P values as exact values whenever suitable.*
☒	☐	For Bayesian analysis, information on the choice of priors and Markov chain Monte Carlo settings
☒	☐	For hierarchical and complex designs, identification of the appropriate level for tests and full reporting of outcomes
☒	☐	Estimates of effect sizes (e.g. Cohen's d, Pearson's r), indicating how they were calculated

Our web collection on statistics for biologists contains articles on many of the points above.

Software and code

Policy information about availability of computer code

Data collection	CryoEM data were collected on a Titan Krios with Thermo Fisher Scientific EPU software v3.8.<br>Molecular dynamics simulations were performed using NAMD 3.0.<br>In-silico modelling of distance distributions were performed in the MtsslSuite webserver. Raw PELDOR traces were analyzed using the DeerAnalysis2022-embedded ConsensusDeerAnalyzer (CDA) 2.0, and DeerLab.
Data analysis	CryoEM data were processed in CryoSPARC v4.5.3 and RELION-4.0. Model building was performed using Coot v0.9 and ISOLDE v1.6, and validated using PHENIX v1.17.1.3660. Molecular dynamics trajectories were analysed using VMD, CHAP, HOLE, Python v3.x, and custom analysis scripts, as described in the Methods.Calculation of Wasserstein distances, Bhattacharyya coefficients, and global fitting of PELDOR modulation depths to calculate pentameric EcMscL fraction and labeling efficiencies was performed in MATLAB. Analyses and custom scripts are described in the Methods section. Custom scripts are available from the corresponding author upon reasonable request.

For manuscripts utilizing custom algorithms or software that are central to the research but not yet described in published literature, software must be made available to editors and reviewers. We strongly encourage code deposition in a community repository (e.g. GitHub). See the Nature Portfolio guidelines for submitting code & software for further information.

Data

Policy information about availability of data

All manuscripts must include a data availability statement. This statement should provide the following information, where applicable:

- Accession codes, unique identifiers, or web links for publicly available datasets
- A description of any restrictions on data availability
- For clinical datasets or third party data, please ensure that the statement adheres to our policy

The EcMscL models are deposited in the Protein Data Bank (PDB) under the accession codes 28OA (DMPC), 28OB (DOPC) and 28OC (DSPC). The mass spectrometry proteomics data have been deposited to the ProteomeXchange Consortium via the PRIDE [1] partner repository with the dataset identifier PXD077332 (Reviewer Token: wkBmynq3fx0g). The EPR and the molecular dynamics (MD) analysis outputs generated in this study are provided in the Source Data file.

Field-specific reporting

Please select the one below that is the best fit for your research. If you are not sure, read the appropriate sections before making your selection.

☒ Life sciences ☐ Behavioural & social sciences ☐ Ecological, evolutionary & environmental sciences

For a reference copy of the document with all sections, see nature.com/documents/nr-reporting-summary-flat.pdf

Life sciences study design

All studies must disclose on these points even when the disclosure is negative.

Sample size	No humans or animals were included in this structural biology-focussed study, therefore sample size does not apply. The large number of micrographs obtained for the DSPC condition reflects the challenges posed in processing this heterogenous dataest.
Data exclusions	No data were excluded.
Replication	For the expanded-state DSPC structure, channel expasion was observed from samples prepared independently by cryoEM, PELDOR and HDX-MS.
Randomization	This non-clinical, biochemistry study was not related to randomisation as no humans or animals were included.
Blinding	Blinding was not relevant to our study as it is not an established practice in structural biology projects.

Reporting for specific materials, systems and methods

We require information from authors about some types of materials, experimental systems and methods used in many studies. Here, indicate whether each material, system or method listed is relevant to your study. If you are not sure if a list item applies to your research, read the appropriate section before selecting a response.

Materials & experimental systems

n/a	Involved in the study	
☒	☐	Antibodies
☒	☐	Eukaryotic cell lines
☒	☐	Palaeontology and archaeology
☒	☐	Animals and other organisms
☒	☐	Clinical data
☒	☐	Dual use research of concern
☒	☐	Plants

Methods

n/a	Involved in the study	
☒	☐	ChIP-seq
☒	☐	Flow cytometry
☒	☐	MRI-based neuroimaging

This checklist template is licensed under a Creative Commons Attribution 4.0 International License, which permits use, sharing, adaptation, distribution and reproduction in any medium or format, as long as you give appropriate credit to the original author(s) and the source, provide a link to the Creative Commons license, and indicate if changes were made. The images or other third party material in this article are included in the article's Creative Commons license, unless indicated otherwise in a credit line to the material. If material is not included in the article's Creative Commons license and your intended use is not permitted by statutory regulation or exceeds the permitted use, you will need to obtain permission directly from the copyright holder. To view a copy of this license, visit http://creativecommons.org/licenses/by/4.0/
