# Supplementary material for: Structural basis of the gating mechanism of the large-conductance mechanosensitive channel from Escherichia coli
Source: Nat Commun. 2026 Aug 22;17:10777. doi: 10.1038/s41467-026-76193-0 (PMC13661626; doi:10.1038/s41467-026-76193-0)
Supplement: Supplementary file 4 [file 41467_2026_76193_MOESM4_ESM.pdf]

Description of Additional Supplementary Files

Supplementary Video 1. Gating transition of EcMscL from the closed to the expanded (open-like) state. Channel expansion is characterised by the lateral tilting and displacement of TM1 and TM2, which significantly reduces the transmembrane thickness and drives a pore dilation from ~2 to ~8 Å. The animation was generated in ChimeraX by morphing between the DMPC- and DSPC-stabilised structures. Top pore and side views are presented.
