# Supplementary material for: Structural basis of the gating mechanism of the large-conductance mechanosensitive channel from Escherichia coli
Source: Nat Commun. 2026 Aug 22;17:10777. doi: 10.1038/s41467-026-76193-0 (PMC13661626; doi:10.1038/s41467-026-76193-0)
Supplement: Supplementary file 5 [file 41467_2026_76193_MOESM6_ESM.zip › Source Data/Underpinning Data Tables_EPR_EcMscL.docx]

Underpinning Data Tables – Hardman et al.,

| Figure | Panel | ID | Raw Data | Meta Data |
| --- | --- | --- | --- | --- |
| Main text; 2 | B | blue trace; grey trace | JW_011_260414_PELDOR_lambda_red_65scans.DSC;  JW_011_260414_PELDOR_lambda_red_65scans.DTA;  JW_010_260410_PELDOR_66scans.DSC;  JW_010_260410_PELDOR_66scans.DTA;  JW_010_260410_PELDOR_66scans_comparative_DEER_analysis.mat;  JW_011_260414_PELDOR_lambda_red_65scans_comparative_DEER_analysis.mat; | blue_trace.dat; I79R1_DDM_lambda_reduction.dat;  I79R1_DDM_nolambda_reduction.dat; |
| Main text; 2 | B | red trace; grey trace | 20260420_JW_113_PELDOR_40scans.DSC;  20260420_JW_113_PELDOR_40scans.DTA;  20260421_JW_EcMscL_I79R1_firstpeak_ND_Epe_lamda_redu.DSC;  20260421_JW_EcMscL_I79R1_firstpeak_ND_Epe_lamda_redu.DTA  20260420_JW_113_PELDOR_40scans_comparative_DEER_analysis.mat;  20260421_JW_EcMscL_I79R1_firstpeak_ND_Epe_lamda_redu_comparative_DEER_analysis.mat | red_trace.dat;  I79R1_EcPE_lambda_reduction.dat;  I79R1_EcPE_nolambda_reduction.dat |
| Main text; 2 | B | magenta trace; grey trace | 260426_XY_PELDOR_67scans.DSC;  260426_XY_PELDOR_67scans.DTA;  260426_XY_PELDOR_67scans_comparative_DEER_analysis.mat;  260427_JW_PELDOR_noreduction_35scans.DTA;  260427_JW_PELDOR_noreduction_35scans.DSC;  260427_JW_PELDOR_noreduction_35scans_comparative_DEER_analysis.mat | magenta_trace.dat;  I79R1_D1DSPC_no_reduction.dat;  I79R1_D1DSPC_lambda_reduction.dat |
| Main text; 2 | B | pink fill | - | pink_fill.dat |
| Main text; 2 | B | teal fill | - | teal_fill.dat |
| Main text; 2 | C | blue trace; grey fill | JW_014_260416_PELDOR_lambdared_35scans.DSC;  JW_014_260416_PELDOR_lambdared_35scans.DTA;  JW_012_260414_PELDOR_33scans.DSC;  JW_012_260414_PELDOR_33scans.DTA;  JW_014_260416_PELDOR_lambdared_35scans_comparative_DEER_analysis.mat;  JW_012_260414_PELDOR_33scans_comparative_DEER_analysis.mat; | blue_trace.dat;  M94R1_DDM_lambda_reduced.dat;  M94R1_DDM_no_lambdared.dat |
| Main text; 2 | C | red trace; grey fill | 20260419_JW_112_PELDOR_lambda_red_75scans.DSC;  20260419_JW_112_PELDOR_lambda_red_75scans.DTA;  20260419_JW_112_PELDOR_lambda_red_75scans_comparative_DEER_analysis.mat;  20260416_XY_PELDOR_32scans.DSC;  20260416_XY_PELDOR_32scans.DTA;  20260416_XY_PELDOR_32scans_comparative_DEER_analysis.mat | red_trace.dat;  M94R1_EcPE_no_lambda_reduced.dat;  M94R1_EcPE_lambda_reduced.dat |
| Main text; 2 | C | magenta trace; grey fill | 20260424_XY_EcMscL_M94R1_MSP1D1_DSPC_lamda_42scans.DSC;  20260424_XY_EcMscL_M94R1_MSP1D1_DSPC_lamda_42scans.DTA;  20260424_XY_EcMscL_M94R1_MSP1D1_DSPC_lamda_42scans_comparative_DEER_analysis.mat  260422_XY_112_PELDOR_61scans.DSC;  260422_XY_112_PELDOR_61scans.DTA;  260422_XY_112_PELDOR_61scans_comparative_DEER_analysis.mat | magenta_trace.dat;  M94R1_DSPCD1_no_lambda_reduced.dat;  M94R1_DSPCD1_lambda_reduced.dat |
| Main text; 2 | C | pink fill | - | pink_fill.dat |
| Main text; 2 | C | teal fill | - | teal_fill.dat |
| Supplementary; 5 | A; left | blue trace | 20241129_170727860_EcMscL_I79C_DDM_20241129_result.DSC;  20241129_170727860_EcMscL_I79C_DDM_20241129_result.DTA; | left_panel_blue_trace.dat |
| Supplementary; 5 | A; left | red trace | 20260408_131844905_20260408_I79R1_MSP1D1_Ecolipolar_result.DSC;  20260408_131844905_20260408_I79R1_MSP1D1_Ecolipolar_result.DTA | left_panel_red_trace.dat |
| Supplementary; 5 | A; left | magenta trace | 20241203_131226633_EcMscL_I79R1_DSPC_MSPD1_20241203_result.DSC;  20241203_131226633_EcMscL_I79R1_DSPC_MSPD1_20241203_result.DTA | left_panel_magenta_trace.dat |
| Supplementary; 5 | A; left | cyan trace | 20241217_150623132_EcMscL_I79R1_E3D1_DSPC_20241217_result.DSC;  20241217_150623132_EcMscL_I79R1_E3D1_DSPC_20241217_result.DTA | left_panel_cyan_trace.dat |
| Supplementary; 5 | A; right | blue trace | 20241128_135029707_EcMscL_M94C_DDM_20241128_result.DSC;  20241128_135029707_EcMscL_M94C_DDM_20241128_result.DTA | right_panel_blue_trace.dat |
| Supplementary; 5 | A; right | red trace | 20260414_161956116_20260414_EcMscL_M94R1_MSP1D1_Ecolipolar_result.DSC;  20260414_161956116_20260414_EcMscL_M94R1_MSP1D1_Ecolipolar_result.DTA | right_panel_red_trace.dat |
| Supplementary; 5 | A; right | magenta trace | 20250116_135153803_20250116_EcMscL_M94R1_D1_DSPC_result.DSC;  20250116_135153803_20250116_EcMscL_M94R1_D1_DSPC_result.DTA | right_panel_magenta_trace.dat |
| Supplementary; 5 | A; right | cyan trace | 20241217_142531020_EcMscL_M94R1_E3D1_DSPC_20241217_result.DSC;  20241217_142531020_EcMscL_M94R1_E3D1_DSPC_20241217_result.DTA | right_panel_cyan_trace.dat |
| Supplementary; 5 | B; left | blue trace; black trace; upper | JW_011_260414_PELDOR_lambda_red_65scans.DSC;  JW_011_260414_PELDOR_lambda_red_65scans.DTA;  JW_011_260414_PELDOR_lambda_red_65scans_comparative_DEER_analysis.mat | left_panel_upper_blue_trace.dat |
| Supplementary; 5 | B; left | blue trace; black trace; lower | JW_010_260410_PELDOR_66scans.DSC;  JW_010_260410_PELDOR_66scans.DTA;  JW_010_260410_PELDOR_66scans_comparative_DEER_analysis.mat | left_panel_lower_blue_trace.dat |
| Supplementary; 5 | B; centre | red trace; black trace; upper | 20260421_JW_EcMscL_I79R1_firstpeak_ND_Epe_lamda_redu.DSC;  20260421_JW_EcMscL_I79R1_firstpeak_ND_Epe_lamda_redu.DTA;  20260421_JW_EcMscL_I79R1_firstpeak_ND_Epe_lamda_redu_comparative_DEER_analysis.mat | centre_panel_upper_red_trace.dat |
| Supplementary; 5 | B; centre | red trace; black trace; lower | 20260420_JW_113_PELDOR_40scans.DSC;  20260420_JW_113_PELDOR_40scans.DTA;  20260420_JW_113_PELDOR_40scans_comparative_DEER_analysis.mat | centre_panel_lower_red_trace.dat |
| Supplementary; 5 | B; right | magenta trace; black trace; upper | 260426_XY_PELDOR_67scans.DSC;  260426_XY_PELDOR_67scans.DTA;  260426_XY_PELDOR_67scans_comparative_DEER_analysis.mat | right_panel_upper_magenta_trace.dat |
| Supplementary; 5 | B; right | magenta trace; black trace; lower | 260427_JW_PELDOR_noreduction_35scans.DSC;  260427_JW_PELDOR_noreduction_35scans.DTA;  260427_JW_PELDOR_noreduction_35scans_comparative_DEER_analysis.mat | right_panel_upper_magenta_trace.dat |
| Supplementary; 5 | C; left | blue trace; black trace; upper | JW_014_260416_PELDOR_lambdared_35scans.DSC;  JW_014_260416_PELDOR_lambdared_35scans.DTA;  JW_014_260416_PELDOR_lambdared_35scans_comparative_DEER_analysis.mat | left_panel_upper_blue_trace.dat |
| Supplementary; 5 | C; left | blue trace; black trace; lower | JW_012_260414_PELDOR_33scans.DSC;  JW_012_260414_PELDOR_33scans.DTA;  JW_012_260414_PELDOR_33scans_comparative_DEER_analysis.mat | left_panel_lower_blue_trace.dat |
| Supplementary; 5 | C; centre | red trace; black trace; upper | 20260419_JW_112_PELDOR_lambda_red_75scans.DSC;  20260419_JW_112_PELDOR_lambda_red_75scans.DTA;  20260419_JW_112_PELDOR_lambda_red_75scans_comparative_DEER_analysis.mat | centre_panel_upper_red_trace.dat |
| Supplementary; 5 | C; centre | red trace; black trace; lower | 20260416_XY_PELDOR_32scans.DSC;  20260416_XY_PELDOR_32scans.DTA;  20260416_XY_PELDOR_32scans_comparative_DEER_analysis.mat | centre_panel_lower_red_trace.dat |
| Supplementary; 5 | C; right | magenta trace; black trace; upper | 20260424_XY_EcMscL_M94R1_MSP1D1_DSPC_lamda_42scans.DSC;  20260424_XY_EcMscL_M94R1_MSP1D1_DSPC_lamda_42scans.DTA  20260424_XY_EcMscL_M94R1_MSP1D1_DSPC_lamda_42scans_comparative_DEER_analysis.mat | right_panel_upper_magenta_trace.dat |
| Supplementary; 5 | C; right | magenta trace; black trace; lower | 260422_XY_112_PELDOR_61scans.DSC;  260422_XY_112_PELDOR_61scans.DTA;  260422_XY_112_PELDOR_61scans_comparative_DEER_analysis.mat | right_panel_lower_magenta_trace.dat |
| Supplementary; 6 | A | cyan trace; black trace; upper | 260430_JW_PELDOR_lambda_reduced_60scans.DSC;  260430_JW_PELDOR_lambda_reduced_60scans.DTA;  260430_JW_PELDOR_lambda_reduced_60scans_comparative_DEER_analysis.mat | upper_cyan_trace.dat |
| Supplementary; 6 | A | cyan trace; black trace; lower | 260428_XY_PELDOR_noreduction.DSC;  260428_XY_PELDOR_noreduction.DTA;  260428_XY_PELDOR_noreduction_comparative_DEER_analysis.mat | lower_cyan_trace.dat |
| Supplementary; 6 | B | cyan trace; black trace | YM_013_250210_DEER.DSC;  YM_013_250210_DEER.DTA;  YM_013_250210_DEER_comparative_DEER_analysis.mat | cyan_trace.dat |
| Supplementary; 6 | C | cyan trace; grey fill | 260428_XY_PELDOR_noreduction.DSC;  260428_XY_PELDOR_noreduction.DTA;  260428_XY_PELDOR_noreduction_comparative_DEER_analysis.mat  260430_JW_PELDOR_lambda_reduced_60scans.DSC;  260430_JW_PELDOR_lambda_reduced_60scans.DTA;  260430_JW_PELDOR_lambda_reduced_60scans_comparative_DEER_analysis.mat | cyan_trace.dat |
| Supplementary; 6 | C | pink fill | - | pink_fill.dat |
| Supplementary; 6 | C | teal fill | - | teal_fill.dat |
| Supplementary; 6 | D | cyan trace; grey fill | YM_013_250210_DEER.DSC;  YM_013_250210_DEER.DTA;  YM_013_250210_DEER_comparative_DEER_analysis.mat | cyan_trace.dat |
| Supplementary; 6 | D | pink fill | - | pink_fill.dat |
| Supplementary; 6 | D | teal fill | - | teal_fill.dat |
| Supplementary; 7 | A | blue trace; black trace; grey trace | JW_011_260414_PELDOR_lambda_red_65scans.DSC;  JW_011_260414_PELDOR_lambda_red_65scans.DTA;  JW_011_260414_PELDOR_lambda_red_65scans_comparative_DEER_analysis.mat | blue_trace.dat |
| Supplementary; 7 | A | red trace; black trace; grey trace | 20260421_JW_EcMscL_I79R1_firstpeak_ND_Epe_lamda_redu.DSC;  20260421_JW_EcMscL_I79R1_firstpeak_ND_Epe_lamda_redu.DTA;  20260421_JW_EcMscL_I79R1_firstpeak_ND_Epe_lamda_redu_comparative_DEER_analysis.mat | red_trace.dat |
| Supplementary; 7 | A | magenta trace; black trace; grey trace | 260426_XY_PELDOR_67scans.DSC;  260426_XY_PELDOR_67scans.DTA;  260426_XY_PELDOR_67scans_comparative_DEER_analysis.mat | magenta_trace.dat |
| Supplementary; 7 | A | cyan trace; black trace; grey trace | 260430_JW_PELDOR_lambda_reduced_60scans.DSC;  260430_JW_PELDOR_lambda_reduced_60scans.DTA;  260430_JW_PELDOR_lambda_reduced_60scans_comparative_DEER_analysis.mat | cyan_trace.dat |
| Supplementary; 7 | B | blue trace; black trace; grey trace | JW_014_260416_PELDOR_lambdared_35scans.DSC;  JW_014_260416_PELDOR_lambdared_35scans.DTA;  JW_014_260416_PELDOR_lambdared_35scans_comparative_DEER_analysis.mat | blue_trace.dat |
| Supplementary; 7 | B | red trace; black trace; grey trace | 20260419_JW_112_PELDOR_lambda_red_75scans.DSC;  20260419_JW_112_PELDOR_lambda_red_75scans.DTA;  20260419_JW_112_PELDOR_lambda_red_75scans_comparative_DEER_analysis.mat | red_trace.dat |
| Supplementary; 7 | B | magenta trace; black trace; grey trace | 20260424_XY_EcMscL_M94R1_MSP1D1_DSPC_lamda_42scans.DSC;  20260424_XY_EcMscL_M94R1_MSP1D1_DSPC_lamda_42scans.DTA  20260424_XY_EcMscL_M94R1_MSP1D1_DSPC_lamda_42scans_comparative_DEER_analysis.mat | magenta_trace.dat |
| Supplementary; 7 | C | blue trace; grey fill | JW_011_260414_PELDOR_lambda_red_65scans.DSC;  JW_011_260414_PELDOR_lambda_red_65scans.DTA;  JW_011_260414_PELDOR_lambda_red_65scans_comparative_DEER_analysis.mat | blue_trace.dat |
| Supplementary; 7 | C | red trace; grey fill | 20260421_JW_EcMscL_I79R1_firstpeak_ND_Epe_lamda_redu.DSC;  20260421_JW_EcMscL_I79R1_firstpeak_ND_Epe_lamda_redu.DTA;  20260421_JW_EcMscL_I79R1_firstpeak_ND_Epe_lamda_redu_comparative_DEER_analysis.mat | red_trace.dat |
| Supplementary; 7 | C | magenta trace; grey fill | 260426_XY_PELDOR_67scans.DSC;  260426_XY_PELDOR_67scans.DTA;  260426_XY_PELDOR_67scans_comparative_DEER_analysis.mat | magenta_trace.dat |
| Supplementary; 7 | C | cyan trace; grey fill | 260430_JW_PELDOR_lambda_reduced_60scans.DSC;  260430_JW_PELDOR_lambda_reduced_60scans.DTA;  260430_JW_PELDOR_lambda_reduced_60scans_comparative_DEER_analysis.mat | cyan_trace.dat |
| Supplementary; 7 | C | pink fill | - | pink_fill.dat |
| Supplementary; 7 | C | teal fill | - | teal_fill.dat |
| Supplementary; 7 | D | blue trace; grey fill | JW_014_260416_PELDOR_lambdared_35scans.DSC;  JW_014_260416_PELDOR_lambdared_35scans.DTA;  JW_014_260416_PELDOR_lambdared_35scans_comparative_DEER_analysis.mat | blue_trace.dat |
| Supplementary; 7 | D | red trace; grey fill | 20260419_JW_112_PELDOR_lambda_red_75scans.DSC;  20260419_JW_112_PELDOR_lambda_red_75scans.DTA;  20260419_JW_112_PELDOR_lambda_red_75scans_comparative_DEER_analysis.mat | red_trace.dat |
| Supplementary; 7 | D | magenta trace; grey fill | 20260424_XY_EcMscL_M94R1_MSP1D1_DSPC_lamda_42scans.DSC;  20260424_XY_EcMscL_M94R1_MSP1D1_DSPC_lamda_42scans.DTA  20260424_XY_EcMscL_M94R1_MSP1D1_DSPC_lamda_42scans_comparative_DEER_analysis.mat | magenta_trace.dat |
| Supplementary; 7 | D | pink fill | - | pink_fill.dat |
| Supplementary; 7 | D | teal fill | - | teal_fill.dat |
| Supplementary; 8 | A | blue trace; black trace; grey trace | JW_010_260410_PELDOR_66scans.DSC;  JW_010_260410_PELDOR_66scans.DTA;  JW_010_260410_PELDOR_66scans_comparative_DEER_analysis.mat | blue_trace.dat |
| Supplementary; 8 | A | red trace; black trace; grey trace | 20260420_JW_113_PELDOR_40scans.DSC;  20260420_JW_113_PELDOR_40scans.DTA;  20260420_JW_113_PELDOR_40scans_comparative_DEER_analysis.mat | red_trace.dat |
| Supplementary; 8 | A | magenta trace; black trace; grey trace | 260427_JW_PELDOR_noreduction_35scans.DSC;  260427_JW_PELDOR_noreduction_35scans.DTA;  260427_JW_PELDOR_noreduction_35scans_comparative_DEER_analysis.mat | magenta_trace.dat |
| Supplementary; 8 | A | cyan trace; black trace; grey trace | 260428_XY_PELDOR_noreduction.DSC;  260428_XY_PELDOR_noreduction.DTA;  260428_XY_PELDOR_noreduction_comparative_DEER_analysis.mat | cyan_trace.dat |
| Supplementary; 8 | B | blue trace; black trace; grey trace | JW_012_260414_PELDOR_33scans.DSC;  JW_012_260414_PELDOR_33scans.DTA;  JW_012_260414_PELDOR_33scans_comparative_DEER_analysis.mat | blue_trace.dat |
| Supplementary; 8 | B | red trace; black trace; grey trace | 20260416_XY_PELDOR_32scans.DSC;  20260416_XY_PELDOR_32scans.DTA;  20260416_XY_PELDOR_32scans_comparative_DEER_analysis.mat | red_trace.dat |
| Supplementary; 8 | B | magenta trace; black trace; grey trace | 260422_XY_112_PELDOR_61scans.DSC;  260422_XY_112_PELDOR_61scans.DTA;  260422_XY_112_PELDOR_61scans_comparative_DEER_analysis.mat | magenta_trace.dat |
| Supplementary; 8 | B | cyan trace; black trace; grey trace | YM_013_250210_DEER.DSC;  YM_013_250210_DEER.DTA;  YM_013_250210_DEER_comparative_DEER_analysis.mat | cyan_trace.dat |
| Supplementary; 8 | C | blue trace; grey fill | JW_010_260410_PELDOR_66scans.DSC;  JW_010_260410_PELDOR_66scans.DTA;  JW_010_260410_PELDOR_66scans_comparative_DEER_analysis.mat | blue_trace.dat |
| Supplementary; 8 | C | red trace; grey fill | 20260420_JW_113_PELDOR_40scans.DSC;  20260420_JW_113_PELDOR_40scans.DTA;  20260420_JW_113_PELDOR_40scans_comparative_DEER_analysis.mat | red_trace.dat |
| Supplementary; 8 | C | magenta trace; grey fill | 260427_JW_PELDOR_noreduction_35scans.DSC;  260427_JW_PELDOR_noreduction_35scans.DTA;  260427_JW_PELDOR_noreduction_35scans_comparative_DEER_analysis.mat | magenta_trace.dat |
| Supplementary; 8 | C | cyan trace; grey fill | 260428_XY_PELDOR_noreduction.DSC;  260428_XY_PELDOR_noreduction.DTA;  260428_XY_PELDOR_noreduction_comparative_DEER_analysis.mat | cyan_trace.dat |
| Supplementary; 8 | C | pink fill | - | pink_fill.dat |
| Supplementary; 8 | C | teal fill | - | teal_fill.dat |
| Supplementary; 8 | D | blue trace; grey fill | JW_012_260414_PELDOR_33scans.DSC;  JW_012_260414_PELDOR_33scans.DTA;  JW_012_260414_PELDOR_33scans_comparative_DEER_analysis.mat | blue_trace.dat |
| Supplementary; 8 | D | red trace; grey fill | 20260416_XY_PELDOR_32scans.DSC;  20260416_XY_PELDOR_32scans.DTA;  20260416_XY_PELDOR_32scans_comparative_DEER_analysis.mat | red_trace.dat |
| Supplementary; 8 | D | magenta trace; grey fill | 260422_XY_112_PELDOR_61scans.DSC;  260422_XY_112_PELDOR_61scans.DTA;  260422_XY_112_PELDOR_61scans_comparative_DEER_analysis.mat | magenta_trace.dat |
| Supplementary; 8 | D | cyan trace; grey fill | YM_013_250210_DEER.DSC;  YM_013_250210_DEER.DTA;  YM_013_250210_DEER_comparative_DEER_analysis.mat | cyan_trace.dat |
| Supplementary; 8 | D | pink fill | - | pink_fill.dat |
| Supplementary; 8 | D | teal fill | - | teal_fill.dat |
| Supplementary; 17 | A | red trace; black trace | JW_010_260410_PELDOR_66scans.DSC;  JW_010_260410_PELDOR_66scans.DTA;  JW_010_260410_PELDOR_66scans_comparative_DEER_analysis.mat | red_trace.dat |
| Supplementary; 17 | A | blue trace; black trace | 20260410_XY_EcMscL_I79R1_0.05DDM_4us_29scans.DSC;  20260410_XY_EcMscL_I79R1_0.05DDM_4us_29scans.DTA;  20260410_XY_EcMscL_I79R1_0.05DDM_4us_29scans_comparative_DEER_analysis.mat | blue_trace.dat |
| Supplementary; 17 | B | black trace | - | black_trace.dat |
